# Mechanisms Underlying Hypoxia Tolerance in *Drosophila melanogaster*: *hairy* as a Metabolic Switch

**DOI:** 10.1371/journal.pgen.1000221

**Published:** 2008-10-17

**Authors:** Dan Zhou, Jin Xue, James C. K. Lai, Nicholas J. Schork, Kevin P. White, Gabriel G. Haddad

**Affiliations:** 1 Departments of Pediatrics (Section of Respiratory Medicine) and Neuroscience, University of California San Diego, La Jolla, California, United States of America; 2 Rady Children's Hospital – San Diego, San Diego, California, United States of America; 3 College of Pharmacy, Idaho State University, Pocatello, Idaho, United States of America; 4 Department of Molecular and Experimental Medicine, The Scripps Research Institute, La Jolla, California, United States of America; 5 Institute for Genomics and Systems Biology, The University of Chicago, Chicago, Illinois, United States of America; 6 Departments of Human Genetics and Ecology and Evolution, The University of Chicago, Chicago, Illinois, United States of America; University of California San Francisco, United States of America

## Abstract

Hypoxia-induced cell injury has been related to multiple pathological conditions. In order to render hypoxia-sensitive cells and tissues resistant to low O_2_ environment, in this current study, we used *Drosophila melanogaster* as a model to dissect the mechanisms underlying hypoxia-tolerance. A *D. melanogaster* strain that lives perpetually in an extremely low-oxygen environment (4% O_2_, an oxygen level that is equivalent to that over about 4,000 m above Mt. Everest) was generated through laboratory selection pressure using a continuing reduction of O_2_ over many generations. This phenotype is genetically stable since selected flies, after several generations in room air, survive at this low O_2_ level. Gene expression profiling showed striking differences between tolerant and naïve flies, in larvae and adults, both quantitatively and qualitatively. Up-regulated genes in the tolerant flies included signal transduction pathways (e.g., Notch and Toll/Imd pathways), but metabolic genes were remarkably down-regulated in the larvae. Furthermore, a different allelic frequency and enzymatic activity of the triose phosphate isomerase (TPI) was present in the tolerant versus naïve flies. The transcriptional suppressor, *hairy*, was up-regulated in the microarrays and its binding elements were present in the regulatory region of the specifically down-regulated metabolic genes but not others, and mutations in *hairy* significantly reduced hypoxia tolerance. We conclude that, the hypoxia-selected flies: (a) altered their gene expression and genetic code, and (b) coordinated their metabolic suppression, especially during development, with *hairy* acting as a metabolic switch, thus playing a crucial role in hypoxia-tolerance.

## Author Summary

Hypoxia-induced injury has been related to multiple pathological conditions. In order to render mammalian cells and tissues resistant to low O_2_ environment, we wished to first understand the mechanisms underlying hypoxia-tolerance in resistant animals. Therefore, we generated a *D. melanogaster* strain that is tolerant to severe hypoxic conditions through long-term experimental selection. Several adaptive changes were identified in the hypoxia-selected flies that included up-regulation of multiple signal transduction pathways (such as Notch pathway, Insulin pathway, EGF receptor pathway, and Toll/Imd pathway), modulation of cellular respiration enzymes, and polymorphic differences in metabolic enzymes (such as TPI). While we believe that multiple pathways contribute to the hypoxia-tolerant trait in this *Drosophila* strain, we demonstrate that hairy-mediated metabolic suppression is a critical mechanism for reducing the mismatch between supply and demand of O_2_.

## Introduction

Mammalian tissues experience a reduction in oxygen delivery at high altitude or during certain disease states, such as myocardial infarction and stroke. In order to survive, cells, tissues and organisms have developed various strategies to adapt to such O_2_ limited condition. There are indeed major differences between different organisms and cells in their ability to survive reduced environmental O_2_. For example, turtle neurons are very tolerant to low oxygen and can survive without O_2_ for hours and days [1],[2]. In contrast, mammalian neurons are very sensitive to reduced oxygen and cannot survive for even minutes under similar conditions. However, the mechanisms underlying survival in such extreme hypoxic conditions are not clear at present, in spite of the fact that there have been a number of interesting observations in this regard in the past few decades. For instance, it has been demonstrated that a number of hypoxia-tolerant animals (e.g. *Pseudemys scripta* and *Crucian Carp*) reduce their O_2_ consumption during hypoxia in such a way to minimize the mismatch between O_2_ supply and demand [3]–[5]. Similar phenomena was also observed in *Drosophila melanogaster* [6],[7] and in newborn mammals [8],[9]. Many questions, however, remain unsolved. For instance, we do not have an adequate understanding of the mechanisms that are responsible for reducing metabolic rate during low O_2_ conditions; and similarly, the mechanisms that are responsible for coordinating the suppression of these metabolic processes are still largely unknown.

In the early 1990s, we discovered that the fruit fly, *Drosophila melanogaster*, is tolerant to *acute* anoxia (zero mmHg O_2_). Flies can sustain such environment for a *few hours* without any evidence of injury [6]. Since a) *Drosophila* has been demonstrated to be a powerful genetic model for human diseases [10]–[14] and b) many biochemical and genetic pathways are highly conserved between *Drosophila* and mammals, we used *Drosophila* in the current study to explore the mechanisms underlying tolerance to long-term hypoxia. We first generated a *Drosophila melanogaster* strain that can live *perpetually* (i.e., from generation to generation) in severe, normally lethal, hypoxic conditions. To better understand the mechanisms underlying this remarkable hypoxia tolerance, we used cDNA microarrays containing 13,061 predicted or known genes (∼90% genes in the genome) to examine the differences in gene expression profiles between the hypoxia-selected (AF) and naïve control (NF) flies [15]–[17]. We performed these studies in both larvae and adults to determine gene expression as a function of development. Furthermore, we used a combination of bioinformatic, molecular and genetic strategies to investigate the role of specific genes in hypoxia tolerance.

## Results

### Experimental Selection of Hypoxia-Tolerant *Drosophila melanogaster*

**Figure 1:**
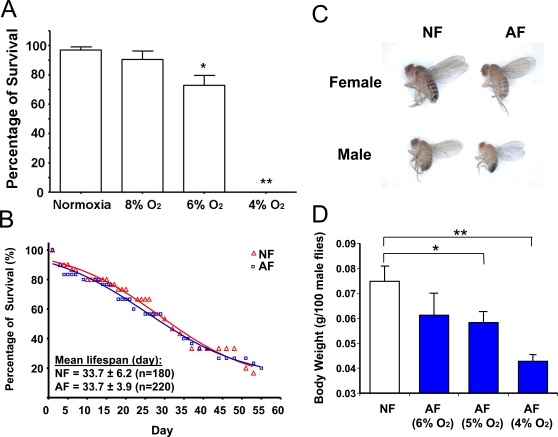
Phenotypic changes in hypoxia-selected *Drosophila melanogaster*. A) Survival rate of parental *Drosophila melanogaster* population in hypoxic conditions. The survival rates of the parental *Drosophila melanogaster* population were determined in hypoxic conditions, i.e., 8%, 6% and 4% oxygen. Data were presented as means±SD (* p<0.05, **p<0.01; student's t-test). The parental population survived well at 8% O_2_ but their survival rate was reduced at 6%; and 4% O_2_ was lethal. B) Lifespan of NF and AF. No significant alteration in lifespan was found in AF flies (p>0.05, student's t-test) at 25°C in normoxia. Survivorship curves were analyzed with GraphPad prism software (San Diego, CA) to calculate the mean lifespan. C) and D) Body size and weight in AF. The body size and weight of AF were significantly reduced, as compared to NF. Data were presented as means±SD (* p<0.05, **p<0.01; student's t-test).

Twenty-seven wild-type isogenic lines constituted the parental population that we used for long-term experimental selection of a *Drosophila melanogaster* strain. At baseline, there was significant variability in hypoxia tolerance among these 27 lines, as determined by eclosion rate under 5% O_2_ and recovery time from anoxic stupor [17]. In order to determine the level of O_2_ at which to initiate the long-term selection experiment, we performed a pilot study by culturing F1 embryos of the parental flies under different levels of hypoxia (e.g., 8%, 6% or 4% O_2_) (Figure 1A). We found that their survival rate was reduced at 6% O_2_, and no adult flies were actually obtained at 4% O_2_. At 8% O_2_, however, the majority of embryos (>80%) completed their development and reached the adult stage. Therefore, hypoxia selection was initiated at 8% O_2_, an O_2_ level in which flies can develop throughout their life cycle. The O_2_ concentration was then gradually decreased by ∼1% every 3 to 5 generations to maintain the selection pressure (except for the transition between 5% and 4% which necessitated a much longer time). By the 13^th^ generation, flies were able to complete development and perpetually live in 5% O_2_; and by the 32^nd^ generation, the AF flies could even live perpetually under a severer level (4% of O_2_), a lethal condition for NF. We hypothesized that this is due to, at least partially, newly occurring mutations or recombination and selection of favorable alleles in the AF population. To test this hypothesis, a subset of embryos obtained from selected flies were collected and cultured under *normoxia* for several consecutive generations. After 8 generations in normoxia, AF were re-introduced into the lethal hypoxic environment (4% O_2_), and again, the majority (>80%) of the flies completed their development and could be maintained in this extreme condition perpetually. This result strongly suggested that the hypoxia-tolerance in the selected flies is a heritable trait.

Several remarkable phenotypic changes were observed in the hypoxia-selected flies (AF). As described previously [17], the AF flies have a shortened recovery time from anoxia-induced stupor [6], consume more oxygen in hypoxia, and show a significant reduction in body weight and size. We also demonstrated that this reduction in size is due to the reduction in both cell number and cell size [17]. In the hypoxia chambers, at 5% O_2_ levels, adult flies had significantly decreased body weight: the decrease in male body weight was about 25% and was further reduced to about 40% under 4% O_2_ (Figure 1C and 1D). Interestingly, the reduction in body weight and size were reversed to normal when the AF embryos were grown under normoxia. Life span was also studied in our selected and naïve flies. Hypoxia-selection did not affect lifespan (Figure 1B).

### Whole Genome Expression Profiles of Hypoxia-Selected *Drosophila melanogaster*

**Figure 2:**
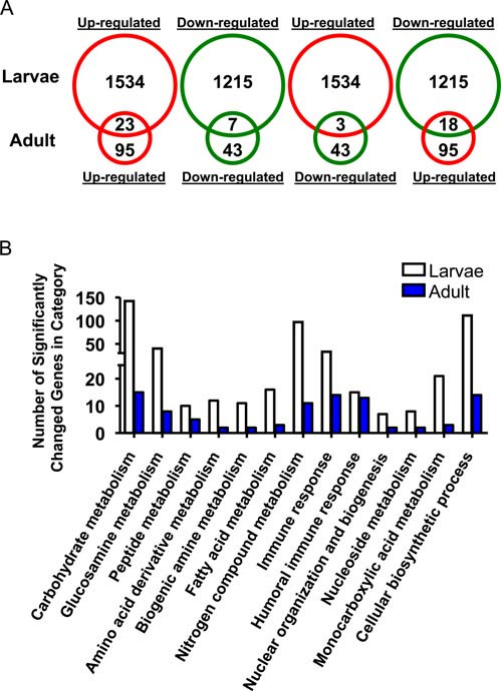
Gene expression profiles in hypoxia-selected *Drosophila melanogaster* in larvae and adult flies. A) Comparison of gene expression alterations in hypoxia-selected *Drosophila melanogaster* in the larva and adult fly. Expression levels of 2749 genes (1534 up- and 1215 down-regulated) in larvae and 138 genes (95 up- and 43 down-regulated) in the adult were significantly altered in AF (fold change>1.5 and q<0.05). Red: up-regulation; Green: down-regulation. B) Summary of significantly altered biological processes in larvae and adult hypoxia-selected *Drosophila melanogaster* (p<0.05).

Global gene expression profiles were examined in hypoxia-selected *Drosophila melanogaster* in the 3^rd^ instar larval stage and in adults using cDNA microarrays that contained 13,061 known or predicted genes of the *Drosophila melanogaster* genome [16],[18]. After analyzing the data sets with a significant cutoff of >1.5 fold difference and a false discovery rate (FDR) of <0.05 [19], 2749 genes (1534 up- and 1215 down-regulated) were significantly altered in the larval stage, but only 138 genes (∼20 times less than those in larvae) met this criteria (95 up- and 43 down-regulated) in the adult. The complete list of differentially regulated genes is detailed in Tables S1 and S2. Among them, 51 genes were found to be altered in both larval and adult stages with 23 up-regulated and 7 down-regulated genes (Figure 2A). Interestingly, most of the commonly up-regulated genes encode proteins that are related to immunity, and the majority of the commonly down-regulated genes encode proteins that are related to metabolism.

**Figure 3:**
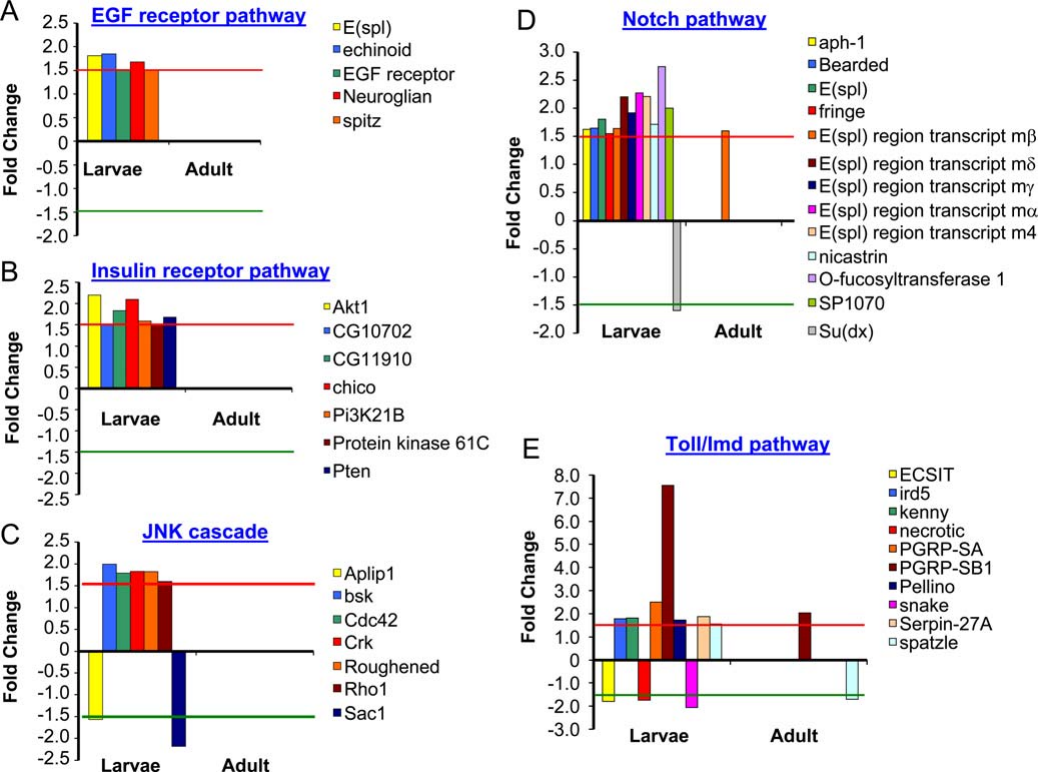
Summary of alterations in signal transduction pathways. The expression levels of genes encoding five signal transduction pathways were significantly altered in hypoxia-selected *Drosophila melanogaster* (p<0.05). The majority of the changes was an up-regulation. Significantly altered genes encoding the components A) of EGF receptor pathway, B) of the Insulin receptor pathway, C) of the JNK cascade, D) of the Notch pathway, and E) of the Toll/Imd pathway.

The significantly altered genes were further analyzed, based upon the Gene Ontological categorizations (GO) [20], by GenMAPP and MAPPFinder [20],[21]. Less than half of the differentially expressed genes (∼40%) encode for proteins whose functions have not been characterized; the remaining encode proteins that are involved in numerous biological functions, such as development, metabolism, defense mechanisms and signal transduction (Tables S3 and S4). More than 30 biological processes were found to be altered in both larvae and adult and these were mostly related to either defense (especially immune responses, p<0.05) or metabolism (especially carbohydrate and peptide metabolism, p<0.05) (Figure 2B). The most affected processes in larvae were related to metabolism (1044 genes, especially carbohydrate metabolism, 135 genes, p<0.01). In addition, multiple components of signal transduction pathways were identified to be significantly altered in larvae and these included EGF, insulin, Notch, and Toll/Imd signaling pathways (Figure 3, p<0.05). To confirm the changes obtained from microarrays, 10 differentially expressed genes were randomly chosen and their expression levels were determined by real-time qRT-PCR using specific primers in larval samples (Table S5). The microarray results and the real-time PCR gave similar trends (r = 0.85, Figure S1).

### Changes in Genes Encoding Cellular Respiratory Enzymes

**Figure 4:**
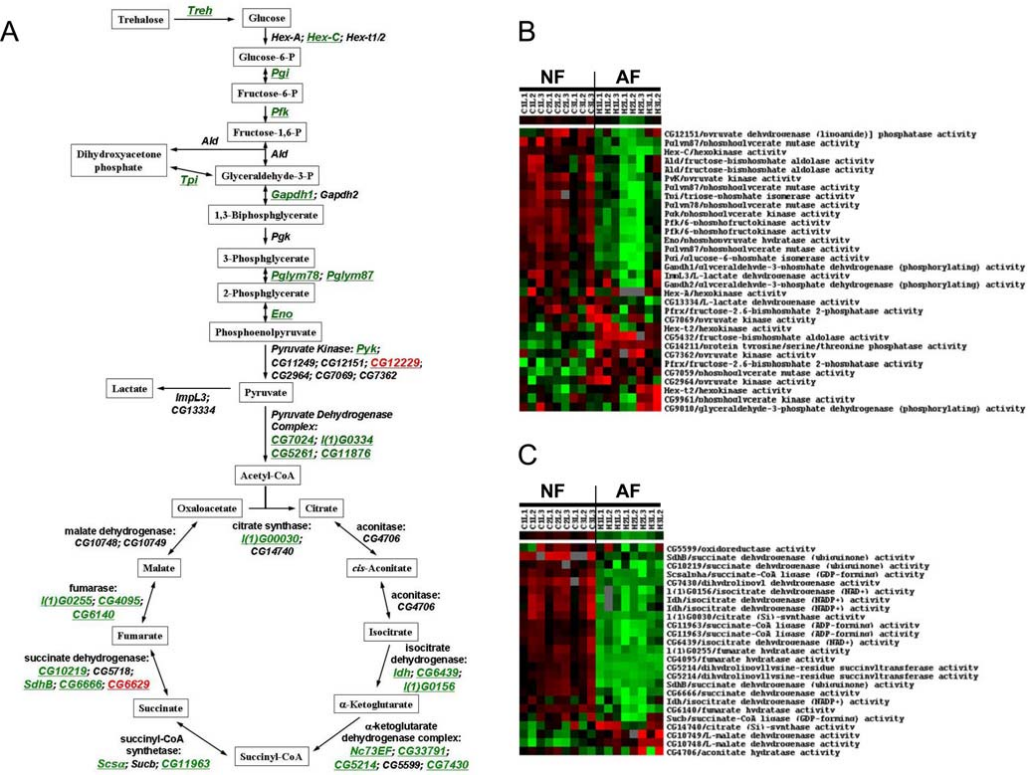
Suppression of genes encoding glycolytic and TCA cycle enzymes. A) Schematic illustration of the alterations in genes encoding glycolytic and TCA cycle enzymes (green: down-regulation, black: not significantly changed, red: up-regulation). B) Cluster map of changes in genes encoding glycolytic enzymes in NF and AF larvae. C) Cluster map of changes in genes encoding TCA cycle enzymes in NF and AF larvae. Each cluster contained 9 hybridizations of NF and 8 hybridizations of AF samples.

**Figure 5:**
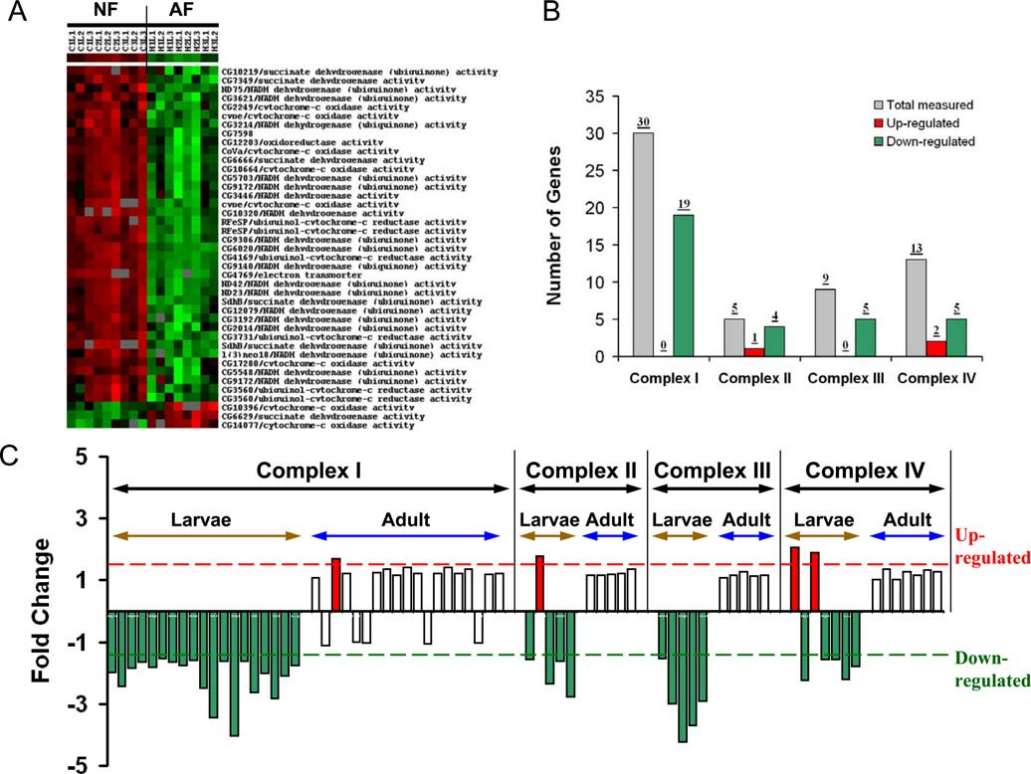
Suppression of genes encoding components of the respiratory chain complexes. A) Cluster map of changes in gene expression encoding components of respiratory chain complexes in NF and AF larvae. Each cluster contained 9 hybridizations of NF and 8 hybridizations of AF samples. B) Summary of alterations in each respiratory chain complex measured in larvae. C) Comparison of gene expression changes in each respiratory chain complex between larvae and adult.

Significant gene changes were identified in the family of genes regulating cellular respiration in AF, especially in larvae. The majority of these changes consisted of a down- rather than an up-regulation of gene expression (Table S3). Besides one pyruvate kinase isoform (*CG12229*), most of the genes encoding glycolytic enzymes were dramatically down-regulated (Figures 4A and 4B). Similarly, suppression was also found in the TCA cycle (Figures 4A and 4C), lipid β-oxidation, and respiratory chain complex genes in larvae (Figure 5). As shown in Figures 5B and 5C, among the 50 measured genes encoding components of the respiratory chain, 33 genes were down-regulated, and only 3 genes were up-regulated. Interestingly, the suppression of these metabolic genes occurred only in the larvae and not in the adult fly (Figure 5C).

### Coordinated Suppression of TCA Cycle Enzymes

**Figure 6:**
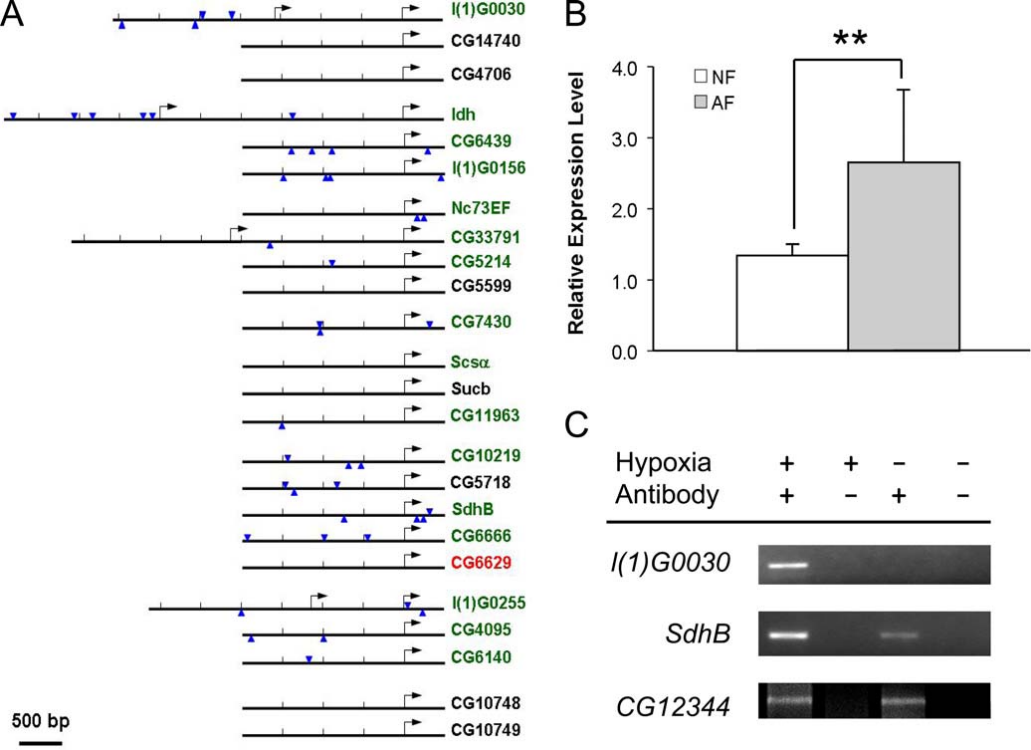
Effect of the transcriptional suppressor, *hairy*, on TCA cycle gene expression and hypoxia tolerance. A) Genomic localization of hairy binding elements in the *cis*-regulatory region of genes encoding TCA cycle enzymes. Arrowheads indicate consensus hairy binding sites and arrows indicate transcriptional start sites. B) Significant up-regulation of hairy expression in hypoxia-selected AF populations (p<0.01). C) Chromatin immunoprecipitation assay of gene *l(1)G0030*, *SdhB* and *CG12344* under normoxia or hypoxia.

Since many genes encoding metabolic enzymes were significantly down-regulated, we asked whether such down-regulation was coordinated at a transcriptional level. Therefore, the GenomatixSuite (GEMS) software was used to identify transcription factor binding elements in defined *cis*-regulatory regions of the TCA cycle, glycolysis and lipid β-oxidation genes. The TCA cycle related genes were separated into two groups, one containing the significantly down-regulated genes (down-regulated group, 16 genes) and the other containing those that were not significantly altered or up-regulated genes (reference group, 8 genes) (Table S6). The binding elements of the *Drosophila* transcriptional suppressor *hairy* were present in the regulatory region of the down-regulated genes (15 of 16 *cis*-regulatory regions, 0.88/kb) but not in the reference group (1 out of 7 *cis*-regulatory regions, 0.15/kb) (p<0.0001, CHI-Test) (Figure 6A). Of particular interest, the expression level of *hairy* was significantly up-regulated in AF (Figure 6B, Table S1). This result suggested that *hairy*, a key transcriptional suppressor, reduced the expression of the TCA cycle genes. No such specific transcription factor binding elements were found in genes encoding glycolysis or β-oxidation enzymes.

**Figure 7:**
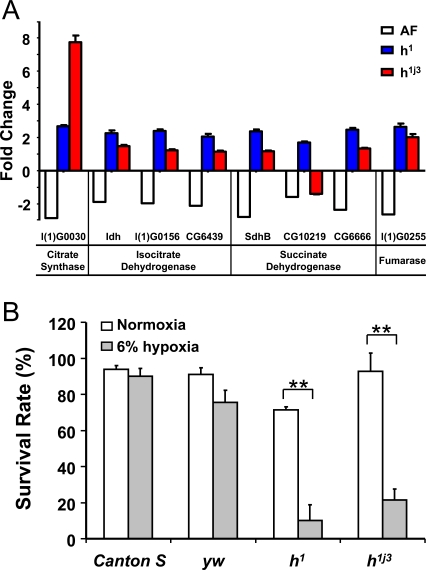
Loss-of-function *hairy* mutants abolished suppression on the expression of genes encoding TCA cycle enzymes and reduced hypoxia-tolerance in *D. melanogaster*. A) Abolished suppression of genes encoding TCA cycle enzymes in *hairy* loss-of-function mutants. The differences in gene expression were measured in *yw* control and *hairy* mutants using real-time PCR. Individual genes encoding TCA cycle enzymes had a significantly higher expression level in all *hairy* mutants than those in control except gene *CG10219*. Data were presented as means±SD (p<0.01). Gene description: l(1)G0030: citrate synthase; Idh: isocitrate dehydrogenase; l(1)G0156: isocitrate dehydrogenase; CG6439: isocitrate dehydrogenase; SdhB: succinate dehydrogenase; CG10219: succinate dehydrogenase; CG6666: succinate dehydrogenase; l(1)G0255: fumarase. B) Significant reduction in hypoxia survival in *hairy* loss-of-function mutants. Embryos from loss-of-function hairy mutants (*h^1^* and *h^1j3^*) and controls (*Canton-S* and *yw*) were cultured at normoxic or 6% O_2_ hypoxic condition. The ratio of adult flies to pupae was determined as survival rate of each stock. The loss-of-function *hairy* mutants exhibited a significantly reduced rate of hypoxia survival. Data were presented as means±SD (** p<0.01).

To confirm that *hairy* directly binds to the *cis*-regulatory regions of these candidate TCA cycle genes, chromatin immunoprecipitation (ChIP) assay was performed using a specific antibody to *hairy* in *Drosophila* Kc cells. The candidate *hairy* binding targets were tested using specific primers in both hypoxia and normoxia (Table S5). For a negative control, we included a *cis*-regulatory region of another TCA cycle gene (i.e., *CG6629*) that was up-regulated in AF and had no *hairy* binding elements detected. We found that *hairy* did bind to the *cis*-regulatory region of gene *l(1)G0030* in hypoxia but not in normoxia. Similarly, *hairy* was also found to bind to the *cis*-regulatory region of gene *SdhB* under both hypoxic and normoxic conditions, and its binding activity was significantly higher in hypoxia than in normoxia. Such hypoxia-induced increase in *hairy* binding did not occur in CG12344, a *hairy* target gene, which encodes for a non-metabolic gene (i.e., an isoform of GABA-A receptor). This result demonstrates that the down-regulated TCA cycle genes are direct targets of *hairy*, and *hairy* specifically suppresses their expression under hypoxia. Furthermore, such hypoxia-induced suppression of TCA cycle genes was abolished in the *hairy* loss-of-function mutants, *h^1^* or *h^1j3^* (Figure 7A). To further evaluate the role of *hairy* in hypoxia tolerance, we determined the survival rate of these two *hairy* loss-of-function mutants at 6% of O_2_. This mild level of O_2_ was used since it is sufficient to show differences between the mutants and controls. As shown in Figure 7B, both *hairy* loss-of-function mutants exhibited much lower survival rate (p<0.0001, CHI-Test) as compared to controls, proving the contribution of *hairy* to hypoxia tolerance in flies.

### Differences in DNA Sequence between AF and NF

**Figure 8:**
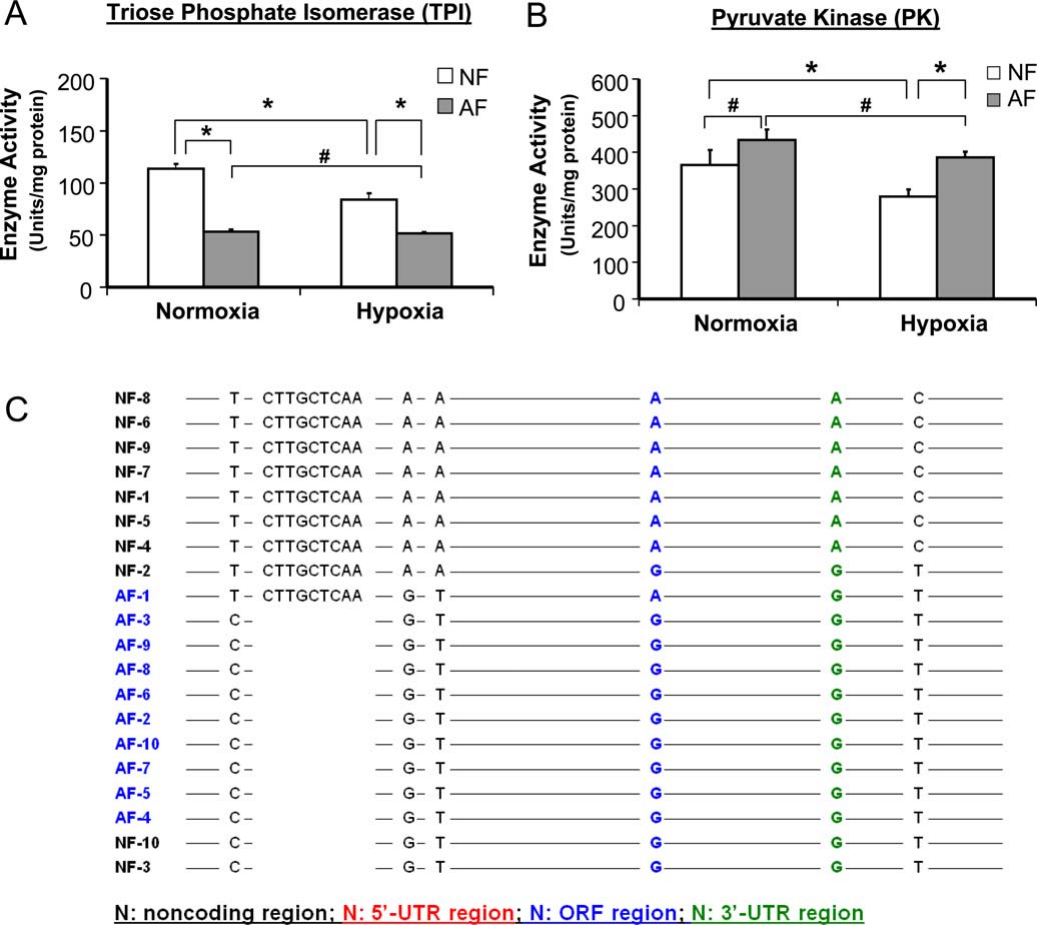
Differences in enzymic activities of triose phosphate isomerase and pyruvate kinase and allelic frequency between AF and NF. A) Enzymic activity and hypoxia response of TPI in AF and NF. TPI activity was measured in 3rd instar larvae of AF and NF cultured under either normoxia or hypoxia. AF had a significantly reduced enzymic activity than NF under both hypoxic and normoxic conditions. In contrast to the effect of hypoxia in NF, hypoxia did not induce a significant reduction on enzyme activity in AF. Data were presented as means±SD (* p<0.05, #p>0.05). B) Enzymic activity and hypoxia response of PK in AF and NF. The enzymic activity of PK was measured in 3^rd^ instar larvae of NF and AF cultured under either normoxia or hypoxia. There was no significant difference in PK activity between AF and NF cultured under normoxia. Hypoxia induced a significant reduction of PK activity in NF but not in AF. Data were presented as means±SD (* p<0.05, #p>0.05). C) Comparison of allelic frequency in the genomic region of TPI gene between AF and NF. The genomic locus encoding TPI gene was amplified by high fidelity PCR using specific primers. The PCR products were purified and cloned into pCR4-TOPO plasmid to create a plasmid library for the TPI alleles of AF and NF populations. Ten clones from the AF or NF TPI allele library were sequenced and compared by using ClustalW2 software and DnaSP 4.0. The statistical power for the comparison was calculated by GPower software 3.0.3. Seven significant polymorphic differences were identified in the AF population that included 3 SNPs (−77T/C, −53A/G, −51A/T) and 1 indel (−74 to −66) in the *cis*-regulatory region, 1 synonymous SNP (1051A/G) in the coding region, 1 SNP (1480A/G) in the 3′-untranslated region, and 1 SNP (1662C/T) in the downstream region (p<0.001, CHI-Test).

Since some of the current experiments suggested that hypoxia tolerance was a heritable trait in the AF population, we studied these flies further to determine the genetic basis of this heritability. Two types of experiments were performed. First, we examined the activity levels of 2 key glycolytic enzymes, triose phosphate isomerase (TPI) and pyruvate kinase (PK) in NF and AF. We argued that, if there was a genetic basis for the change in enzyme activity, such activity level would be altered not only in the hypoxia-cultured flies but also in those cultured in normoxia, i.e., the enzyme activity would be different from that in NF, whether in hypoxia or normoxia. As shown in Figures 8A and 8B, the activity of TPI in AF was indeed reduced when these flies were cultured in either normoxia or hypoxia. When PK activity was assessed, however, AF had a higher level activity under hypoxia and stayed at the same level in normoxia without a significant increase as compared to NF. Second, the genomic locus encoding *TPI* was sequenced to determine whether there were any differences between AF and NF. We chose to sequence the *TPI* locus, because, unlike PK, TPI is encoded by a single gene. As expected, a number of significant polymorphic differences were identified in the AF population that included 3 SNPs (−77T/C, −53A/G, −51A/T) and 1 indel (−74 to −66) in the *cis*-regulatory region, 1 synonymous SNP (1051A/G) in the coding region, 1 SNP (1480A/G) in the 3′-untranslated region, and 1 SNP (1662C/T) in the downstream region (Figure 8C, Table S7), demonstrating significant genetic differences in the AF population (p<0.001, CHI-Test).

## Discussion

The current laboratory selection experiments have shown that the hypoxia-selected flies have garnered, with ongoing generations, a spectacular ability to survive extremely low O_2_ conditions. To put this ability in perspective, these flies live perpetually now at an oxygen level that is equivalent to that above Mount Everest by ∼4,000 meters. This phenotypic breakthrough occurred after 32 generations of selection. One of the most profound phenotypic abnormalities is a reduction in body size in the AF flies. This substantial decrease in body size was presumably of physiologic relevance, which is likely related to shorten the O_2_ diffusion distances for improved survival. It is very interesting to note that, though we show evidence of heritability of the hypoxia survival trait in the selected flies, the change of body size and weight did not seem to be heritable, as body size was reverted to normal, even after a single generation in room air.

The goal of the current study was to determine the genetic and molecular basis of adaptation to *long-term* (i.e., over generations) hypoxic environments. Since survival under long term hypoxia is a complex trait, we expected this adaptation to be controlled by a number of pathways and genes. Indeed, our microarray and genetic analyses have shown that the survival of both larvae and adult flies was accompanied by alterations in a number of major signaling pathways, such as EGF, Insulin, Notch and Toll/Imd pathways. In this regard, there are a number of interesting observations made from our studies. First, the number of significantly altered genes was much larger in larvae than in the adults. More than 20% of all measured genes were significantly altered in the larval samples whereas only ∼1% changed in the adults, using the same statistical criteria. This major difference between larvae and adults may have a number of explanations, including the fact that larval cells undergo rapid growth, proliferation and differentiation as compared to adults. Second, although adaptive changes were found in both larvae and adult flies, it is possible that these changes were induced through both genetic and/or epigenetic physiologic regulation. Since we found that a) the hypoxia-selected flies could live for a number of generations in normoxia and survive again when introduced back in 4% hypoxia, b) some glycolytic enzymes maintained their activity in the AF population at a lower (or higher) level not only during hypoxia but also in normoxia (e.g., TPI and PK), and c) a number of polymorphic changes existed in AF as compared to NF, our data provided direct evidence for actual *genetic* alterations that were responsible for, at least in part, tolerance to severe hypoxia. Third, several signal transduction pathways were altered in the selected flies, i.e., EGF, Insulin, Notch and Toll/Imd pathway, which have also been found to be activated by hypoxic stimuli in mammalian tissues/cells [22]–[28]. For example, we and others have shown that the Notch receptor and its targets were altered in mouse heart [29] and cultured cells [30],[31] following chronic hypoxia. Such similarities in hypoxia responses demonstrated that some of the mechanisms underlying hypoxia-tolerance are conserved across species. Fourth, previous studies have demonstrated an enhancement of glycolytic activity as a major metabolic response in cells/tissues following *acute* (in minutes or hours) hypoxia, in contrast, our present data showed that many genes encoding glycolytic enzymes were down-regulated in the AF. Moreover, no significant lactate accumulation was found in the AF flies (unpublished observations from our laboratory). Finally, although hypoxia-tolerance is a complex trait, a single gene alteration, such as that of *hairy*, can remarkably make a significant difference in terms of survival.

For more than three decades, physiologists have debated the importance of down-regulation of metabolism in hypoxia tolerance in some tolerant animals such as the turtle. For example, Hochachka and others have argued that anoxia-tolerant organisms depress their metabolism in order to minimize the mismatch between supply and demand [32]–[34]. While this idea, based on metabolic data, is intuitively appealing, there is no information about how various metabolic enzymes could be coordinated in order to survive severe long lasting hypoxia. Our current work provides the first evidence showing that such coordination can be achieved at a transcriptional level by a seemingly *metabolic switch*, i.e., *hairy*, which is a transcriptional suppressor. This notion is supported by a number of observations from current study: a) expression of *hairy* was up-regulated in AF, b) *hairy*-binding region was presented in the *cis*-regulatory regions of the down-regulated genes but not others, c) the functional ChIP analysis led us to believe that the down-regulation of these metabolic genes is based on the changes of *hairy*, *especially* that its binding was not increased for other, non-metabolic target genes, e.g., CG12344, and d) *hairy* loss-of-function mutations abolished the suppression on the expression of TCA cycle genes and significantly reduced hypoxia survival in *Drosophila*. This decreased survival in flies carrying the *hairy* mutation is particularly important since these experiments strongly demonstrate that *hairy* contributes to hypoxia tolerance through the regulation of TCA cycle enzymes. While it is possible that the decreased survival is not related to the role that *hairy* plays in metabolic regulation but due to an inherent weakness of the *hairy* mutants, this is much less likely because a) we have studied in the past different mutation alleles in flies and they do not necessarily behave differently in hypoxia [6], and b) we chose two different mutants of *hairy* and they have similar effects.

Another interesting observation in this work is related to the enzymatic activities of triose-phosphate isomerase (TPI) and pyruvate kinase (PK) in AF. We hypothesized that, if there is a genetic basis for the change in enzyme activity, we should be able to detect the alterations in AF flies that were cultured in both hypoxia and normoxia, and the enzyme activity should maintain different from those in NF, whether in hypoxia or normoxia. Indeed, as expected, we found that the expression level and enzymatic activity of TPI were significantly lower in AF than those in NF in both hypoxia and normoxia. In addition, we identified 7 polymorphic differences in the genomic locus of the TPI gene in AF which demonstrated a genetic modification of this locus in AF strain. In contrast, the activity of PK in AF *did not* differ from that of NF in normoxia, although its activity was significantly higher in AF under hypoxia (Figure 8B). This result demonstrated that there was an inhibition of this enzyme in NF under hypoxia, and such hypoxia-induced inhibition was abolished in AF. On the surface, this result might weaken our argument, a potential explanation for such a difference between PK and TPI is that the regulation of PK is much more complicated. Indeed, there are 7 genes encoding PK in *D. melanogaster*. In AF, we found that 1 gene (i.e., *Pyk*) was down-regulated and another gene (i.e., *CG12229*) was up-regulated. Therefore, in addition to possible genetic rearrangements, other factors may play a role to keep higher activity of PK in AF under hypoxia and to minimize the difference between AF and NF in normoxia.

If the changes that led to enhanced survival in extremely low O_2_ levels in the AF population are genetic in nature, as we demonstrated in this work, it is important to note that the breakthroughs in survival to lower and lower O_2_ levels took place after a relatively small number of generations in the *Drosophila*. This relatively small number of generations was also found when previous investigators were studying other phenomena such as geotaxis [15]. If translated into human years, the 32 generations that were needed to alter the phenotype and genotype in *Drosophila* can be approximated to about a thousand years. Although changes in the DNA code with selection pressure could take a much longer period of time (potentially thousands and millions of years in “Darwinian time”), this work demonstrated that such changes in DNA and phenotype could proceed at a much faster rate, presumably because of the trait itself or the selection pressure applied.

In summary, we have generated a *Drosophila melanogaster* strain that is very tolerant to severe hypoxic conditions through long-term experimental selection. Several adaptive changes have been identified in the hypoxia-selected AF flies that include up-regulation of multiple signal transduction pathways, modulation of cellular respiration enzymes, and polymorphic differences in metabolic enzymes such as TPI. While we believe that multiple pathways contribute to hypoxia-tolerant trait in this *Drosophila* strain, we demonstrate that *hairy*-mediated metabolic suppression is an important one. The adaptive mechanisms identified in this hypoxia-tolerant *Drosophila* model may also play a crucial role in protecting mammals from hypoxia injury.

## Materials and Methods

### *Drosophila melanogaster* Stocks, Hypoxia Selection, and Phenotypic Assays

Twenty-seven isogenic *Drosophila melanogaster* lines (kindly provided by Dr. Andrew Davis) were used as parental stocks for the long-term hypoxia-selection experiment as described previously [17]. Briefly, embryos collected from the parental population were divided into 6 groups, 3 groups of them were subjected to long-term hypoxia-selection, and 3 other groups were maintained under normoxia as controls. Both hypoxia-selection and control experiments were performed in specially designed population chambers (26 cm×16 cm×16 cm). These chambers were connected to either O_2_ at certain concentrations (balanced with N_2_, for the hypoxia-selection experiments) or to room air (21% O_2_, for the control experiments). The humidity in the chambers was maintained by passing the gas through water prior to going into the chambers. The flow speed was monitored by 565 Glass Tube Flowmeter (Concoa, Virginia Beach, VA), and the O_2_ level within the chamber was monitored with Diamond General 733 Clark Style Electrode (Diamond General Development Corp., Ann Arbor, MI). The selection was started at 8% O_2_ and this concentration was gradually decreased by 1% each 3 to 5 generations to keep the selection pressure. Embryos, 3^rd^ instar larvae and adult flies were collected from each generation and stored at −80°C for analyses. The results presented in the current study were derived from expression arrays using larval and adult samples.

The body weight of hypoxia-selected flies was determined at each generation. Male flies (n = 100) from hypoxia or control chambers were collected, weighed and used as the index of body weight.

The *hairy* loss-of-function mutants (*h^1^* and *h^1j3^*) were obtained from *Drosophila* Stock Center (Bloomington, IN). The survival rate of these stocks in hypoxia was determined by culturing them in a 6% O_2_ environment. After 3 weeks in culture, the number of live adult flies and pupae were counted. The ratio between live adult flies to the total number of pupae was calculated and presented as survival rate. The statistical significance of survival between *hairy* mutants and controls was calculated by CHI-test.

### cDNA Microarray Analysis

cDNA microarrays containing 13,061 known or predicted genes of the *D. melanogaster* genome were processed according to previous descriptions [16],[35]. Nine larval samples from AF or NF, 6 adult samples from AF or NF were included in this analysis. Each larval sample contained a pool of 25 3^rd^ instar larvae, and each adult sample contained 25 male and 25 female adult flies from each individual population. Total RNA was extracted from the samples using TRIzol (Invitrogen, Carlsbad, CA) followed by a clean-up with RNeasy kit (Qiagen, Valencia, CA). Three µg of total RNA from each sample was amplified with an *in vitro* transcription-based strategy [13]. A common reference design was applied for the hybridizations, and the reference RNA sample was created using a balanced pool of 3^rd^ instar larvae (for the larval samples) or adult flies (for the adult samples) from each parental line. This reference was chosen so that relative abundance of each transcript could be calculated individually, and the relative levels of each transcript among biological replicates could be compared. A total of 30 arrays were included in this analysis, and the hybridizations were done in different days using arrays printed from different batches. Microarray images were acquired by GenePix 4000 microarray scanner using GenePix Pro 3 microarray analysis software (Axon Instruments, Sunnyvale, CA, USA). The statistical significance (q-value, i.e., false discover rate (FDR)) and the ratio of the changes in expression was calculated using Significance Analysis of Microarray (SAM) software [19] following LOWESS normalization. The fold changes were presented as ratios, if up-regulated, or −1/ratio, if down-regulated. The gene ontology (GO) based analyses were performed using GenMAPP software [20]. The microarray data can be retrieved using access number GSE8803 in the GEO database at http://www.ncbi.nlm.nih.gov/geo.

### Semi-Quantitative Real-Time RT-PCR

Semi-quantitative real-time RT-PCR was used to evaluate the result of microarrays and to determine the differences in expression levels of genes encoding TCA cycle enzymes between the *hairy* mutants and control. All specific primers were designed by Primer 3 software [36] and synthesized at ValueGene (San Diego, CA) (Table S5). First strand cDNA was synthesized using SuperScriptII reverse transcriptase and Oligo-(dT) primer. Real-time PCR amplification was performed using ABI Prism 7900HT Sequence Detection System (Applied Biosystems, Foster City, CA). For each reaction, 10 µl of 2× SYBR green PCR master mix (Applied Biosystems, Foster City, CA) and 0.5 µM of both forward and reverse primers along with 100 ng of each appropriate cDNA samples were mixed (total reaction volume: 20 µl). Melting curves were determined and the final products were isolated with 4% agarose gel to ensure specificity of the reaction. The relative expression level was calculated using 2^−ΔΔ*C*t^ method, as described previously [37]–[39]. *Drosophila melanogaster* β-actin was used as internal control. The final results were presented as fold change of AF over NF or *hairy* mutants over *yw* control. All experiments were done in triplicate.

### Enzyme Activity Assays

The enzymatic activity of Triose Phosphate Isomerase (TPI) and Pyruvate Kinase (PK) were determined as previously described with modifications [40]. Briefly, the assay samples were extracted from 3rd instar larvae cultured under either normoxic or hypoxic condition (4% O_2_). 0.2 ml (100 mg wet weight/ml) of isolation medium (0.25 M sucrose,1 mM EDTA-K, 5 mM HEPES-Tris, pH 7.4, with protease inhibitors) was added and the suspension of larval tissue in isolation medium was transferred into a 2 ml all glass homogenizer. 10% (v/v) Triton X-100 was added to this suspension making the final concentration of Triton X-100 0.5% (v/v). Then the tissue was homogenized with the B (i.e., tight) pestle. Aliquots of this homogenate were employed for enzymatic activity measurements. The enzymatic activities of TPI or PK were determined using 10, 20, or 30 µl of the homogenate with proper substrates. The dynamic of color formation was recorded using Beckman Coulter DU800 Spectrophotometer at selected wave length for each substrate. The kinetic parameters of the reaction were calculated by curve fitting.

### Identification of Transcription Factor Binding Element in *cis*-Regulatory Region

The common transcription factor binding sites in the defined promoter regions of candidate genes were analyzed using GenomatixSuite (Genomatix Software GmbH, Germany). The genes encoding TCA cycle or β-oxidation enzymes were separated into down-regulated or reference groups. The genome DNA sequences from 2000 bp upstream of the first transcription starting site (TSS) to 500 bp downstream of the last TSS of each gene was downloaded and used as *cis*-regulatory region of the gene (*Drosophila* genome R5.2) [41]. These sequences were subjected to GEMS analysis to identify common transcription factor binding elements [42]. The statistical significance of the transcription factor binding element frequency in the AF and NF population was analyzed by CHI-test.

### ChIP Assay

Chromatin immunoprecipitation and PCR detection of hairy binding in the *cis*-regulatory regions of genes encoding TCA cycle enzymes were performed in cultured *Drosophila* Kc cells (obtained from Dr. Amy Kiger, UCSD) [43],[44]. The Kc cells were treated with 0.5% O_2_ for 4 hours. About 10^6^ cells were used in each ChIP experiment. Chromatin immunoprecipitation was carried out using ChIP assay kit (Upstate, Temecula, CA) according to the manufacture's instructions. The immunoprecipitation was performed overnight at 4°C with 2 µg of hairy antibody (Abcam, Cambridge, MA). DNA fragments were purified with phenol:chloroform (Invitrogen, Carlsbad, CA). For PCR, 2 µl of a 25 µl DNA extraction was amplified with specific primers (Table S5).

### DNA Sequencing and Analysis

Genomic DNA was extracted from 15 male AF or NF adult flies. The samples were ground in 400 µl of homogenate buffer (100 mM Tris/HCl, 100 mM EDTA, 100 mM NaCl and 0.5% SDS, pH7.5) and incubated at 65°C for 30 min. Genomic DNA was extracted by adding in 800 µl extraction solution (1 2.5 (v/v) of 5 M KAc and 6 M LiCl). After 15 min of centrifugation at 13,000 rpm, the supernatant was transferred into a new tube and the genomic DNA was precipitated by adding 600 µl of isopropanol. The DNA pellet was washed with 70% of ethanol and dissolved in TE buffer.

The genomic locus encoding TPI gene was amplified by PCR using specific primers (forward primer: GTTTAAGGTCCGCAGAGGTG, and reverse primer: ATTTTGGCAAGCCTGTTGAT). All the coding exons and intronic flanking regions were amplified by polymerase chain reaction (PCR) using the high fidelity proofreading DNA polymerase, Platinum *Pfx* DNA Polymerase (Invitrogen, Carlsbad, CA). The PCR products were purified and cloned into pCR4-TOPO plasmid to create the plasmid library for TPI alleles of the AF and NF population. Cycle sequencing was performed on an ABI automated sequencer (Applied Biosystems, Foster City, CA) by Eton Biosciences, Inc. (San Diego, CA). Ten clones from AF or NF TPI allele library were sequenced and compared by using ClustalW2 software [45] (http://www.ebi.ac.uk/Tools/clustalw2/) and DnaSP 4.0 [46]. The statistical power for the comparison was calculated by GPower software 3.0.3 [47].

## Supporting Information

Figure S1 Correlation between Microarray and qRT-PCR Results.
(0.83 MB TIF)

Table S1 List of Significantly Altered Genes in Hypoxia-Selected *Drosophila melanogaster* at Larval Stage.
(0.23 MB PDF)

Table S2 List of Significantly Altered Genes in Hypoxia-Selected *Drosophila melanogaster* at Adult Stage.
(0.08 MB PDF)

Table S3 List of Significantly Altered Biological Processes in Hypoxia-Selected *Drosophila melanogaster* at Larval Stage.
(0.18 MB PDF)

Table S4 List of Significantly Altered Biological Processes in Hypoxia-Selected *Drosophila melanogaster* at Adult Stage.
(0.11 MB PDF)

Table S5 Primers for qRT-PCR and ChIP-PCR Assays.
(0.02 MB PDF)

Table S6 Summary of Genomic Locations of *hairy* Transcription Repressor Binding Elements in the *cis*-Regulatory Regions of Genes Encoding TCA Cycle Enzymes.
(0.03 MB PDF)

Table S7 Comparison of the TPI Genomic Locus between NF and AF with Multiple Sequence Alignment.
(0.06 MB PDF)
