# Supplementary figures and images for: Mechanisms Underlying Hypoxia Tolerance in Drosophila melanogaster: hairy as a Metabolic Switch
Source: PLoS Genet. 2008 Oct 17;4(10):e1000221. doi: 10.1371/journal.pgen.1000221 (PMC2556400; doi:10.1371/journal.pgen.1000221)

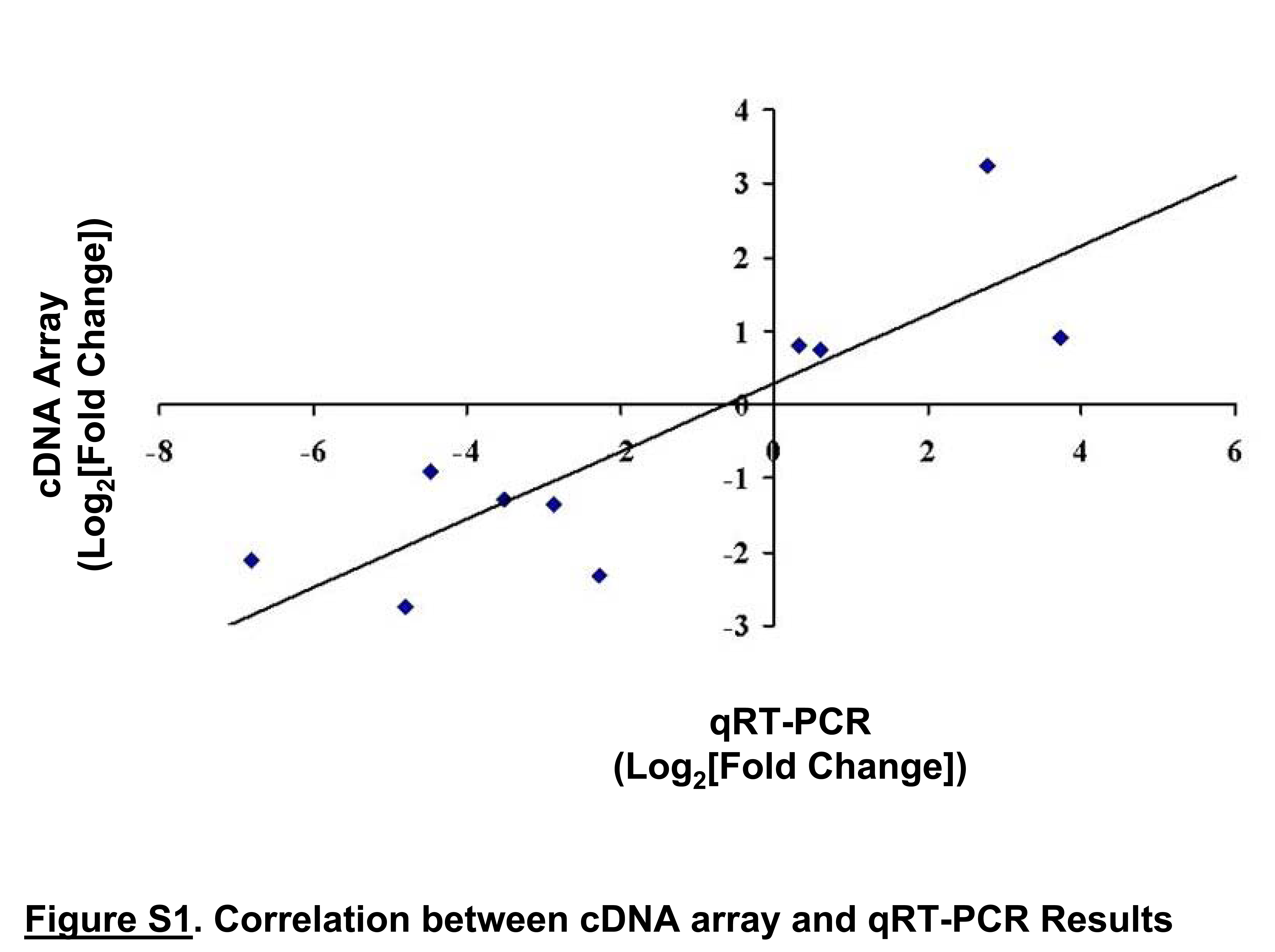

Supplement: Figure S1 — Correlation between Microarray and qRT-PCR Results. (0.83 MB TIF) [file pgen.1000221.s001.tif]
