# Supplementary material for: Mechanisms Underlying Hypoxia Tolerance in Drosophila melanogaster: hairy as a Metabolic Switch
Source: PLoS Genet. 2008 Oct 17;4(10):e1000221. doi: 10.1371/journal.pgen.1000221 (PMC2556400; doi:10.1371/journal.pgen.1000221)
Supplement: Table S1 — List of Significantly Altered Genes in Hypoxia-Selected Drosophila melanogaster at Larval Stage. (0.23 MB PDF) [file pgen.1000221.s002.pdf]

Table S1. List of Significantly Altered Genes in Hypoxia-Selected *Drosophila melanogaster* at Larval Stage

Flybase ID	Symbol	Full_name	Fold Change	q-value(%)
FBgn0035868	CG7194	CG7194	9.93	0.00
FBgn0040559	CG14359	CG14359	9.45	0.00
FBgn0012042	AttA	Attacin-A	8.98	0.00
FBgn0041581	AttB	Attacin-B	8.78	0.00
FBgn0030357	CG2471	CG2471	8.03	0.00
FBgn0038509	CG14332	CG14332	7.92	0.00
FBgn0040992	CG10570	CG10570	7.83	0.00
FBgn0039685	Obp99b	Odorant-binding protein 99b	7.63	0.00
FBgn0043578	PGRP-SB1	PGRP-SB1	7.56	0.00
FBgn0032280	CG17105	CG17105	7.40	0.00
FBgn0041579	AttC	Attacin-C	6.58	0.00
FBgn0003372	Sgs1	Salivary gland secretion 1	6.47	0.00
FBgn0039299	CG11854	CG11854	6.10	0.00
FBgn0000594	Est-P	Esterase P	5.73	0.00
FBgn0030544	CG13403	CG13403	5.63	0.00
FBgn0045761	CG10618	CG10618	5.61	0.00
FBgn0033920	CG8561	CG8561	5.20	0.00
FBgn0037346	eIF-5C	eukaryotic initiation factor 5C	5.06	0.00
FBgn0035813	CG8492	CG8492	5.01	0.00
FBgn0032281	CG17107	CG17107	5.00	0.00
FBgn0000640	Fbp2	Fat body protein 2	4.93	0.00
FBgn0035089	Phk-3	Pherokine 3	4.87	0.00
FBgn0050029	CG30029	CG30029	4.76	0.00
FBgn0040565	CG7606	CG7606	4.73	0.00
FBgn0034428	CG18606	CG18606	4.65	0.00
FBgn0033857	CG13335	CG13335	4.65	0.00
FBgn0030443	CG12715	CG12715	4.63	0.00
FBgn0033137	Tsp42Ep	Tetraspanin 42Ep	4.54	0.00
FBgn0001224	Hsp23	Heat shock protein 23	4.51	0.00
FBgn0037370	CG1236	CG1236	4.51	0.00
FBgn0038943	CG5391	CG5391	4.35	0.00
FBgn0034407	DptB	Diptericin B	4.33	0.00
FBgn0002533	Lcp2	Larval cuticle protein 2	4.28	0.00

FBgn0041604	dlp	dally-like	4.27	0.00
FBgn0034426	CG10476	CG10476	4.24	0.00
FBgn0030105	CG15369	CG15369	4.23	0.00
FBgn0026190	PH4alphaMP	prolyl-4-hydroxylase-alpha MP	4.20	0.00
FBgn0003378	Sgs8	Salivary gland secretion 8	4.18	0.00
FBgn0002562	Lsp1alpha	Larval serum protein 1 alpha	4.17	0.00
FBgn0004577	Pxd	Peroxidase	4.15	0.00
FBgn0038523	CG7587	CG7587	4.13	0.00
FBgn0031327	CG5397	CG5397	4.11	0.00
FBgn0036410	CG8100	CG8100	4.07	0.00
FBgn0025456	CREG	Cellular Repressor of E1A-stimulated Genes	4.04	0.00
FBgn0039482	CG14258	CG14258	4.01	0.00
FBgn0036030	CG6767	CG6767	4.01	0.00
FBgn0032286	CG7300	CG7300	3.87	0.00
FBgn0033725	CG8502	CG8502	3.79	0.00
FBgn0037372	CG2091	CG2091	3.78	0.00
FBgn0040503	CG7763	CG7763	3.78	0.00
FBgn0000173	ben	bendless	3.77	0.00
FBgn0028373	inx3	innexin 3	3.76	0.00
FBgn0003377	Sgs7	Salivary gland secretion 7	3.75	0.00
FBgn0015396	jumu	jumeau	3.75	0.00
FBgn0034856	yellow-d2	yellow-d2	3.73	0.00
FBgn0031971	CG7224	CG7224	3.69	0.00
FBgn0038449	CG17562	CG17562	3.67	0.00
FBgn0026144	CBP	sarcoplasmic calcium-binding protein	3.59	0.00
FBgn0036587	CG4950	CG4950	3.58	0.00
FBgn0010381	Drs	Drosomycin	3.49	0.00
FBgn0001321	knk	knickkopf	3.47	0.00
FBgn0039099	CG10157	CG10157	3.47	0.00
FBgn0031248	CG11912	CG11912	3.46	0.00
FBgn0031747	CG9021	CG9021	3.44	0.00
FBgn0041194	Prat2	Phosphoribosylamidotransferase 2	3.44	0.00
FBgn0037114	CG7160	CG7160	3.44	0.00
FBgn0039030	CG6660	CG6660	3.43	0.00
FBgn0036468	CG13461	CG13461	3.42	0.00
FBgn0050480	CG30480	CG30480	3.40	0.00

FBgn0002564	Lsp1gamma	Larval serum protein 1 gamma	3.38	0.00
FBgn0000639	Fbp1	Fat body protein 1	3.31	0.00
FBgn0050440	CG30440	CG30440	3.31	0.00
FBgn0037975	CG3397	CG3397	3.30	0.00
FBgn0036393	CG17362	CG17362	3.27	0.00
FBgn0032639	CG18563	CG18563	3.27	0.00
FBgn0039593	CG9989	CG9989	3.26	0.00
FBgn0040817	CG14132	CG14132	3.26	0.00
FBgn0036782	CG7320	CG7320	3.21	0.00
FBgn0039239	CG13641	CG13641	3.20	0.00
FBgn0036929	CG7668	CG7668	3.18	0.00
FBgn0026431	Grip75	Grip75	3.17	0.00
FBgn0003375	Sgs5	Salivary gland secretion 5	3.17	0.00
FBgn0037235	CG1103	CG1103	3.16	0.00
FBgn0028956	mthl3	methuselah-like 3	3.15	0.00
FBgn0036985	CG5847	CG5847	3.14	0.00
FBgn0031893	CG4495	CG4495	3.14	0.00
FBgn0038083	CG5999	CG5999	3.14	0.00
FBgn0036198	CG6038	CG6038	3.13	0.00
FBgn0022073	Thor	Thor	3.12	0.00
FBgn0031579	CG15422	CG15422	3.09	0.00
FBgn0038919	CG17843	CG17843	3.09	0.00
FBgn0028552	gammaSnap	gamma-soluble NSF attachment protein	3.08	0.00
FBgn0037083	CG5656	CG5656	3.04	0.00
FBgn0031872	iHog	interference Hedgehog	3.03	0.00
FBgn0004879	plx	pollux	3.02	0.00
FBgn0031471	CG3117	CG3117	3.01	0.00
FBgn0004859	ci	cubitus interruptus	3.01	0.00
FBgn0040532	CG8369	CG8369	3.00	0.00
FBgn0037974	CG12224	CG12224	2.97	0.00
FBgn0032785	CG10026	CG10026	2.97	0.00
FBgn0052091	CG32091	CG32091	2.96	0.00
FBgn0037782	CG12813	CG12813	2.95	0.00
FBgn0015024	CkIalpha	Casein kinase Ialpha	2.94	0.00
FBgn0032230	CG13139	CG13139	2.93	0.00
FBgn0037814	CG6325	CG6325	2.93	0.00

FBgn0035176	CG13905	CG13905	2.93	0.00
FBgn0031559	CG3513	CG3513	2.92	0.00
FBgn0037672	sage	salivary gland-expressed bHLH	2.91	0.00
FBgn0038420	CG10311	CG10311	2.90	0.00
FBgn0028992	sds22	sds22	2.90	0.00
FBgn0032284	CG7294	CG7294	2.89	0.00
FBgn0026170	smt3	smt3	2.89	0.00
FBgn0015035	Cyp4e3	Cytochrome P450-4e3	2.87	0.00
FBgn0036467	CG12310	CG12310	2.87	0.00
FBgn0040832	CG8012	CG8012	2.87	0.00
FBgn0026076	UBL3	UBL3	2.87	0.00
FBgn0024194	rasp	rasp	2.87	0.00
FBgn0051778	CG31778	CG31778	2.86	0.00
FBgn0035959	CG4911	CG4911	2.85	0.00
FBgn0038009	CG17738	CG17738	2.85	0.00
FBgn0037007	CG5059	CG5059	2.84	0.00
FBgn0038508	CG5866	CG5866	2.84	0.00
FBgn0031220	CG4822	CG4822	2.83	0.00
FBgn0041711	yellow-e	yellow-e	2.82	0.00
FBgn0040369	CG13377	CG13377	2.82	0.00
FBgn0041182	TepII	Thiolester containing protein II	2.81	0.00
FBgn0036101	ninA	ninjurin A	2.81	0.00
FBgn0035936	Tsp66E	Tetraspanin 66E	2.80	0.00
FBgn0030326	CG2444	CG2444	2.79	0.00
FBgn0031216	CG11376	CG11376	2.79	0.00
FBgn0040833	CG7498	CG7498	2.79	0.00
FBgn0034901	CG11300	CG11300	2.78	0.00
FBgn0010228	HmgZ	HMG protein Z	2.78	0.00
FBgn0032682	CG10176	CG10176	2.76	0.00
FBgn0038037	Cyp9f2	Cyp9f2	2.74	0.00
FBgn0014865	Mtk	Metchnikowin	2.74	0.00
FBgn0052405	CG32405	CG32405	2.74	0.00
FBgn0033875	CG6357	CG6357	2.74	0.00
FBgn0033901	O-fut1	O-fucosyltransferase 1	2.74	0.00
FBgn0011260	Sema-2a	Sema-2a	2.74	0.00
FBgn0035806	PGRP-SD	PGRP-SD	2.73	0.00

FBgn0032400	CG6770	CG6770	2.73	0.00
FBgn0001257	ImpL2	Ecdysone-inducible gene L2	2.73	0.00
FBgn0037810	sle	slender lobes	2.71	0.00
FBgn0037127	CG14566	CG14566	2.71	0.00
FBgn0036556	CG5830	CG5830	2.70	0.00
FBgn0027108	inx2	innexin 2	2.70	0.00
FBgn0051103	CG31103	CG31103	2.69	0.00
FBgn0030685	Graf	Graf	2.68	0.00
FBgn0022893	Df31	Decondensation factor 31	2.68	0.00
FBgn0039241	CG11089	CG11089	2.67	0.00
FBgn0001197	His2Av	Histone H2A variant	2.67	0.00
FBgn0041094	scyl	scylla	2.67	0.00
FBgn0038074	CG6188	CG6188	2.67	0.00
FBgn0038053	CG18549	CG18549	2.67	0.00
FBgn0026620	tacc	transforming acidic coiled-coil protein	2.66	0.00
FBgn0004646	ogre	optic ganglion reduced	2.65	0.00
FBgn0013997	Nrx-IV	Neurexin IV	2.63	0.00
FBgn0031937	CG13795	CG13795	2.63	0.00
FBgn0031250	Ent1	Equilibrative nucleoside transporter 1	2.63	0.00
FBgn0052672	Atg8a	Autophagy-specific gene 8a	2.63	0.00
FBgn0002440	l(3)mbn	lethal (3) malignant blood neoplasm	2.62	0.00
FBgn0030876	CG6762	CG6762	2.62	0.00
FBgn0053047	CG11714	CG11714	2.61	0.00
FBgn0040222	fne	found in neurons	2.61	0.00
FBgn0035290	CG1887	CG1887	2.60	0.00
FBgn0035680	loj	logjam	2.60	0.00
FBgn0035571	CG12493	CG12493	2.59	0.00
FBgn0005626	ple	pale	2.59	0.00
FBgn0030734	CG9911	CG9911	2.58	0.00
FBgn0034429	CG18607	CG18607	2.57	0.01
FBgn0038366	CG4576	CG4576	2.57	0.00
FBgn0039172	CG5677	CG5677	2.57	0.00
FBgn0032669	CG15155	CG15155	2.57	0.00
FBgn0036769	Tsp74F	Tetraspanin 74F	2.55	0.00
FBgn0011327	Uch-L3	Ubiquitin C-terminal hydrolase	2.54	0.00
FBgn0031914	CG5973	CG5973	2.54	0.00

FBgn0036128	CG11801	CG11801	2.54	0.00
FBgn0035688	CG10289	CG10289	2.54	0.00
FBgn0035528	CG15012	CG15012	2.54	0.00
FBgn0035877	CG7083	CG7083	2.54	0.00
FBgn0028536	CG15281	CG15281	2.53	0.00
FBgn0022238	lolal	lola like	2.53	0.00
FBgn0036553	CG17027	CG17027	2.53	0.00
FBgn0010240	Lcch3	Ligand-gated chloride channel homolog 3	2.52	0.00
FBgn0051075	CG31075	CG31075	2.52	0.00
FBgn0015772	Nak	Numb-associated kinase	2.51	0.00
FBgn0034631	CG10496	CG10496	2.51	0.00
FBgn0036525	CG13451	CG13451	2.51	0.00
FBgn0032949	CG3305	CG3305	2.51	0.00
FBgn0030310	PGRP-SA	Peptidoglycan recognition protein SA	2.51	0.00
FBgn0031809	CG9508	CG9508	2.50	0.00
FBgn0035423	CG17737	CG17737	2.49	0.00
FBgn0034341	GstE7	Glutathione S transferase E7	2.49	0.00
FBgn0000239	bur	burgundy	2.49	0.00
FBgn0013303	Nca	Neurocalcin	2.49	0.00
FBgn0038088	CG10126	CG10126	2.48	0.00
FBgn0001226	Hsp27	Heat shock protein 27	2.48	0.00
FBgn0037583	CG9684	CG9684	2.48	0.00
FBgn0031558	CG16704	CG16704	2.48	0.00
FBgn0003969	vap	vacuolar peduncle	2.47	0.00
FBgn0000575	emc	extra macrochaetae	2.47	0.00
FBgn0004838	Hrb27C	Heterogeneous nuclear ribonucleoprotein at 27C	2.47	0.00
FBgn0038412	Zip3	Zinc/iron regulated transporter-related protein 3	2.46	0.00
FBgn0032223	GATAd	GATAd	2.46	0.00
FBgn0030494	CG15757	CG15757	2.46	0.00
FBgn0035711	CG8519	CG8519	2.45	0.00
FBgn0031873	Gas41	Gas41	2.45	0.00
FBgn0050069	CG30069	CG30069	2.45	0.00
FBgn0036264	CG11529	CG11529	2.45	0.00
FBgn0028546	Rsu-1	Ras suppressor-1	2.45	0.00
FBgn0032035	CG13393	CG13393	2.44	0.00
FBgn0037150	CG7133	CG7133	2.44	0.00

FBgn0034881	CG10332	CG10332	2.44	0.00
FBgn0003374	Sgs4	Salivary gland secretion 4	2.44	0.01
FBgn0000308	chic	chickadee	2.43	0.00
FBgn0036394	CG9040	CG9040	2.43	0.00
FBgn0028939	CG18146	CG18146	2.43	0.00
FBgn0000541	E(bx)	Enhancer of bithorax	2.43	0.00
FBgn0037728	CG16817	CG16817	2.42	0.00
FBgn0031936	CG13794	CG13794	2.42	0.00
FBgn0031470	CG18557	CG18557	2.42	0.00
FBgn0034786	CG13531	CG13531	2.41	0.00
FBgn0004889	tws	twins	2.41	0.00
FBgn0040250	Ugt86Dj	Ugt86Dj	2.41	0.00
FBgn0033942	CG10112	CG10112	2.41	0.00
FBgn0038115	CG7966	CG7966	2.39	0.00
FBgn0053006	CG18431	CG18431	2.39	0.00
FBgn0035773	bhr	bhringi	2.39	0.00
FBgn0026192	par-6	par-6	2.39	0.00
FBgn0004607	zfh2	Zn finger homeodomain 2	2.39	0.00
FBgn0026257	cav	caravaggio	2.38	0.00
FBgn0024806	DIP2	DISCO Interacting Protein 2	2.38	0.00
FBgn0035985	CG3672	CG3672	2.38	0.00
FBgn0040373	CG3038	CG3038	2.37	0.00
FBgn0003345	sd	scalloped	2.37	0.00
FBgn0026077	Gasp	Gasp	2.36	0.00
FBgn0051741	CG31741	CG31741	2.36	0.00
FBgn0003206	Ras64B	Ras oncogene at 64B	2.35	0.00
FBgn0034647	CG15678	CG15678	2.35	0.00
FBgn0029687	Vap-33-1	Vap-33-1	2.35	0.00
FBgn0035815	CG7422	CG7422	2.34	0.00
FBgn0032243	Klp31E	Klp31E	2.34	0.00
FBgn0040494	CG7242	CG7242	2.34	0.00
FBgn0027584	CG4757	CG4757	2.34	0.00
FBgn0037636	CG9821	CG9821	2.32	0.00
FBgn0036893	CG9376	CG9376	2.32	0.00
FBgn0036930	fat2	fat2	2.32	0.00
FBgn0037069	CG7658	CG7658	2.32	0.05

FBgn0028582	lqf	liquid facets	2.32	0.00
FBgn0003124	polo	polo	2.31	0.00
FBgn0028690	Rpn5	Rpn5	2.31	0.00
FBgn0030262	CG2081	CG2081	2.31	0.00
FBgn0028411	Nxt1	NTF2-related export protein 1	2.31	0.00
FBgn0030418	CG4004	CG4004	2.30	0.00
FBgn0010051	Itp-r83A	Inositol 1,4,5,-tris-phosphate receptor	2.30	0.00
FBgn0015796	Rab8	Rab-protein 8	2.30	0.00
FBgn0037376	CG2051	CG2051	2.30	0.00
FBgn0035692	CG13298	CG13298	2.29	0.00
FBgn0052238	CG32238	CG32238	2.29	0.00
FBgn0039209	CG13624	CG13624	2.29	0.00
FBgn0035049	Mmp1	Matrix metalloproteinase 1	2.29	0.00
FBgn0037357	sec23	sec23	2.28	0.00
FBgn0029927	CG14430	CG14430	2.28	0.00
FBgn0036773	CG13698	CG13698	2.28	0.00
FBgn0029093	cathD	cathD	2.28	0.00
FBgn0037770	Art4	Arginine methyltransferase 4	2.28	0.00
FBgn0032689	CG10413	CG10413	2.28	0.00
FBgn0015777	nrv2	nervana 2	2.27	0.00
FBgn0040950	CG13990	CG13990	2.27	0.00
FBgn0002732	malpha	E(spl) region transcript malpha	2.27	0.00
FBgn0035028	Start1	Start1	2.27	0.00
FBgn0033008	gus	gustavus	2.27	0.00
FBgn0035657	CG10478	CG10478	2.27	0.00
FBgn0031597	CG17612	CG17612	2.27	0.00
FBgn0026263	bip1	bip1	2.27	0.00
FBgn0031268	CG2813	CG2813	2.27	0.00
FBgn0033529	CG17765	CG17765	2.26	0.00
FBgn0039923	MED26	Mediator complex subunit 26	2.26	0.00
FBgn0037347	CG1427	CG1427	2.25	0.00
FBgn0038100	Paip2	polyA-binding protein interacting protein 2	2.25	0.00
FBgn0004592	Eig71Ee	Ecdysone-induced gene 71Ee	2.24	0.00
FBgn0042092	CG13773	CG13773	2.24	0.00
FBgn0032724	CG10428	CG10428	2.24	0.00
FBgn0005655	mus209	mutagen-sensitive 209	2.24	0.00

FBgn0024921	Trn	Transportin	2.24	0.00
FBgn0032988	Tif-IA	Tif-IA	2.24	0.00
FBgn0034440	CG10073	CG10073	2.24	0.02
FBgn0034082	CG10734	CG10734	2.23	0.00
FBgn0037344	CG2926	CG2926	2.23	0.00
FBgn0040466	Dip2	Dorsal interacting protein 2	2.23	0.00
FBgn0034264	CG10933	CG10933	2.23	0.00
FBgn0035440	CG14969	CG14969	2.22	0.00
FBgn0040941	CG15308	CG15308	2.22	0.00
FBgn0034943	Fmo-1	Flavin-containing monooxygenase 1	2.22	0.00
FBgn0039759	CG9733	CG9733	2.22	0.00
FBgn0027586	CG5867	CG5867	2.22	0.00
FBgn0002629	m4	E(spl) region transcript m4	2.21	0.00
FBgn0032727	CG10623	CG10623	2.21	0.00
FBgn0020240	Mcr	Macroglobulin complement-related	2.21	0.00
FBgn0015776	nrv1	nervana 1	2.21	0.00
FBgn0035550	CG11349	CG11349	2.21	0.00
FBgn0028980	tan	tantalus	2.21	0.00
FBgn0015791	Rab14	Rab-protein 14	2.21	0.00
FBgn0011291	Taf11	TBP-associated factor 11	2.20	0.00
FBgn0025373	Fpps	Farnesyl pyrophosphate synthase	2.20	0.00
FBgn0034756	Cyp6d2	Cyp6d2	2.20	0.00
FBgn0036062	CG6685	CG6685	2.20	0.00
FBgn0037680	CG8121	CG8121	2.20	0.00
FBgn0040324	Ephrin	Ephrin	2.20	0.00
FBgn0042189	CG17376	CG17376	2.20	0.00
FBgn0010379	Akt1	Akt1	2.20	0.00
FBgn0002734	HLHmdelta	E(spl) region transcript mdelta	2.20	0.00
FBgn0035038	CG13588	CG13588	2.19	0.00
FBgn0041710	yellow-f	yellow-f	2.19	0.00
FBgn0032514	CG9302	CG9302	2.19	0.00
FBgn0031717	Oscillin	Oscillin	2.19	0.00
FBgn0039928	cals	calsyntenin-1	2.19	0.00
FBgn0030828	CG5162	CG5162	2.19	0.00
FBgn0029167	Hml	Hemolectin	2.19	0.00
FBgn0035765	CG8600	CG8600	2.19	0.00

FBgn0028693	Rpn12	Rpn12	2.19	0.00
FBgn0035798	CG7526	CG7526	2.18	0.00
FBgn0053120	CG33120	CG33120	2.18	0.00
FBgn0031044	CG14211	CG14211	2.18	0.00
FBgn0032680	Ntf-2r	Nuclear transport factor-2-related	2.18	0.00
FBgn0035679	CG10467	CG10467	2.18	0.00
FBgn0037971	CG10007	CG10007	2.17	0.00
FBgn0034894	CG4091	CG4091	2.17	0.00
FBgn0028472	dbr	debra	2.17	0.00
FBgn0036221	CG11588	CG11588	2.17	0.00
FBgn0036126	CG6272	CG6272	2.17	0.00
FBgn0003891	tud	tudor	2.17	0.00
FBgn0034804	CG3831	CG3831	2.17	0.00
FBgn0051111	CG31111	CG31111	2.17	0.00
FBgn0035398	CG1869	CG1869	2.17	0.00
FBgn0040211	hgo	homogentisate 1,2-dioxygenase	2.16	0.00
FBgn0032197	CG5694	CG5694	2.16	0.00
FBgn0011253	gish	gilgamesh	2.16	0.00
FBgn0034290	CG5773	CG5773	2.16	0.00
FBgn0027945	ppl	pumpless	2.16	0.00
FBgn0020257	ppa	partner of paired	2.16	0.00
FBgn0000259	CkIIbeta	Casein kinase II beta subunit	2.15	0.00
FBgn0027865	Tsp96F	Tetraspanin 96F	2.15	0.00
FBgn0040002	CG17683	CG17683	2.15	0.00
FBgn0005612	Sox14	Sox box protein 14	2.15	0.00
FBgn0032283	CG7296	CG7296	2.15	0.00
FBgn0030364	CG15735	CG15735	2.15	0.00
FBgn0029838	CG4666	CG4666	2.14	0.00
FBgn0039798	CG11313	CG11313	2.14	0.00
FBgn0000567	Eip74EF	Ecdysone-induced protein 74EF	2.14	0.00
FBgn0004898	fd96Cb	forkhead domain 96Cb	2.14	0.00
FBgn0031515	CG9664	CG9664	2.14	0.00
FBgn0033641	Roc2	Roc2	2.14	0.00
FBgn0052210	CG32210	CG32210	2.13	0.00
FBgn0004106	cdc2	cdc2	2.13	0.00
FBgn0041184	Socs36E	Suppressor of cytokine signaling at 36E	2.13	0.00

FBgn0032132	CG4382	CG4382	2.12	0.00
FBgn0031992	CG8498	CG8498	2.12	0.00
FBgn0037794	CG6254	CG6254	2.12	0.00
FBgn0026404	Nc	Nedd2-like caspase	2.12	0.00
FBgn0011762	DNApol-alpha50	DNA polymerase alpha 50kD	2.12	0.00
FBgn0036432	shd	shade	2.12	0.00
FBgn0031834	CG13766	CG13766	2.11	0.00
FBgn0025741	plexA	plexin A	2.11	0.00
FBgn0002741	Mhc	Myosin heavy chain	2.11	0.01
FBgn0039014	CG6982	CG6982	2.11	0.00
FBgn0034398	CG15098	CG15098	2.11	0.00
FBgn0052632	CG32632	CG32632	2.11	0.00
FBgn0027375	RecQ5	homolog of RecQ	2.11	0.00
FBgn0036573	CG5161	CG5161	2.11	0.00
FBgn0032248	CG5343	CG5343	2.11	0.00
FBgn0031962	CG18591	CG18591	2.11	0.00
FBgn0031335	CG5565	CG5565	2.11	0.00
FBgn0052268	dro6	drosomycin-6	2.11	0.00
FBgn0037121	Rpb8	Rpb8	2.11	0.00
FBgn0037670	CG8436	CG8436	2.10	0.00
FBgn0036787	CG4306	CG4306	2.10	0.00
FBgn0024248	chico	chico	2.10	0.00
FBgn0032371	CG4983	CG4983	2.10	0.00
FBgn0029825	CG12728	CG12728	2.10	0.00
FBgn0039904	Hcf	Host cell factor	2.10	0.00
FBgn0036121	CG6310	CG6310	2.10	0.00
FBgn0002535	Lcp4	Larval cuticle protein 4	2.10	0.00
FBgn0014879	Set	Set	2.10	0.00
FBgn0030309	CG1572	CG1572	2.10	0.00
FBgn0035469	CG14977	CG14977	2.09	0.00
FBgn0024320	NPC1	Niemann-Pick Type C-1	2.09	0.00
FBgn0035526	CG1316	CG1316	2.09	0.00
FBgn0034282	Mapmodulin	Mapmodulin	2.09	0.00
FBgn0033731	CG8515	CG8515	2.09	0.00
FBgn0039959	CG17514	CG17514	2.09	0.00
FBgn0020616	SA	Stromalin	2.09	0.00

FBgn0040483	Caps	Calcium activated protein for secretion	2.09	0.00
FBgn0039274	CG11920	CG11920	2.08	0.00
FBgn0037422	Osi13	Osiris 13	2.08	0.00
FBgn0032726	CG10621	CG10621	2.08	0.00
FBgn0027095	ARP-like	ARP-like	2.08	0.00
FBgn0011217	eff	effete	2.08	0.00
FBgn0030740	CG9917	CG9917	2.08	0.00
FBgn0014007	Ptp69D	Protein tyrosine phosphatase 69D	2.07	0.00
FBgn0004419	me31B	maternal expression at 31B	2.07	0.00
FBgn0037960	mthl5	methuselah-like 5	2.07	0.00
FBgn0030863	CG8188	CG8188	2.07	0.00
FBgn0031560	CG16713	CG16713	2.06	0.00
FBgn0034742	CG4294	CG4294	2.06	0.00
FBgn0028545	CG8942	CG8942	2.06	0.00
FBgn0026199	myoglianin	myoglianin	2.06	0.00
FBgn0003498	sqd	squid	2.06	0.00
FBgn0000307	chif	chiffon	2.06	0.00
FBgn0039695	CG12068	CG12068	2.06	0.00
FBgn0023023	CRMP	Collapsin Response Mediator Protein	2.05	0.00
FBgn0034724	CG3624	CG3624	2.05	0.00
FBgn0031325	CG14343	CG14343	2.05	0.00
FBgn0033020	CG10396	CG10396	2.05	0.00
FBgn0040534	CG11985	CG11985	2.05	0.00
FBgn0033528	translin	translin	2.05	0.00
FBgn0035844	CG13676	CG13676	2.05	0.00
FBgn0032251	CG13142	CG13142	2.05	0.00
FBgn0024989	CG3777	CG3777	2.05	0.00
FBgn0025874	Meics	Meiotic central spindle	2.05	0.00
FBgn0031547	Sr-CIV	Scavenger receptor class C, type IV	2.05	0.00
FBgn0032887	CG33322	CG33322	2.05	0.00
FBgn0051999	CG31999	CG31999	2.04	0.00
FBgn0035289	CG12026	CG12026	2.04	0.00
FBgn0037551	CG7891	CG7891	2.04	0.00
FBgn0028506	CG4455	CG4455	2.04	0.00
FBgn0000565	Eip71CD	Ecdysone-induced protein 28/29kD	2.04	0.00
FBgn0032242	CG5355	CG5355	2.04	0.00

FBgn0024330	MED6	Mediator complex subunit 6	2.04	0.00
FBgn0015393	hoip	hoi-polloi	2.04	0.00
FBgn0050365	CG30365	CG30365	2.04	0.00
FBgn0051729	CG31729	CG31729	2.03	0.00
FBgn0034275	CG5002	CG5002	2.03	0.00
FBgn0036921	RhoGDI	RhoGDI	2.03	0.00
FBgn0032755	CG17344	CG17344	2.03	0.00
FBgn0039113	CG10217	CG10217	2.03	0.00
FBgn0037575	Ctr1B	Copper transporter 1B	2.03	0.00
FBgn0030398	CG2555	CG2555	2.03	0.00
FBgn0040371	CG12470	CG12470	2.02	0.00
FBgn0039671	CG11470	CG11470	2.02	0.00
FBgn0031961	CG7102	CG7102	2.02	0.00
FBgn0000634	Fas1	Fasciclin 1	2.02	0.00
FBgn0033055	CG7861	CG7861	2.02	0.00
FBgn0053180	Ranbp16	Ranbp16	2.02	0.00
FBgn0036926	CG7646	CG7646	2.02	0.00
FBgn0052158	CG32158	CG32158	2.02	0.00
FBgn0036266	CG5626	CG5626	2.01	0.00
FBgn0027507	CG1344	CG1344	2.01	0.00
FBgn0031489	CG17224	CG17224	2.01	0.00
FBgn0030312	l(1)G0237	lethal (1) G0237	2.01	0.00
FBgn0032495	CG16820	CG16820	2.01	0.00
FBgn0030286	CG1657	CG1657	2.01	0.00
FBgn0036018	CG3335	CG3335	2.01	0.00
FBgn0020415	Idgf2	Imaginal disc growth factor 2	2.01	0.00
FBgn0015229	glec	gliolectin	2.01	0.00
FBgn0001078	ftz-f1	ftz transcription factor 1	2.01	0.00
FBgn0041191	Rheb	Rheb	2.01	0.00
FBgn0038489	CG12265	CG12265	2.01	0.00
FBgn0004629	Cys	Cystatin-like	2.00	0.00
FBgn0033495	CG12214	CG12214	2.00	0.00
FBgn0034703	CG3045	CG3045	2.00	0.00
FBgn0028984	Spn5	Serine protease inhibitor 5	2.00	0.00
FBgn0000422	Ddc	Dopa decarboxylase	2.00	0.00
FBgn0031879	SP1070	SP1070	2.00	0.00

FBgn0040102	lectin-24Db	lectin-24Db	2.00	0.00
FBgn0004648	svr	silver	2.00	0.00
FBgn0032139	CG13116	CG13116	2.00	0.00
FBgn0035110	thoc7	thoc7	1.99	0.00
FBgn0000229	bsk	basket	1.99	0.00
FBgn0029002	miple2	miple2	1.99	0.00
FBgn0032421	CG17218	CG17218	1.99	0.00
FBgn0001491	l(1)10Bb	lethal (1) 10Bb	1.99	0.00
FBgn0030693	CG8974	CG8974	1.99	0.00
FBgn0034614	CG9752	CG9752	1.98	0.00
FBgn0037336	CG2519	CG2519	1.98	0.00
FBgn0037755	CG12945	CG12945	1.98	0.00
FBgn0031326	CG5156	CG5156	1.98	0.00
FBgn0028734	Fmr1	Fmr1	1.98	0.00
FBgn0037338	Snm1	Snm1	1.98	0.00
FBgn0035817	CG7409	CG7409	1.98	0.00
FBgn0036967	CG6597	CG6597	1.98	0.00
FBgn0001168	h	hairy	1.98	0.00
FBgn0038242	CG14852	CG14852	1.98	0.00
FBgn0031453	CG9894	CG9894	1.98	0.00
FBgn0035710	SP1173	SP1173	1.98	0.00
FBgn0032699	CG10383	CG10383	1.97	0.00
FBgn0031689	Cyp28d1	Cyp28d1	1.97	0.00
FBgn0037697	CG9363	CG9363	1.97	0.00
FBgn0026371	SAK	SAK	1.97	0.00
FBgn0025809	Paf-AHalpha	Platelet-activating factor acetylhydrolase alpha	1.97	0.00
FBgn0051948	CG31948	CG31948	1.97	0.00
FBgn0040252	Ugt86Dh	Ugt86Dh	1.97	0.00
FBgn0052081	CG32081	CG32081	1.97	0.00
FBgn0035427	CG14959	CG14959	1.97	0.00
FBgn0034554	CG15227	CG15227	1.97	0.02
FBgn0013746	alien	alien	1.97	0.00
FBgn0036715	Cad74A	Cad74A	1.97	0.00
FBgn0032521	CG7110	CG7110	1.96	0.00
FBgn0051510	CG31510	CG31510	1.96	0.00
FBgn0000053	ade3	adenosine 3	1.96	0.00

FBgn0041160	comm2	comm2	1.96	0.00
FBgn0032000	CG8372	CG8372	1.96	0.00
FBgn0037378	CG2046	CG2046	1.96	0.00
FBgn0035839	CG7550	CG7550	1.96	0.00
FBgn0032691	CG10414	CG10414	1.96	0.00
FBgn0039135	CG13603	CG13603	1.96	0.00
FBgn0028704	Nckx30C	Nckx30C	1.95	0.00
FBgn0032880	CG9318	CG9318	1.95	0.00
FBgn0029764	CG3249	CG3249	1.95	0.00
FBgn0037128	CG14572	CG14572	1.95	0.00
FBgn0036295	sti	sticky	1.95	0.00
FBgn0033301	CG12780	CG12780	1.95	0.00
FBgn0053096	CG33096	CG33096	1.95	0.00
FBgn0037360	CG2182	CG2182	1.95	0.00
FBgn0028406	Rep4	Rep4	1.95	0.00
FBgn0004432	Cyp1	Cyclophilin 1	1.94	0.00
FBgn0029506	Tsp42Ee	Tetraspanin 42Ee	1.94	0.00
FBgn0030703	CG9066	CG9066	1.94	0.00
FBgn0032685	CG10211	CG10211	1.94	0.00
FBgn0039265	CG11790	CG11790	1.94	0.00
FBgn0035608	CG10630	CG10630	1.94	0.00
FBgn0036959	CG6951	CG6951	1.94	0.00
FBgn0036911	Fibp	Fibp	1.94	0.00
FBgn0035976	PGRP-LC	Peptidoglycan recognition protein LC	1.94	0.00
FBgn0015795	Rab7	Rab-protein 7	1.94	0.00
FBgn0022347	CG11971	CG11971	1.94	0.00
FBgn0040701	CG13714	CG13714	1.94	0.00
FBgn0051751	CG31751	CG31751	1.94	0.00
FBgn0032624	CG6304	CG6304	1.93	0.00
FBgn0010452	trn	tartan	1.93	0.00
FBgn0034179	CG6805	CG6805	1.93	0.00
FBgn0052084	CG33269	CG33269	1.93	0.00
FBgn0035393	CG16753	CG16753	1.93	0.00
FBgn0034791	CG3501	CG3501	1.93	0.00
FBgn0038243	CG8066	CG8066	1.93	0.00
FBgn0050437	CG30437	CG30437	1.93	0.00

FBgn0034668	Fili	Fish-lips	1.93	0.00
FBgn0039532	Mtl	Mig-2-like	1.93	0.00
FBgn0002735	HLHmgamma	E(spl) region transcript mgamma	1.92	0.00
FBgn0038471	CG5220	CG5220	1.92	0.00
FBgn0030306	CG1751	CG1751	1.92	0.00
FBgn0031571	CG3921	CG3921	1.92	0.00
FBgn0037552	CG7800	CG7800	1.92	0.00
FBgn0034797	nahoda	nahoda	1.92	0.00
FBgn0010435	emp	epithelial membrane protein	1.92	0.00
FBgn0024698	cpsf	cleavage and polyadenylation specificity factor	1.92	0.00
FBgn0033170	sPLA2	secretory Phospholipase A2	1.91	0.00
FBgn0025781	cdc16	cdc16	1.91	0.00
FBgn0032194	CG4901	CG4901	1.91	0.00
FBgn0032305	CG6700	CG6700	1.91	0.00
FBgn0001225	Hsp26	Heat shock protein 26	1.91	0.00
FBgn0031529	CG9662	CG9662	1.91	0.00
FBgn0039330	CG11909	CG11909	1.91	0.02
FBgn0031361	CG17652	CG17652	1.91	0.00
FBgn0020224	Cbl	Cbl	1.91	0.00
FBgn0030994	CG14193	CG14193	1.91	0.00
FBgn0032214	CG4968	CG4968	1.91	0.00
FBgn0038525	CG14329	CG14329	1.91	0.00
FBgn0030429	CG4661	CG4661	1.91	0.00
FBgn0003292	rt	rotated abdomen	1.90	0.00
FBgn0033028	CG11665	CG11665	1.90	0.00
FBgn0037560	CG18228	CG18228	1.90	0.00
FBgn0031822	CG9548	CG9548	1.90	0.00
FBgn0038225	CG8489	CG8489	1.90	0.00
FBgn0004624	CaMKII	Calcium/calmodulin-dependent protein kinase II	1.90	0.00
FBgn0036931	CG14183	CG14183	1.90	0.00
FBgn0028510	CG15261	CG15261	1.90	0.00
FBgn0038947	sar1	sar1	1.90	0.00
FBgn0000261	Cat	Catalase	1.90	0.00
FBgn0036538	CG15715	CG15715	1.90	0.00
FBgn0035523	CG1311	CG1311	1.90	0.00
FBgn0011722	Tig	Tiggrin	1.90	0.00

FBgn0036565	CG5235	CG5235	1.90	0.00
FBgn0015924	crq	croquemort	1.90	0.00
FBgn0025821	I-t	inhibitor-t	1.90	0.00
FBgn0001229	Hsp67Bc	Heat shock gene 67Bc	1.90	0.00
FBgn0040512	zetaCOP	zetaCOP	1.90	0.00
FBgn0032193	CG5727	CG5727	1.89	0.00
FBgn0037468	CG1943	CG1943	1.89	0.00
FBgn0016724	RfaBp	Retinoid- and fatty-acid binding protein	1.89	0.00
FBgn0030334	Karl	Karl	1.89	0.00
FBgn0037721	CG9427	CG9427	1.89	0.00
FBgn0036008	CG3408	CG3408	1.89	0.00
FBgn0032601	yellow-b	yellow-b	1.89	0.00
FBgn0039281	CG11847	CG11847	1.89	0.00
FBgn0037082	CG5664	CG5664	1.89	0.00
FBgn0033468	CG1418	CG1418	1.89	0.00
FBgn0037067	CG11310	CG11310	1.89	0.00
FBgn0033683	CG18343	CG18343	1.89	0.00
FBgn0035726	CG9953	CG9953	1.88	0.00
FBgn0030657	CG15645	CG15645	1.88	0.00
FBgn0028886	CG15279	CG15279	1.88	0.00
FBgn0035830	CG8209	CG8209	1.88	0.00
FBgn0032358	CG4851	CG4851	1.88	0.00
FBgn0038453	CG10326	CG10326	1.88	0.00
FBgn0036843	CG6812	CG6812	1.88	0.00
FBgn0036830	CG14077	CG14077	1.88	0.00
FBgn0035800	CG7716	CG7716	1.88	0.00
FBgn0038978	CG7045	CG7045	1.88	0.00
FBgn0037288	CG14661	CG14661	1.88	0.00
FBgn0032200	CG5676	CG5676	1.88	0.00
FBgn0036942	CG7328	CG7328	1.88	0.00
FBgn0032805	CG10337	CG10337	1.88	0.00
FBgn0037534	CG2781	CG2781	1.88	0.00
FBgn0010894	sinu	sinuous	1.88	0.00
FBgn0038966	CG13848	CG13848	1.87	0.00
FBgn0037518	CG2641	CG2641	1.87	0.00
FBgn0030183	CG15309	CG15309	1.87	0.00

FBgn0036248	CG17153	CG17153	1.87	0.00
FBgn0031986	CG8673	CG8673	1.87	0.00
FBgn0032285	CG17108	CG17108	1.87	0.00
FBgn0031581	CG10039	CG10039	1.87	0.00
FBgn0028990	Spn27A	Serpin-27A	1.87	0.00
FBgn0052372	CG32372	CG32372	1.87	0.00
FBgn0040827	CG13315	CG13315	1.87	0.00
FBgn0040519	CG15219	CG15219	1.87	0.00
FBgn0038359	CG5614	CG5614	1.87	0.00
FBgn0030341	CG1967	CG1967	1.87	0.00
FBgn0004581	bgcn	benign gonial cell neoplasm	1.87	0.00
FBgn0036005	CG3428	CG3428	1.87	0.00
FBgn0039268	CG11819	CG11819	1.87	0.00
FBgn0034726	Mes4	Mes4	1.87	0.00
FBgn0022942	Cbp80	cap binding protein 80	1.87	0.00
FBgn0035132	mthl10	methuselah-like 10	1.87	0.00
FBgn0050080	CG30080	CG30080	1.87	0.00
FBgn0035983	CG4080	CG4080	1.87	0.00
FBgn0030247	CG15208	CG15208	1.87	0.00
FBgn0022213	Cas	CAS/CSE1 segregation protein	1.87	0.00
FBgn0012066	DNApol-delta	DNA-polymerase-delta	1.87	0.00
FBgn0032899	CG9338	CG9338	1.86	0.00
FBgn0039690	CG1969	CG1969	1.86	0.00
FBgn0035904	CG6776	CG6776	1.86	0.00
FBgn0001104	G-ialpha65A	G protein alphai subunit 65A	1.86	0.00
FBgn0036848	CG10424	CG10424	1.86	0.00
FBgn0036014	CG3222	CG3222	1.86	0.00
FBgn0031974	CG12560	CG12560	1.86	0.00
FBgn0010348	Arf79F	ADP ribosylation factor 79F	1.85	0.00
FBgn0028872	CG18095	CG18095	1.85	0.00
FBgn0033015	d4	d4	1.85	0.00
FBgn0013981	His4r	Histone H4 replacement	1.85	0.00
FBgn0026084	cib	ciboulot	1.85	0.00
FBgn0032723	CG18397	CG18397	1.85	0.00
FBgn0031892	CG13783	CG13783	1.85	0.00
FBgn0052016	CG32016	CG32016	1.85	0.00

FBgn0000547	ed	echinoid	1.85	0.00
FBgn0029121	Sras	severas	1.85	0.00
FBgn0035496	CG14990	CG14990	1.85	0.00
FBgn0030852	CG8316	CG8316	1.85	0.00
FBgn0032253	CG5322	CG5322	1.85	0.00
FBgn0036309	CG10971	CG10971	1.85	0.00
FBgn0037834	Art1	Arginine methyltransferase 1	1.85	0.00
FBgn0038151	yellow-e2	yellow-e2	1.84	0.00
FBgn0037674	Vps16A	Vps16A	1.84	0.00
FBgn0027660	blot	bloated tubules	1.84	0.00
FBgn0040508	ACXC	ACXC	1.84	0.00
FBgn0032615	CG6012	CG6012	1.84	0.00
FBgn0031033	CG14219	CG14219	1.84	0.00
FBgn0034342	GstE8	Glutathione S transferase E8	1.84	0.00
FBgn0035871	CG7188	CG7188	1.84	0.00
FBgn0032124	CG17855	CG17855	1.84	0.00
FBgn0051935	CG31935	CG31935	1.84	0.00
FBgn0033013	Nipped-A	Nipped-A	1.84	0.00
FBgn0004436	UbcD6	Ubiquitin conjugating enzyme	1.84	0.00
FBgn0002791	mr	morula	1.84	0.00
FBgn0040009	CG17490	CG17490	1.83	0.00
FBgn0050344	CG30344	CG30344	1.83	0.00
FBgn0039332	CG11910	CG11910	1.83	0.00
FBgn0039370	CG4956	CG4956	1.83	0.00
FBgn0032059	CG9296	CG9296	1.83	0.00
FBgn0031676	CG14040	CG14040	1.83	0.00
FBgn0038294	Zeelin1	Zeelin1	1.83	0.00
FBgn0030711	CG8928	CG8928	1.83	0.00
FBgn0040323	GNBP1	Gram-negative bacteria binding protein 1	1.83	0.00
FBgn0036509	CG7739	CG7739	1.83	0.00
FBgn0001105	Gbeta13F	G protein beta-subunit 13F	1.83	0.00
FBgn0020416	Idgf1	Imaginal disc growth factor 1	1.83	0.00
FBgn0003607	Su(var)205	Suppressor of variegation 205	1.83	0.00
FBgn0035869	CG7066	CG7066	1.83	0.00
FBgn0026869	Thd1	Thd1	1.83	0.00
FBgn0024811	Crk	Crk	1.83	0.00

FBgn0033439	CG1773	CG1773	1.82	0.01
FBgn0035426	CG12078	CG12078	1.82	0.00
FBgn0037359	MED27	Mediator complex subunit 27	1.82	0.00
FBgn0030171	CG2989	CG2989	1.82	0.00
FBgn0002914	Myb	Myb oncogene-like	1.82	0.00
FBgn0040011	CG17494	CG17494	1.82	0.00
FBgn0035842	CG7504	CG7504	1.82	0.00
FBgn0039293	CG11851	CG11851	1.82	0.00
FBgn0028894	CG5869	CG5869	1.82	0.00
FBgn0034140	CG8317	CG8317	1.82	0.00
FBgn0039077	CG17380	CG17380	1.82	0.00
FBgn0004636	R	Roughened	1.82	0.00
FBgn0037677	CG12951	CG12951	1.82	0.00
FBgn0036147	CG6199	CG6199	1.82	0.00
FBgn0032906	CG9273	CG9273	1.82	0.00
FBgn0004373	fwd	four wheel drive	1.81	0.00
FBgn0041205	key	kenny	1.81	0.00
FBgn0028913	CG3473	CG3473	1.81	0.00
FBgn0030421	CG3812	CG3812	1.81	0.00
FBgn0053070	CG14425	CG14425	1.81	0.00
FBgn0022959	yps	ypsilon schachtel	1.81	0.00
FBgn0039800	CG11314	CG11314	1.81	0.00
FBgn0038250	CG3505	CG3505	1.81	0.00
FBgn0033031	CG8245	CG8245	1.81	0.00
FBgn0034729	CG10344	CG10344	1.81	0.00
FBgn0061209	His2B:CG17949	His2B:CG17949	1.81	0.00
FBgn0037537	CG2767	CG2767	1.81	0.00
FBgn0030985	Obp18a	Odorant-binding protein 18a	1.81	0.00
FBgn0035866	CG7197	CG7197	1.81	0.00
FBgn0051100	CG31100	CG31100	1.81	0.00
FBgn0035073	CG16896	CG16896	1.81	0.00
FBgn0035434	dro5	drosomycin-5	1.81	0.00
FBgn0030611	CG15027	CG15027	1.81	0.00
FBgn0000591	E(spl)	Enhancer of split	1.81	0.00
FBgn0030726	CG9216	CG9216	1.81	0.00
FBgn0025366	Ip259	Intronic Protein 259	1.81	0.00

FBgn0051367	CG31367	CG31367	1.81	0.00
FBgn0035998	CG3437	CG3437	1.81	0.00
FBgn0035725	CG18156	CG18156	1.81	0.00
FBgn0016675	Lectin-galC1	Galactose-specific C-type lectin	1.80	0.00
FBgn0031032	CG14204	CG14204	1.80	0.00
FBgn0038149	CG9796	CG9796	1.80	0.00
FBgn0040295	Ogt	O-glycosyltransferase	1.80	0.00
FBgn0011817	nmo	nemo	1.80	0.00
FBgn0030969	CG7288	CG7288	1.80	0.00
FBgn0011571	caz	cabeza	1.80	0.00
FBgn0040852	CG15197	CG15197	1.80	0.00
FBgn0003041	pbl	pebble	1.80	0.00
FBgn0036194	CG11652	CG11652	1.80	0.00
FBgn0000464	Lar	Leukocyte-antigen-related-like	1.80	0.00
FBgn0058016	CG17493	CG17493	1.79	0.00
FBgn0037730	CG9444	CG9444	1.79	0.00
FBgn0036932	CG14184	CG14184	1.79	0.00
FBgn0033021	CG10417	CG10417	1.79	0.00
FBgn0036710	CG6479	CG6479	1.79	0.00
FBgn0036499	CG7276	CG7276	1.79	0.00
FBgn0036550	CG17026	CG17026	1.79	0.00
FBgn0030698	Aats-arg	Arginyl-tRNA synthetase	1.79	0.00
FBgn0010406	RNaseX25	Ribonuclease X25	1.79	0.00
FBgn0039518	CG13978	CG13978	1.79	0.00
FBgn0014469	Cyp4e2	Cytochrome P450-4e2	1.79	0.00
FBgn0052590	CG32590	CG32590	1.79	0.00
FBgn0038110	CG8031	CG8031	1.79	0.00
FBgn0011570	cpb	capping protein beta	1.79	0.00
FBgn0030870	CG6398	CG6398	1.79	0.00
FBgn0010341	Cdc42	Cdc42	1.79	0.00
FBgn0031351	CG14352	CG14352	1.79	0.00
FBgn0030653	CG7860	CG7860	1.79	0.00
FBgn0052694	CG32694	CG32694	1.79	0.00
FBgn0036454	CG17839	CG17839	1.79	0.00
FBgn0031186	CG14614	CG14614	1.78	0.00
FBgn0031908	CG5177	CG5177	1.78	0.00

FBgn0032956	cul-2	cul-2	1.78	0.00
FBgn0035080	pain	painless	1.78	0.00
FBgn0025355	SuUR	Suppressor of Under-Replication	1.78	0.00
FBgn0024222	ird5	immune response deficient 5	1.78	0.00
FBgn0003321	sbr	small bristles	1.78	0.00
FBgn0030029	CG15343	CG15343	1.78	0.00
FBgn0036465	CG11552	CG11552	1.78	0.00
FBgn0051108	CG31108	CG31108	1.78	0.00
FBgn0036898	Oat	Ornithine aminotransferase precursor	1.78	0.01
FBgn0037719	bocksbeutel	bocksbeutel	1.78	0.00
FBgn0004513	Mdr65	Multiple drug resistance 65	1.78	0.00
FBgn0035151	CG17129	CG17129	1.78	0.00
FBgn0034008	CG8152	CG8152	1.78	0.00
FBgn0036029	CG16719	CG16719	1.78	0.00
FBgn0038198	CG3153	CG3153	1.77	0.00
FBgn0028371	jbug	jitterbug	1.77	0.00
FBgn0030307	CG33235	CG33235	1.77	0.00
FBgn0033686	CG12367	CG12367	1.77	0.00
FBgn0030136	RpS28b	Ribosomal protein S28b	1.77	0.00
FBgn0022986	qkr58E-1	quaking related 58E-1	1.77	0.00
FBgn0035097	CG13405	CG13405	1.77	0.00
FBgn0029725	CG2871	CG2871	1.77	0.00
FBgn0034663	CG4363	CG4363	1.77	0.00
FBgn0039955	CG41099	CG41099	1.77	0.00
FBgn0032864	CG2493	CG2493	1.77	0.00
FBgn0002780	mod	modulo	1.77	0.00
FBgn0032590	CG4631	CG4631	1.77	0.00
FBgn0023076	Clk	Clock	1.77	0.00
FBgn0032599	CG13277	CG13277	1.77	0.00
FBgn0002506	did	diminished discs	1.77	0.00
FBgn0034307	CG10914	CG10914	1.77	0.00
FBgn0011569	can	cannonball	1.77	0.00
FBgn0032491	CG16815	CG16815	1.77	0.00
FBgn0030376	CG2750	CG2750	1.77	0.00
FBgn0040057	eIF-4B	Eukaryotic initiation factor 4B	1.76	0.00
FBgn0030744	CG9992	CG9992	1.76	0.00

FBgn0028535	CG4140	CG4140	1.76	0.00
FBgn0037860	CG6629	CG6629	1.76	0.00
FBgn0035158	CG13895	CG13895	1.76	0.00
FBgn0003277	RpII215	RNA polymerase II 215kD subunit	1.76	0.00
FBgn0035838	CG7942	CG7942	1.76	0.00
FBgn0038511	CG5873	CG5873	1.76	0.00
FBgn0030782	CG18358	CG18358	1.76	0.00
FBgn0032259	CG6144	CG6144	1.76	0.00
FBgn0030854	CG8289	CG8289	1.76	0.00
FBgn0000370	crc	cryptocephal	1.76	0.00
FBgn0039291	CG13663	CG13663	1.76	0.00
FBgn0002521	pho	pleiohomeotic	1.76	0.00
FBgn0030694	CG15602	CG15602	1.76	0.00
FBgn0011509	SrpRbeta	Signal recognition particle receptor beta	1.76	0.00
FBgn0004176	gammaTub23C	gamma-Tubulin at 23C	1.76	0.00
FBgn0052573	CG32573	CG32573	1.76	0.00
FBgn0037669	CG9740	CG9740	1.76	0.00
FBgn0037126	CG14567	CG14567	1.75	0.00
FBgn0032664	CG5755	CG5755	1.75	0.00
FBgn0030699	CG8578	CG8578	1.75	0.00
FBgn0037251	CG9804	CG9804	1.75	0.00
FBgn0000404	CycA	Cyclin A	1.75	0.00
FBgn0030890	CG7536	CG7536	1.75	0.00
FBgn0034232	CG4866	CG4866	1.75	0.00
FBgn0051990	CG17768	CG17768	1.75	0.00
FBgn0032215	CG5385	CG5385	1.75	0.00
FBgn0031417	CG3597	CG3597	1.75	0.00
FBgn0040551	CG11686	CG11686	1.75	0.00
FBgn0011703	RnrL	Ribonucleoside diphosphate reductase large subunit	1.75	0.00
FBgn0001123	G-salpha60A	G protein salpha 60A	1.75	0.00
FBgn0031600	CG3652	CG3652	1.75	0.00
FBgn0051624	CG31624	CG31624	1.75	0.00
FBgn0032960	Grp1	General receptor for phosphoinositides 1	1.75	0.00
FBgn0031951	r2d2	r2d2	1.75	0.00
FBgn0032840	sNPF	short neuropeptide F precursor	1.75	0.00
FBgn0052956	Chrac-14	Chrac-14	1.74	0.00

FBgn0025806	Rap2l	Ras-associated protein 2-like	1.74	0.00
FBgn0017577	Mcm5	Minichromosome maintenance 5	1.74	0.00
FBgn0039271	CG11839	CG11839	1.74	0.00
FBgn0033958	CG12858	CG12858	1.74	0.00
FBgn0027057	CSN1b	COP9 complex homolog subunit 1 b	1.74	0.00
FBgn0016059	Sema-1b	Sema-1b	1.74	0.00
FBgn0028380	fal	falten	1.74	0.00
FBgn0034685	CG6393	CG6393	1.74	0.00
FBgn0030355	CG2467	CG2467	1.74	0.00
FBgn0035906	CG6673	CG6673	1.74	0.00
FBgn0039463	CG18472	CG18472	1.74	0.00
FBgn0032004	CG8292	CG8292	1.74	0.00
FBgn0030296	CG15196	CG15196	1.74	0.00
FBgn0004087	Dhfr	Dihydrofolate reductase	1.74	0.00
FBgn0032816	CG10447	CG10447	1.74	0.00
FBgn0010218	Cpn	Calphotin	1.74	0.00
FBgn0033207	CG12826	CG12826	1.74	0.00
FBgn0034564	CG9344	CG9344	1.73	0.00
FBgn0032196	CG5708	CG5708	1.73	0.00
FBgn0052138	CG32138	CG32138	1.73	0.00
FBgn0014033	Sr-CI	Scavenger receptor class C, type I	1.73	0.00
FBgn0035585	CG12027	CG12027	1.73	0.00
FBgn0032900	CG14401	CG14401	1.73	0.00
FBgn0025726	unc-13	unc-13	1.73	0.00
FBgn0039136	CG5902	CG5902	1.73	0.00
FBgn0025879	Timp	Tissue inhibitor of metalloproteases	1.73	0.00
FBgn0032873	CG2614	CG2614	1.73	0.00
FBgn0052922	CG14681	CG14681	1.73	0.00
FBgn0026585	Ent2	Equilibrative nucleoside transporter 2	1.73	0.00
FBgn0051258	CG31258	CG31258	1.73	0.00
FBgn0036821	CG3961	CG3961	1.73	0.00
FBgn0052423	CG32423	CG32423	1.73	0.00
FBgn0015299	Ssb-c31a	Single stranded-binding protein c31A	1.73	0.00
FBgn0037638	CG8379	CG8379	1.73	0.00
FBgn0016715	Reg-2	Rhythmically expressed gene 2	1.73	0.00
FBgn0035764	mus312	mutagen-sensitive 312	1.73	0.00

FBgn0032343	CG6201	CG6201	1.73	0.00
FBgn0003008	or	orange	1.73	0.00
FBgn0031317	CG5118	CG5118	1.72	0.00
FBgn0036537	CG18081	CG18081	1.72	0.00
FBgn0028743	CG5036	CG5036	1.72	0.00
FBgn0039352	CG5053	CG5053	1.72	0.00
FBgn0017551	Rca1	Regulator of cyclin A1	1.72	0.00
FBgn0031626	CG15631	CG15631	1.72	0.00
FBgn0002534	Lcp3	Larval cuticle protein 3	1.72	0.00
FBgn0025574	Pli	Pellino	1.72	0.00
FBgn0031776	CG13993	CG13993	1.72	0.00
FBgn0030180	CG12102	CG12102	1.72	0.00
FBgn0031494	CG17219	CG17219	1.72	0.00
FBgn0002284	Pros26	Proteasome 26kD subunit	1.72	0.00
FBgn0031542	CG15414	CG15414	1.72	0.00
FBgn0036323	CG14118	CG14118	1.72	0.00
FBgn0028435	Su(Tpl)	Su(Tpl)	1.72	0.00
FBgn0032101	CG9586	CG9586	1.72	0.00
FBgn0039234	nct	nicastrin	1.72	0.00
FBgn0036811	MED11	Mediator complex subunit 11	1.72	0.00
FBgn0022131	aPKC	atypical protein kinase C	1.72	0.00
FBgn0036485	FucTA	FucTA	1.72	0.02
FBgn0037601	Cyp313b1	Cyp313b1	1.72	0.00
FBgn0036988	CG5262	CG5262	1.72	0.00
FBgn0011598	grp	grapes	1.72	0.00
FBgn0028428	Ih	I[[h]] channel	1.72	0.00
FBgn0050222	CG30222	CG30222	1.72	0.00
FBgn0070364	Dpse\GA10306	GA10306	1.71	0.02
FBgn0028738	ETH	Ecdysis triggering hormone	1.71	0.00
FBgn0010315	CycD	Cyclin D	1.71	0.00
FBgn0027890	dUTPase	Deoxyuridine triphosphatase	1.71	0.00
FBgn0039128	CG13599	CG13599	1.71	0.00
FBgn0032847	Taf13	TBP-associated factor 13	1.71	0.00
FBgn0031006	rictor	rapamycin-insensitive companion of Tor	1.71	0.00
FBgn0031034	CG14205	CG14205	1.71	0.00
FBgn0032524	CG9267	CG9267	1.71	0.00

FBgn0030710	CG8924	CG8924	1.71	0.00
FBgn0026313	X11L	X11L	1.71	0.00
FBgn0030280	CG11106	CG11106	1.71	0.00
FBgn0051391	CG31391	CG31391	1.71	0.00
FBgn0032482	Pect	Phosphoethanolamine cytidylyltransferase	1.71	0.00
FBgn0034443	cer	crammer	1.71	0.05
FBgn0030955	CG6891	CG6891	1.71	0.00
FBgn0050086	CG33460	CG33460	1.71	0.00
FBgn0013725	phyl	phyllopod	1.71	0.00
FBgn0039511	CG3330	CG3330	1.71	0.00
FBgn0026749	Yippee	Yippee	1.71	0.00
FBgn0031126	Cyp6v1	Cyp6v1	1.70	0.00
FBgn0013347	TfIIA-S	Transcription-factor-IIA-S	1.70	0.00
FBgn0033948	CG12863	CG12863	1.70	0.00
FBgn0034451	CG11242	CG11242	1.70	0.00
FBgn0028892	CG4161	CG4161	1.70	0.00
FBgn0032140	CG13117	CG13117	1.70	0.00
FBgn0051651	pgant5	polypeptide GalNAc transferase 5	1.70	0.00
FBgn0035488	CG11593	CG11593	1.70	0.00
FBgn0032876	CG1962	CG1962	1.70	0.00
FBgn0026777	Rad23	Rad23	1.70	0.00
FBgn0035898	CG6915	CG6915	1.70	0.00
FBgn0032632	CG6380	CG6380	1.70	0.00
FBgn0004908	Arf84F	ADP ribosylation factor 84F	1.70	0.00
FBgn0030625	CG5877	CG5877	1.70	0.00
FBgn0040239	bc10	bc10	1.70	0.00
FBgn0031229	CG3436	CG3436	1.70	0.00
FBgn0024230	Hs2st	Hs2st	1.70	0.00
FBgn0036437	CG5048	CG5048	1.70	0.00
FBgn0028901	CG18109	CG18109	1.70	0.00
FBgn0036918	CG7770	CG7770	1.70	0.00
FBgn0036820	CG6852	CG6852	1.70	0.00
FBgn0001309	kis	kismet	1.70	0.00
FBgn0037248	CG9809	CG9809	1.70	0.00
FBgn0033899	CG13016	CG13016	1.70	0.00
FBgn0034759	CG13511	CG13511	1.69	0.00

FBgn0001254	ImpE2	Ecdysone-inducible gene E2	1.69	0.00
FBgn0032014	CG7840	CG7840	1.69	0.00
FBgn0022936	CycH	Cyclin H	1.69	0.00
FBgn0037995	CG3809	CG3809	1.69	0.00
FBgn0015218	eIF-4E	Eukaryotic initiation factor 4E	1.69	0.00
FBgn0051547	CG31547	CG31547	1.69	0.00
FBgn0002543	lea	leak	1.69	0.00
FBgn0051380	CG31380	CG31380	1.69	0.00
FBgn0031376	CG10908	CG10908	1.69	0.00
FBgn0029866	CG3842	CG3842	1.69	0.00
FBgn0035964	Dhpr	Dihydropteridine reductase	1.69	0.00
FBgn0039031	CG17244	CG17244	1.69	0.00
FBgn0011230	poe	purity of essence	1.69	0.00
FBgn0053208	MICAL	MICAL	1.69	0.00
FBgn0036956	CG13813	CG13813	1.69	0.00
FBgn0015778	rin	rasputin	1.69	0.00
FBgn0036299	Tsf2	Transferrin 2	1.69	0.00
FBgn0029728	CG2861	CG2861	1.69	0.00
FBgn0036356	CG10222	CG10222	1.69	0.00
FBgn0022341	CG33521	CG33521	1.69	0.01
FBgn0030597	Eo	Ecdysone oxidase	1.68	0.00
FBgn0000711	flw	flap wing	1.68	0.00
FBgn0029990	CG2233	CG2233	1.68	0.00
FBgn0026563	CG1979	CG1979	1.68	0.00
FBgn0025549	unc-119	unc-119	1.68	0.00
FBgn0041180	TepIV	Thiolester containing protein IV	1.68	0.00
FBgn0032946	nrv3	nervana 3	1.68	0.00
FBgn0000546	EcR	Ecdysone receptor	1.68	0.00
FBgn0016983	smid	smallminded	1.68	0.00
FBgn0002968	Nrg	Neuroglian	1.68	0.02
FBgn0037956	CG6959	CG6959	1.68	0.00
FBgn0052075	CG32075	CG32075	1.68	0.00
FBgn0032693	Cyp310a1	Cyp310a1	1.68	0.00
FBgn0052666	CG32666	CG32666	1.68	0.00
FBgn0020633	Mcm7	Minichromosome maintenance 7	1.68	0.00
FBgn0051275	CG31275	CG31275	1.68	0.00

FBgn0036489	CG7011	CG7011	1.68	0.00
FBgn0039927	CG11155	CG11155	1.68	0.00
FBgn0020369	Pros45	Pros45	1.68	0.00
FBgn0031855	CG11221	CG11221	1.68	0.00
FBgn0052041	Hsp22	Heat shock protein 22	1.68	0.00
FBgn0030818	Arpc3B	Arpc3B	1.68	0.00
FBgn0028523	CG5888	CG5888	1.68	0.00
FBgn0026379	Pten	Pten	1.68	0.00
FBgn0025807	Rad9	Rad9	1.68	0.00
FBgn0036884	CG14098	CG14098	1.68	0.00
FBgn0016126	CaMKI	Calcium/calmodulin-dependent protein kinase I	1.68	0.01
FBgn0037332	CG14670	CG14670	1.68	0.00
FBgn0036906	CG14102	CG14102	1.68	0.00
FBgn0029512	Aos1	Aos1	1.68	0.00
FBgn0031977	CG7380	CG7380	1.67	0.00
FBgn0035902	CG6683	CG6683	1.67	0.00
FBgn0032117	FucTB	FucTB	1.67	0.00
FBgn0035020	CG13585	CG13585	1.67	0.00
FBgn0033005	CG3107	CG3107	1.67	0.00
FBgn0034199	CG15917	CG15917	1.67	0.00
FBgn0051918	CG31918	CG31918	1.67	0.00
FBgn0025458	Bub1	Bub1	1.67	0.01
FBgn0035790	Cyp316a1	Cyp316a1	1.67	0.00
FBgn0051998	CG31998	CG31998	1.67	0.00
FBgn0036016	CG3306	CG3306	1.67	0.00
FBgn0027783	SMC2	SMC2	1.67	0.00
FBgn0010607	l(2)05714	lethal (2) 05714	1.67	0.00
FBgn0035623	mthl2	methuselah-like 2	1.67	0.00
FBgn0020907	Scp2	Sarcoplasmic calcium-binding protein 2	1.67	0.00
FBgn0030114	CG17754	CG17754	1.67	0.00
FBgn0035213	CG2199	CG2199	1.67	0.00
FBgn0050268	CG30268	CG30268	1.67	0.00
FBgn0040078	pont	pontin	1.67	0.00
FBgn0038312	CG4334	CG4334	1.67	0.00
FBgn0035199	CG9134	CG9134	1.67	0.00
FBgn0033544	CG7220	CG7220	1.67	0.00

FBgn0030941	wgn	wengen	1.67	0.00
FBgn0029730	CG12681	CG12681	1.66	0.00
FBgn0003391	shg	shotgun	1.66	0.00
FBgn0033962	CG10153	CG10153	1.66	0.00
FBgn0038985	CG6921	CG6921	1.66	0.00
FBgn0036291	CG10681	CG10681	1.66	0.00
FBgn0036262	CG6910	CG6910	1.66	0.00
FBgn0013305	Nmda1	N-methyl-D-aspartate receptor-associated protein	1.66	0.00
FBgn0036522	CG7372	CG7372	1.66	0.00
FBgn0036207	CG10907	CG10907	1.66	0.00
FBgn0032683	CG10275	CG10275	1.66	0.00
FBgn0027339	jim	jim	1.66	0.00
FBgn0039413	CG14556	CG14556	1.66	0.00
FBgn0022984	qkr58E-3	quaking related 58E-3	1.66	0.00
FBgn0036723	CG12229	CG12229	1.66	0.00
FBgn0016076	vri	vrille	1.66	0.00
FBgn0037087	CG7519	CG7519	1.66	0.00
FBgn0037809	CG12818	CG12818	1.65	0.00
FBgn0030704	CG15916	CG15916	1.65	0.00
FBgn0035851	MED24	Mediator complex subunit 24	1.65	0.00
FBgn0037933	Ho	Heme oxygenase	1.65	0.00
FBgn0038102	CG14383	CG14383	1.65	0.00
FBgn0035975	PGRP-LA	Peptidoglycan recognition protein LA	1.65	0.00
FBgn0011296	l(2)efl	lethal (2) essential for life	1.65	0.00
FBgn0029945	CG18155	CG18155	1.65	0.00
FBgn0000216	Brd	Bearded	1.65	0.00
FBgn0014010	Rab5	Rab-protein 5	1.65	0.00
FBgn0032745	CG10473	CG10473	1.65	0.00
FBgn0037556	CG9636	CG9636	1.65	0.01
FBgn0031358	CG17657	CG17657	1.65	0.00
FBgn0036520	CG13449	CG13449	1.65	0.00
FBgn0034769	Obp58c	Odorant-binding protein 58c	1.65	0.00
FBgn0034833	CG13539	CG13539	1.65	0.00
FBgn0003444	smo	smoothened	1.65	0.00
FBgn0035538	DopEcR	DopEcR	1.65	0.00
FBgn0037799	CG12816	CG12816	1.65	0.00

FBgn0034795	MED23	Mediator complex subunit 23	1.65	0.00
FBgn0030756	CG9903	CG9903	1.65	0.00
FBgn0034737	CG11362	CG11362	1.65	0.00
FBgn0037434	CG1249	CG1249	1.65	0.00
FBgn0035640	mad2	mad2	1.65	0.00
FBgn0037696	CG9362	CG9362	1.65	0.00
FBgn0000536	eas	easily shocked	1.65	0.00
FBgn0033105	l(2)k03203	lethal (2) k03203	1.65	0.00
FBgn0014032	Sptr	Sepiapterin reductase	1.65	0.00
FBgn0005617	msl-1	male-specific lethal 1	1.65	0.00
FBgn0035989	CG3967	CG3967	1.65	0.00
FBgn0029996	Ubc-E2H	Ubc-E2H	1.64	0.01
FBgn0039132	AP-1sigma	AP-1sigma	1.64	0.00
FBgn0036153	CG7573	CG7573	1.64	0.00
FBgn0002733	HLHmbeta	E(spl) region transcript mbeta	1.64	0.00
FBgn0035969	CG4476	CG4476	1.64	0.00
FBgn0030813	CG4949	CG4949	1.64	0.00
FBgn0025676	CkIIalpha-i3	CKII-alpha subunit interactor-3	1.64	0.00
FBgn0011747	Ank	Ankyrin	1.64	0.00
FBgn0031205	CG12474	CG12474	1.64	0.00
FBgn0037171	CG14459	CG14459	1.64	0.00
FBgn0035581	CG17150	CG17150	1.64	0.00
FBgn0032036	CG13384	CG13384	1.64	0.00
FBgn0032250	CG5198	CG5198	1.64	0.00
FBgn0010039	GstD3	Glutathione S transferase D3	1.64	0.00
FBgn0035693	CG8219	CG8219	1.64	0.00
FBgn0036661	CG9705	CG9705	1.64	0.00
FBgn0040684	CG12589	CG12589	1.63	0.00
FBgn0052369	CG32369	CG32369	1.63	0.00
FBgn0051607	CG31607	CG31607	1.63	0.00
FBgn0036972	CG6434	CG6434	1.63	0.00
FBgn0035159	CG13896	CG13896	1.63	0.00
FBgn0036146	CG14141	CG14141	1.63	0.00
FBgn0004108	Nrt	Neurotactin	1.63	0.00
FBgn0032538	CG16885	CG16885	1.63	0.00
FBgn0031695	Cyp4ac3	Cyp4ac3	1.63	0.00

FBgn0023081	gek	genghis khan	1.63	0.00
FBgn0038296	CG6752	CG6752	1.63	0.00
FBgn0035702	CG10147	CG10147	1.63	0.00
FBgn0031504	CG15403	CG15403	1.63	0.00
FBgn0035891	Oseg1	Oseg1	1.63	0.00
FBgn0030590	CG9518	CG9518	1.63	0.00
FBgn0030627	gce	germ cell-expressed bHLH-PAS	1.63	0.00
FBgn0035296	CG11814	CG11814	1.63	0.00
FBgn0031996	CG8460	CG8460	1.63	0.00
FBgn0031458	aph-1	anterior pharynx defective 1	1.63	0.00
FBgn0039403	CG14549	CG14549	1.63	0.00
FBgn0015037	Cyp4p1	Cytochrome P450-4p1	1.63	0.00
FBgn0058283	CG17528	CG17528	1.63	0.00
FBgn0001112	Gld	Glucose dehydrogenase	1.63	0.00
FBgn0030673	CG15601	CG15601	1.63	0.00
FBgn0030178	CG2974	CG2974	1.63	0.00
FBgn0058010	cta	concertina	1.63	0.00
FBgn0005666	bt	bent	1.63	0.00
FBgn0019972	Ice	Ice	1.63	0.00
FBgn0030179	CG12094	CG12094	1.63	0.00
FBgn0014163	fax	failed axon connections	1.63	0.02
FBgn0003447	sn	singed	1.63	0.00
FBgn0038465	CG8913	CG8913	1.63	0.00
FBgn0039006	Cyp6d4	Cyp6d4	1.63	0.00
FBgn0025700	CG5885	CG5885	1.63	0.00
FBgn0030719	eIF5	eIF5	1.62	0.00
FBgn0031388	CG12674	CG12674	1.62	0.00
FBgn0029898	CG14439	CG14439	1.62	0.00
FBgn0036504	yellow-k	yellow-k	1.62	0.00
FBgn0035482	CG14985	CG14985	1.62	0.00
FBgn0036476	CG6945	CG6945	1.62	0.00
FBgn0025615	torp4a	torp4a	1.62	0.00
FBgn0035507	CG1308	CG1308	1.62	0.00
FBgn0015829	TfIIEbeta	Transcription factor IIEbeta	1.62	0.00
FBgn0011274	Dif	Dorsal-related immunity factor	1.62	0.00
FBgn0027542	CG6014	CG6014	1.62	0.00

FBgn0030525	CG10996	CG10996	1.62	0.00
FBgn0034512	CG18067	CG18067	1.62	0.00
FBgn0002778	mnd	minidiscs	1.62	0.00
FBgn0039144	Atg6	Autophagy-specific gene 6	1.62	0.00
FBgn0032420	CG6583	CG6583	1.62	0.00
FBgn0031283	CG15880	CG15880	1.62	0.00
FBgn0037279	CG1129	CG1129	1.62	0.00
FBgn0026063	KP78b	KP78b	1.62	0.00
FBgn0037368	CG1239	CG1239	1.62	0.00
FBgn0029094	asf1	anti-silencing factor 1	1.62	0.00
FBgn0038932	CG5740	CG5740	1.62	0.00
FBgn0033265	CG8710	CG8710	1.62	0.00
FBgn0031037	CG14207	CG14207	1.62	0.00
FBgn0030749	Anxb11	Annexin B11	1.62	0.00
FBgn0037722	CG8319	CG8319	1.62	0.01
FBgn0031762	CG9098	CG9098	1.62	0.00
FBgn0052944	CG32944	CG32944	1.62	0.00
FBgn0026148	CG12253	CG12253	1.62	0.00
FBgn0035194	CG9187	CG9187	1.62	0.00
FBgn0030276	Dlic2	Dlic2	1.62	0.00
FBgn0032030	CG17293	CG17293	1.61	0.00
FBgn0031513	CG3347	CG3347	1.61	0.00
FBgn0004395	unk	unkempt	1.61	0.00
FBgn0022701	Cht3	Chitinase 3	1.61	0.00
FBgn0035407	CG14962	CG14962	1.61	0.00
FBgn0037647	CG11968	CG11968	1.61	0.00
FBgn0038210	CG3199	CG3199	1.61	0.00
FBgn0030462	CG15742	CG15742	1.61	0.00
FBgn0058289	CG17883	CG17883	1.61	0.00
FBgn0025637	skpA	skpA	1.61	0.00
FBgn0033775	Cyp9h1	Cyp9h1	1.61	0.00
FBgn0031136	l(1)19Ec	lethal (1) 19Ec	1.61	0.00
FBgn0039328	CG10675	CG10675	1.61	0.00
FBgn0038419	CG14879	CG14879	1.61	0.00
FBgn0035514	CG1332	CG1332	1.61	0.00
FBgn0037234	CG9795	CG9795	1.61	0.00

FBgn0001227	Hsp67Ba	Heat shock gene 67Ba	1.61	0.00
FBgn0001090	bnb	bangles and beads	1.61	0.00
FBgn0039914	mav	maverick	1.61	0.05
FBgn0033675	CG8889	CG8889	1.61	0.00
FBgn0035763	CG8602	CG8602	1.61	0.00
FBgn0028857	CG12448	CG12448	1.61	0.00
FBgn0031270	CG13689	CG13689	1.61	0.00
FBgn0031969	CG7228	CG7228	1.61	0.00
FBgn0031921	CG6630	CG6630	1.61	0.00
FBgn0032728	CG18398	CG18398	1.61	0.00
FBgn0011653	mas	masquerade	1.61	0.00
FBgn0032522	CG16848	CG16848	1.61	0.00
FBgn0034228	CG14479	CG14479	1.61	0.00
FBgn0031279	CG3544	CG3544	1.61	0.00
FBgn0039874	CG2135	CG2135	1.61	0.00
FBgn0035160	CG13897	CG13897	1.61	0.00
FBgn0039735	CG7911	CG7911	1.61	0.00
FBgn0016797	fz2	frizzled 2	1.61	0.00
FBgn0033788	CG13323	CG13323	1.61	0.00
FBgn0035161	CG13898	CG13898	1.61	0.00
FBgn0032074	Tsp29Fa	Tetraspanin 29Fa	1.61	0.00
FBgn0039088	CG10164	CG10164	1.61	0.00
FBgn0039165	CG6204	CG6204	1.60	0.00
FBgn0032634	Rpb11	Rpb11	1.60	0.00
FBgn0039002	CG17625	CG17625	1.60	0.00
FBgn0010314	Cks30A	Cyclin-dependent kinase subunit 30A	1.60	0.00
FBgn0051169	CG31169	CG31169	1.60	0.00
FBgn0038432	CG14883	CG14883	1.60	0.00
FBgn0040871	CG12479	CG12479	1.60	0.00
FBgn0051285	CG31285	CG31285	1.60	0.00
FBgn0052176	CG32176	CG32176	1.60	0.00
FBgn0030393	CG15926	CG15926	1.60	0.00
FBgn0031816	CG16947	CG16947	1.60	0.00
FBgn0035574	Gef64C	Guanine nucleotide exchange factor GEF64C	1.60	0.00
FBgn0035090	CG2736	CG2736	1.60	0.00
FBgn0036317	CG10948	CG10948	1.60	0.00

FBgn0040493	granny-smith	granny smith	1.60	0.00
FBgn0017581	Lk6	Lk6	1.60	0.00
FBgn0035111	CG16940	CG16940	1.60	0.00
FBgn0032047	CG13088	CG13088	1.60	0.00
FBgn0004429	LysP	Lysozyme P	1.60	0.00
FBgn0011241	cbx	crossbronx	1.60	0.00
FBgn0028527	CG18507	CG18507	1.60	0.00
FBgn0014020	Rho1	Rho1	1.60	0.00
FBgn0039405	CG14550	CG14550	1.60	0.00
FBgn0010660	Nup214	Nup214	1.60	0.00
FBgn0030864	CG8173	CG8173	1.60	0.00
FBgn0036426	CG9592	CG9592	1.60	0.00
FBgn0031049	CG14214	CG14214	1.60	0.00
FBgn0028486	CG2967	CG2967	1.60	0.00
FBgn0004797	mdy	midway	1.60	0.00
FBgn0036332	CG11261	CG11261	1.60	0.00
FBgn0038965	mats	mob as tumor suppressor	1.60	0.00
FBgn0051368	CG31368	CG31368	1.60	0.00
FBgn0031610	CG15436	CG15436	1.60	0.00
FBgn0017418	ari-1	ariadne	1.60	0.00
FBgn0031973	CG7219	CG7219	1.60	0.00
FBgn0039228	CG6980	CG6980	1.60	0.00
FBgn0033993	CG8089	CG8089	1.60	0.00
FBgn0013301	Mst35Bb	Male-specific-transcript-35Bb	1.60	0.00
FBgn0016070	smg	smaug	1.60	0.00
FBgn0028855	CG15282	CG15282	1.60	0.00
FBgn0011802	Dhh1	DEAD/DEAH RNA helicase 1	1.59	0.00
FBgn0030725	CG8958	CG8958	1.59	0.00
FBgn0045495	Gr28b	Gustatory receptor 28b	1.59	0.00
FBgn0020622	Pi3K21B	Pi3K21B	1.59	0.00
FBgn0035627	Sse	Separase	1.59	0.00
FBgn0051716	CG31716	CG31716	1.59	0.00
FBgn0032658	CG5681	CG5681	1.59	0.00
FBgn0035986	CG4022	CG4022	1.59	0.00
FBgn0058291	CG17486	CG17486	1.59	0.00
FBgn0039129	RpS19b	Ribosomal protein S19b	1.59	0.00

FBgn0030485	CG1998	CG1998	1.59	0.00
FBgn0035970	CG4483	CG4483	1.59	0.00
FBgn0014857	His3.3A	Histone H3.3A	1.59	0.00
FBgn0035772	Sh3beta	Sh3beta	1.59	0.00
FBgn0052542	CG32542	CG32542	1.59	0.00
FBgn0052499	CG32499	CG32499	1.59	0.00
FBgn0034739	CG3927	CG3927	1.59	0.00
FBgn0032700	CG10338	CG10338	1.59	0.00
FBgn0037963	Cad87A	Cad87A	1.59	0.00
FBgn0030342	CG10347	CG10347	1.59	0.00
FBgn0033029	l(2)NC136	lethal (2) NC136	1.59	0.01
FBgn0030456	CG4332	CG4332	1.59	0.00
FBgn0035415	CG14966	CG14966	1.59	0.00
FBgn0033261	CG18316	CG18316	1.59	0.00
FBgn0001149	GstD1	Glutathione S transferase D1	1.59	0.02
FBgn0037252	CG14650	CG14650	1.59	0.00
FBgn0039907	lgs	legless	1.59	0.00
FBgn0038090	CG10909	CG10909	1.59	0.00
FBgn0035444	CG12012	CG12012	1.59	0.01
FBgn0031315	CG14341	CG14341	1.59	0.00
FBgn0005278	M(2)21AB	Minute (2) 21AB	1.59	0.00
FBgn0033453	CG1667	CG1667	1.58	0.00
FBgn0033285	CG18449	CG18449	1.58	0.02
FBgn0039356	CG5039	CG5039	1.58	0.00
FBgn0038979	CG7046	CG7046	1.58	0.00
FBgn0010633	vlc	vulcan	1.58	0.00
FBgn0011586	e(r)	enhancer of rudimentary	1.58	0.01
FBgn0037737	Pnn	Pinin	1.58	0.00
FBgn0030591	CG9517	CG9517	1.58	0.02
FBgn0036218	CG14128	CG14128	1.58	0.00
FBgn0035094	CG9380	CG9380	1.58	0.00
FBgn0033996	CG11807	CG11807	1.58	0.00
FBgn0037514	CG10919	CG10919	1.58	0.00
FBgn0031995	CG8475	CG8475	1.58	0.00
FBgn0043455	CG5986	CG5986	1.58	0.00
FBgn0015380	drl	derailed	1.58	0.00

FBgn0033551	CG7222	CG7222	1.58	0.01
FBgn0038082	CG5724	CG5724	1.58	0.00
FBgn0039505	CG5934	CG5934	1.58	0.00
FBgn0030320	CG2247	CG2247	1.58	0.00
FBgn0001085	fz	frizzled	1.58	0.00
FBgn0030170	CG2990	CG2990	1.58	0.00
FBgn0004381	Klp68D	Kinesin-like protein at 68D	1.58	0.00
FBgn0011305	Rsf1	Repressor splicing factor 1	1.58	0.00
FBgn0045064	bwa	brain washing	1.58	0.00
FBgn0039902	CG2177	CG2177	1.58	0.00
FBgn0035631	Txl	Thioredoxin-like	1.58	0.00
FBgn0036223	CG14126	CG14126	1.58	0.00
FBgn0014184	Oda	Ornithine decarboxylase antizyme	1.58	0.00
FBgn0033287	CG8701	CG8701	1.58	0.02
FBgn0022361	Pur-alpha	Purine-rich binding protein-alpha	1.57	0.00
FBgn0004556	Dbp73D	Dead box protein 73D	1.57	0.00
FBgn0027617	CG5808	CG5808	1.57	0.00
FBgn0052089	Vha16-2	Vha16-2	1.57	0.00
FBgn0005386	ash1	absent, small, or homeotic discs 1	1.57	0.00
FBgn0039574	CG16918	CG16918	1.57	0.00
FBgn0028519	CG4500	CG4500	1.57	0.00
FBgn0003087	pim	pimples	1.57	0.00
FBgn0025394	CG32810	CG32810	1.57	0.00
FBgn0033613	CG13211	CG13211	1.57	0.00
FBgn0036708	CG13725	CG13725	1.57	0.00
FBgn0033755	CG8594	CG8594	1.57	0.00
FBgn0037223	CG14638	CG14638	1.57	0.00
FBgn0032588	CG5968	CG5968	1.57	0.00
FBgn0029952	CG12689	CG12689	1.57	0.00
FBgn0030700	CG15914	CG15914	1.57	0.00
FBgn0033970	CG10205	CG10205	1.57	0.00
FBgn0015586	Acp76A	Accessory gland-specific peptide 76A	1.57	0.00
FBgn0036331	CG14117	CG14117	1.57	0.00
FBgn0032231	CG5056	CG5056	1.57	0.00
FBgn0010387	Dbi	Diazepam-binding inhibitor	1.57	0.00
FBgn0037668	CG16736	CG16736	1.57	0.00

FBgn0036812	Nufip	Nufip	1.57	0.00
FBgn0053100	4EHP	4EHP	1.57	0.00
FBgn0033112	CG9454	CG9454	1.57	0.00
FBgn0030757	CG9902	CG9902	1.57	0.00
FBgn0052048	CG32048	CG32048	1.57	0.00
FBgn0036141	MRP	Multidrug-Resistance like Protein 1	1.57	0.00
FBgn0037417	Osi10	Osiris 10	1.57	0.00
FBgn0035703	CG8270	CG8270	1.57	0.00
FBgn0037967	CG3281	CG3281	1.57	0.00
FBgn0037578	CG9601	CG9601	1.56	0.00
FBgn0030942	l(1)G0003	lethal (1) G0003	1.56	0.00
FBgn0036793	CG4174	CG4174	1.56	0.00
FBgn0036263	thoc6	thoc6	1.56	0.00
FBgn0052253	CG11583	CG11583	1.56	0.00
FBgn0036118	CG14145	CG14145	1.56	0.00
FBgn0035892	exo70	exo70	1.56	0.00
FBgn0034774	CG13526	CG13526	1.56	0.00
FBgn0031213	galectin	galectin	1.56	0.00
FBgn0053188	CG33936	CG33936	1.56	0.00
FBgn0037553	CG18249	CG18249	1.56	0.00
FBgn0030624	CG9106	CG9106	1.56	0.00
FBgn0015287	RfC40	Replication-factor-C 40kD subunit	1.56	0.00
FBgn0029752	TrxT	Thioredoxin T	1.56	0.00
FBgn0033639	CG9003	CG9003	1.56	0.00
FBgn0037433	CG17919	CG17919	1.56	0.00
FBgn0036406	CG13484	CG13484	1.56	0.00
FBgn0037228	CG1092	CG1092	1.56	0.00
FBgn0052179	Krn	Keren	1.56	0.00
FBgn0031463	CG15400	CG15400	1.56	0.00
FBgn0051217	CG31217	CG31217	1.56	0.00
FBgn0010287	Trf	TBP-related factor	1.56	0.00
FBgn0040786	CG14104	CG14104	1.56	0.00
FBgn0052043	CG32043	CG32043	1.56	0.00
FBgn0031876	CG9200	CG9200	1.56	0.00
FBgn0033724	CG8501	CG8501	1.56	0.00
FBgn0026315	Ugt35a	UDP-glycosyltransferase 35a	1.56	0.00

FBgn0000635	Fas2	Fasciclin 2	1.56	0.01
FBgn0036668	CG9715	CG9715	1.56	0.00
FBgn0014189	Hel25E	Helicase at 25E	1.56	0.00
FBgn0038451	CG14893	CG14893	1.56	0.00
FBgn0038819	CG5494	CG5494	1.56	0.05
FBgn0034651	CG15676	CG15676	1.56	0.00
FBgn0026141	Cdlc2	Cytoplasmic dynein light chain 2	1.56	0.00
FBgn0028997	nmdyn-D7	nmdyn-D7	1.56	0.00
FBgn0016701	Rab4	Rab-protein 4	1.56	0.00
FBgn0030079	CG7267	CG7267	1.56	0.00
FBgn0035843	mus301	mutagen-sensitive 301	1.56	0.00
FBgn0015754	Lis-1	Lissencephaly-1	1.56	0.00
FBgn0015331	abs	abstrakt	1.56	0.00
FBgn0028689	Rpn6	Proteasome p44.5 subunit	1.56	0.00
FBgn0035907	CG6662	CG6662	1.56	0.00
FBgn0034847	CG3502	CG3502	1.56	0.00
FBgn0039869	CG1890	CG1890	1.55	0.00
FBgn0010198	RpS15Aa	Ribosomal protein S15Aa	1.55	0.00
FBgn0033059	CG7845	CG7845	1.55	0.00
FBgn0033450	CG12924	CG12924	1.55	0.00
FBgn0003495	spz	spatzle	1.55	0.00
FBgn0010405	Pros25	Proteasome 25kD subunit	1.55	0.00
FBgn0011591	fng	fringe	1.55	0.00
FBgn0058460	CG17159	CG17159	1.55	0.00
FBgn0011710	38961	Septin-1	1.55	0.01
FBgn0040942	CG12643	CG12643	1.55	0.00
FBgn0030025	CG2147	CG2147	1.55	0.00
FBgn0058411	Parp	Poly-(ADP-ribose) polymerase	1.55	0.01
FBgn0033017	CG10465	CG10465	1.55	0.00
FBgn0034760	CG13512	CG13512	1.55	0.00
FBgn0030801	CG4875	CG4875	1.55	0.00
FBgn0040687	CG14645	CG14645	1.55	0.00
FBgn0053091	CG11889	CG11889	1.55	0.00
FBgn0004174	Mst84Dc	Male-specific RNA 84Dc	1.55	0.01
FBgn0026374	Rhp	Rhophilin	1.55	0.00
FBgn0000426	DebB	Developmental embryonic B	1.55	0.00

FBgn0032115	CG4438	CG4438	1.55	0.00
FBgn0032202	CG18619	CG18619	1.55	0.00
FBgn0036277	CG10418	CG10418	1.55	0.00
FBgn0036156	CG11726	CG11726	1.55	0.00
FBgn0037634	CG8359	CG8359	1.55	0.00
FBgn0010460	bun	bunched	1.55	0.00
FBgn0035761	RhoGEF4	RhoGEF4	1.55	0.00
FBgn0029984	l(1)G0193	lethal (1) G0193	1.55	0.00
FBgn0030409	CG15728	CG15728	1.55	0.00
FBgn0015831	Rtnl2	Rtnl2	1.55	0.00
FBgn0031878	sip2	septin interacting protein 2	1.54	0.00
FBgn0004173	Mst84Db	Male-specific RNA 84Db	1.54	0.01
FBgn0030765	CG9742	CG9742	1.54	0.00
FBgn0027885	Aac11	Aac11	1.54	0.00
FBgn0001185	her	hermaphrodite	1.54	0.00
FBgn0039122	CG10192	CG10192	1.54	0.00
FBgn0032497	CG6043	CG6043	1.54	0.00
FBgn0030936	CG6481	CG6481	1.54	0.00
FBgn0032665	CG15152	CG15152	1.54	0.00
FBgn0035782	CG14835	CG14835	1.54	0.00
FBgn0053038	CG10731	CG10731	1.54	0.00
FBgn0001981	esg	escargot	1.54	0.00
FBgn0035737	CG8634	CG8634	1.54	0.00
FBgn0003261	Rm62	Rm62	1.54	0.00
FBgn0052662	CG32662	CG32662	1.54	0.00
FBgn0028515	CG4930	CG4930	1.54	0.00
FBgn0026737	CG6171	CG6171	1.54	0.00
FBgn0034654	CG10306	CG10306	1.54	0.00
FBgn0035464	CG12006	CG12006	1.54	0.00
FBgn0034468	Obp56a	Odorant-binding protein 56a	1.54	0.00
FBgn0001624	dlg1	discs large 1	1.54	0.00
FBgn0036421	CG13481	CG13481	1.54	0.00
FBgn0003449	snf	sans fille	1.54	0.00
FBgn0004449	Ten-m	Tenascin major	1.54	0.00
FBgn0033063	CG14589	CG14589	1.54	0.00
FBgn0020294	ko	knockout	1.54	0.00

FBgn0039462	CG14252	CG14252	1.54	0.00
FBgn0039323	CG10559	CG10559	1.54	0.00
FBgn0036313	Atg1	Autophagy-specific gene 1	1.54	0.00
FBgn0013343	Syx1A	Syntaxin 1A	1.54	0.00
FBgn0038071	CG6234	CG6234	1.54	0.00
FBgn0037076	CG12972	CG12972	1.54	0.00
FBgn0035917	CG6416	CG6416	1.54	0.00
FBgn0052912	PGRP-LD	Peptidoglycan recognition protein LD	1.54	0.01
FBgn0036478	CG6854	CG6854	1.54	0.00
FBgn0030457	CG12096	CG12096	1.53	0.00
FBgn0032218	CG5381	CG5381	1.53	0.00
FBgn0052086	CG32086	CG32086	1.53	0.00
FBgn0027496	CG9543	CG9543	1.53	0.00
FBgn0037017	CG4074	CG4074	1.53	0.00
FBgn0004919	gol	goliath	1.53	0.01
FBgn0034366	Atg7	Autophagy-specific gene 7	1.53	0.00
FBgn0001977	l(2)35Bg	lethal (2) 35Bg	1.53	0.00
FBgn0030539	CG1368	CG1368	1.53	0.01
FBgn0035709	CG10124	CG10124	1.53	0.00
FBgn0028387	chm	chameau	1.53	0.00
FBgn0035617	CG5146	CG5146	1.53	0.00
FBgn0034230	CG4853	CG4853	1.53	0.00
FBgn0032702	CG10376	CG10376	1.53	0.00
FBgn0020429	GluRIIB	Glutamate receptor IIB	1.53	0.00
FBgn0038368	CG14876	CG14876	1.53	0.00
FBgn0000338	cnc	cap-n-collar	1.53	0.00
FBgn0027948	msps	mini spindles	1.53	0.00
FBgn0000273	Pka-C1	cAMP-dependent protein kinase 1	1.53	0.00
FBgn0036441	CG13476	CG13476	1.53	0.00
FBgn0036090	CG8009	CG8009	1.53	0.00
FBgn0037396	CG11459	CG11459	1.53	0.01
FBgn0002022	Catsup	Catecholamines up	1.53	0.00
FBgn0027565	CG5498	CG5498	1.53	0.00
FBgn0024252	Ark	Apaf-1-related-killer	1.53	0.00
FBgn0036446	CG9384	CG9384	1.53	0.00
FBgn0039421	CG6036	CG6036	1.53	0.00

FBgn0004586	grh	grainy head	1.53	0.00
FBgn0036125	CG6279	CG6279	1.53	0.00
FBgn0029114	Tollo	Tollo	1.52	0.00
FBgn0039415	CG6142	CG6142	1.52	0.00
FBgn0037094	CG7611	CG7611	1.52	0.00
FBgn0020414	Idgf3	Imaginal disc growth factor 3	1.52	0.00
FBgn0040285	Scamp	Scamp	1.52	0.00
FBgn0001084	fy	fuzzy	1.52	0.00
FBgn0030235	Imp	IGF-II mRNA-binding protein	1.52	0.00
FBgn0032929	Mcm10	Sensitized chromosome inheritance modifier 19	1.52	0.00
FBgn0033187	CG2144	CG2144	1.52	0.00
FBgn0035480	CG14984	CG14984	1.52	0.00
FBgn0028433	Ggamma30A	G protein gamma30A	1.52	0.00
FBgn0030646	CG9203	CG9203	1.52	0.00
FBgn0040932	CG12650	CG12650	1.52	0.00
FBgn0030952	CG12609	CG12609	1.52	0.00
FBgn0035211	CG2211	CG2211	1.52	0.00
FBgn0004370	Ptp10D	Protein tyrosine phosphatase 10D	1.52	0.00
FBgn0003716	tkv	thickveins	1.52	0.00
FBgn0052475	mthl8	methuselah-like 8	1.52	0.01
FBgn0034785	CG3649	CG3649	1.52	0.00
FBgn0040963	CG18662	CG18662	1.52	0.00
FBgn0032943	Tsp39D	Tetraspanin 39D	1.52	0.00
FBgn0030803	CG4880	CG4880	1.52	0.00
FBgn0034246	Dcr-2	Dicer-2	1.52	0.00
FBgn0051802	CG31802	CG31802	1.52	0.00
FBgn0050169	CG30169	CG30169	1.52	0.00
FBgn0000412	D1	D1 chromosomal protein	1.52	0.00
FBgn0032553	CG4480	CG4480	1.52	0.00
FBgn0036641	Smn	survival motor neuron	1.52	0.00
FBgn0038192	CG9924	CG9924	1.52	0.00
FBgn0001122	G-oalpha47A	G protein oalpha 47A	1.52	0.01
FBgn0027049	DDB1	DDB1	1.52	0.00
FBgn0032935	CG8678	CG8678	1.52	0.00
FBgn0034032	CG8195	CG8195	1.52	0.00
FBgn0000253	Cam	Calmodulin	1.52	0.01

FBgn0032859	Arc-p34	Arc-p34	1.52	0.00
FBgn0003276	RpII140	RNA polymerase II 140kD subunit	1.52	0.00
FBgn0029878	Pat1	Pat1	1.52	0.00
FBgn0036814	CG14073	CG14073	1.52	0.00
FBgn0003731	Egfr	Epidermal growth factor receptor	1.52	0.01
FBgn0035968	CG4484	CG4484	1.52	0.00
FBgn0005630	lola	longitudinals lacking	1.52	0.00
FBgn0035030	pio	piopio	1.51	0.01
FBgn0020386	Pk61C	Protein kinase 61C	1.51	0.00
FBgn0030623	CG15031	CG15031	1.51	0.00
FBgn0010300	brat	brain tumor	1.51	0.00
FBgn0001255	ImpE3	Ecdysone-inducible gene E3	1.51	0.00
FBgn0035647	CG10486	CG10486	1.51	0.00
FBgn0037652	CG11980	CG11980	1.51	0.00
FBgn0053178	CG33178	CG33178	1.51	0.00
FBgn0037620	CG9793	CG9793	1.51	0.00
FBgn0032901	CG9339	CG9339	1.51	0.00
FBgn0035675	CG6610	CG6610	1.51	0.00
FBgn0036000	CG3434	CG3434	1.51	0.00
FBgn0039277	CG13650	CG13650	1.51	0.00
FBgn0004401	Pep	Protein on ecdysone puffs	1.51	0.00
FBgn0031043	CG14222	CG14222	1.51	0.00
FBgn0032752	CG10702	CG10702	1.51	0.00
FBgn0036438	CG13477	CG13477	1.51	0.01
FBgn0026575	hang	hangover	1.51	0.00
FBgn0033963	CG12857	CG12857	1.51	0.00
FBgn0035808	SP2523	SP2523	1.51	0.00
FBgn0039338	XNP	XNP	1.51	0.00
FBgn0033676	CG17509	CG17509	1.51	0.00
FBgn0034933	CG3735	CG3735	1.51	0.00
FBgn0036707	CG13724	CG13724	1.51	0.00
FBgn0034914	CG5554	CG5554	1.51	0.00
FBgn0031875	CG3430	CG3430	1.51	0.00
FBgn0030739	CG13957	CG13957	1.51	0.00
FBgn0030701	CG16952	CG16952	1.51	0.00
FBgn0033968	CG10200	CG10200	1.51	0.00

FBgn0032016	CG7818	CG7818	1.51	0.00
FBgn0015602	BEAF-32	Boundary element-associated factor of 32kD	1.51	0.00
FBgn0036772	CG5290	CG5290	1.51	0.01
FBgn0039577	CG12516	CG12516	1.51	0.00
FBgn0037307	Tim17a2	Tim17a2	1.51	0.00
FBgn0005672	spi	spitz	1.51	0.00
FBgn0023417	AP-2	AP-2	1.51	0.00
FBgn0032002	CG8353	CG8353	1.51	0.00
FBgn0038209	CG9722	CG9722	1.51	0.00
FBgn0031284	CG3876	CG3876	1.50	0.00
FBgn0033081	geminin	geminin	1.50	0.00
FBgn0027936	vih	vihar	1.50	0.00
FBgn0031623	Taf12L	TBP-associated factor 30kD subunit alpha-2	1.50	0.00
FBgn0031023	CG14200	CG14200	1.50	0.00
FBgn0034985	CG3328	CG3328	1.50	0.00
FBgn0035812	CG7457	CG7457	1.50	0.00
FBgn0029959	Rab39	Rab39	1.50	0.00
FBgn0036443	CG13471	CG13471	1.50	0.00
FBgn0036927	CG7433	CG7433	1.50	0.00
FBgn0036580	PDCD-5	PDCD-5	1.50	0.00
FBgn0043903	dome	domeless	1.50	0.00
FBgn0034713	CG11291	CG11291	1.50	0.00
FBgn0038476	kuk	kugelkern	1.50	0.01
FBgn0030384	CG2577	CG2577	1.50	0.00
FBgn0036785	CG13700	CG13700	1.50	0.00
FBgn0040938	CG33557	CG33557	-1.50	0.00
FBgn0032310	CG17086	CG17086	-1.50	0.00
FBgn0021847	l(2)k14710	lethal (2) k14710	-1.50	0.00
FBgn0038467	CG3590	CG3590	-1.50	0.00
FBgn0035511	CG15007	CG15007	-1.50	0.00
FBgn0030665	CG15646	CG15646	-1.50	0.00
FBgn0010591	Sply	Sphingosine-1-phosphate lyase	-1.50	0.00
FBgn0000116	Argk	Arginine kinase	-1.50	0.00
FBgn0029862	CG12219	CG12219	-1.50	0.00
FBgn0053179	beat-IIIb	beat-IIIb	-1.50	0.05
FBgn0031036	CG14220	CG14220	-1.50	0.00

FBgn0039702	Vps16B	Vps16B	-1.50	0.00
FBgn0035344	Cyp4d20	Cyp4d20	-1.50	0.00
FBgn0037116	CG7158	CG7158	-1.50	0.00
FBgn0033108	CG15236	CG15236	-1.51	0.00
FBgn0034144	CG5089	CG5089	-1.51	0.00
FBgn0030035	CG11190	CG11190	-1.51	0.00
FBgn0050410	CG30410	CG30410	-1.51	0.00
FBgn0035938	orb2	orb2	-1.51	0.00
FBgn0052056	CG32056	CG32056	-1.51	0.00
FBgn0051037	ca	claret	-1.51	0.00
FBgn0052521	CG32521	CG32521	-1.51	0.00
FBgn0051956	pgant4	polypeptide GalNAc transferase 4	-1.51	0.00
FBgn0036422	CG3868	CG3868	-1.51	0.00
FBgn0040823	dpr6	dpr6	-1.51	0.00
FBgn0030359	CG18130	CG18130	-1.51	0.00
FBgn0022935	D19A	D19A	-1.51	0.00
FBgn0035530	dyl	dusky-like	-1.51	0.00
FBgn0037070	CG11309	CG11309	-1.51	0.00
FBgn0052737	CG33691	CG33691	-1.51	0.00
FBgn0031100	CG1504	CG1504	-1.51	0.00
FBgn0040388	CG32796	CG32796	-1.51	0.00
FBgn0032616	CG31781	CG31781	-1.51	0.00
FBgn0034455	CG11007	CG11007	-1.51	0.00
FBgn0034052	CG8299	CG8299	-1.51	0.00
FBgn0034731	CG10384	CG10384	-1.51	0.00
FBgn0020642	Lcp65Ac	Lcp65Ac	-1.51	0.00
FBgn0034431	Tab2	Tab2	-1.51	0.00
FBgn0034496	CG9143	CG9143	-1.51	0.00
FBgn0038042	Scgbeta	Sarcoglycan beta	-1.51	0.00
FBgn0034883	CG17664	CG17664	-1.51	0.00
FBgn0030769	CG13012	CG13012	-1.51	0.00
FBgn0052206	CG32206	CG32206	-1.51	0.01
FBgn0029858	CG15896	CG15896	-1.51	0.00
FBgn0028920	CG8997	CG8997	-1.51	0.00
FBgn0032504	CG16970	CG16970	-1.51	0.00
FBgn0032143	CG4017	CG4017	-1.51	0.00

FBgn0001145	Gs2	Glutamine synthetase 2	-1.51	0.02
FBgn0027544	CG2217	CG2217	-1.52	0.00
FBgn0034085	Ptp52F	Ptp52F	-1.52	0.00
FBgn0038039	CG5196	CG5196	-1.52	0.00
FBgn0050174	CG30174	CG30174	-1.52	0.00
FBgn0036017	CG3280	CG3280	-1.52	0.01
FBgn0051676	CG31676	CG31676	-1.52	0.00
FBgn0033377	Pmm45A	Phosphomannomutase 45A	-1.52	0.00
FBgn0028687	Rpt1	Rpt1	-1.52	0.00
FBgn0029605	CG18082	CG18082	-1.52	0.01
FBgn0036557	mRpS31	mitochondrial ribosomal protein S31	-1.52	0.00
FBgn0030011	Gbeta5	Gbeta5	-1.52	0.00
FBgn0032720	mRpL13	mitochondrial ribosomal protein L13	-1.52	0.00
FBgn0015036	Cyp4ae1	Cytochrome P450-4ae1	-1.52	0.00
FBgn0031132	CG15450	CG15450	-1.52	0.00
FBgn0028388	capt	capulet	-1.52	0.00
FBgn0034858	eIF2B-delta	eIF2B-delta	-1.52	0.00
FBgn0031850	Tsp	Thrombospondin	-1.52	0.00
FBgn0034129	CG15925	CG15925	-1.52	0.00
FBgn0039391	CG8934	CG8934	-1.52	0.00
FBgn0035059	CG3894	CG3894	-1.52	0.00
FBgn0034876	CG3957	CG3957	-1.52	0.00
FBgn0025698	CG5594	CG5594	-1.52	0.00
FBgn0033166	CG11166	CG11166	-1.52	0.00
FBgn0037375	CG1193	CG1193	-1.52	0.00
FBgn0035374	mRpS35	mitochondrial ribosomal protein S35	-1.52	0.00
FBgn0040889	CG33253	CG33253	-1.53	0.00
FBgn0038017	CG4115	CG4115	-1.53	0.00
FBgn0031782	CG9226	CG9226	-1.53	0.00
FBgn0039626	CG1420	CG1420	-1.53	0.00
FBgn0010786	l(3)02640	lethal (3) 02640	-1.53	0.00
FBgn0033501	CG12911	CG12911	-1.53	0.00
FBgn0024362	CG11412	CG11412	-1.53	0.00
FBgn0034132	CG4439	CG4439	-1.53	0.01
FBgn0040553	CG14374	CG14374	-1.53	0.01
FBgn0004915	TfIIB	Transcription factor IIB	-1.53	0.00

FBgn0051708	CG31708	CG31708	-1.53	0.00
FBgn0042187	CG17234	CG17234	-1.53	0.00
FBgn0030540	CG11581	CG11581	-1.53	0.00
FBgn0035979	CG4446	CG4446	-1.53	0.00
FBgn0031836	CG11050	CG11050	-1.53	0.00
FBgn0036143	CG14142	CG14142	-1.53	0.02
FBgn0026619	tafazzin	tafazzin	-1.53	0.00
FBgn0030892	CG7206	CG7206	-1.53	0.00
FBgn0037607	CG8036	CG8036	-1.53	0.00
FBgn0000077	amx	almondex	-1.53	0.00
FBgn0001170	H2.0	Homeodomain protein 2.0	-1.53	0.00
FBgn0037428	Osi18	Osiris 18	-1.53	0.02
FBgn0010830	l(3)04053	lethal (3) 04053	-1.53	0.00
FBgn0016672	Ipp	Inositol polyphosphate 1-phosphatase	-1.53	0.00
FBgn0039156	CG6178	CG6178	-1.53	0.00
FBgn0027550	CG6495	CG6495	-1.53	0.00
FBgn0003651	svp	seven up	-1.53	0.00
FBgn0035348	CG16758	CG16758	-1.53	0.00
FBgn0030543	CG11585	CG11585	-1.53	0.00
FBgn0032663	CG15153	CG15153	-1.53	0.00
FBgn0030826	CG12995	CG12995	-1.53	0.00
FBgn0037563	CG11672	CG11672	-1.53	0.00
FBgn0035358	CG14949	CG14949	-1.54	0.00
FBgn0019643	Dat	Dopamine N acetyltransferase	-1.54	0.00
FBgn0039741	CG7943	CG7943	-1.54	0.00
FBgn0002622	RpS3	Ribosomal protein S3	-1.54	0.00
FBgn0031930	CG7025	CG7025	-1.54	0.00
FBgn0030610	CG9065	CG9065	-1.54	0.00
FBgn0052940	Pif1A	PFTAIRE-interacting factor 1A	-1.54	0.02
FBgn0037122	CG14570	CG14570	-1.54	0.00
FBgn0033659	Damm	Death associated molecule related to Mch2	-1.54	0.00
FBgn0030901	CG33639	CG33639	-1.54	0.00
FBgn0040393	CG14265	CG14265	-1.54	0.00
FBgn0032006	Pvr	PDGF- and VEGF-receptor related	-1.54	0.00
FBgn0035856	CG13679	CG13679	-1.54	0.00
FBgn0001205	Hmgcr	HMG Coenzyme A reductase	-1.54	0.00

FBgn0035035	CG3570	CG3570	-1.54	0.00
FBgn0036873	CG18294	CG18294	-1.54	0.00
FBgn0037687	CG8132	CG8132	-1.54	0.00
FBgn0023519	mRpL16	mitochondrial ribosomal protein L16	-1.54	0.00
FBgn0040548	dpr4	dpr4	-1.54	0.00
FBgn0030433	mRpL49	mitochondrial ribosomal protein L49	-1.54	0.00
FBgn0030991	CG7453	CG7453	-1.55	0.00
FBgn0031637	CG2950	CG2950	-1.55	0.00
FBgn0031436	CG3214	CG3214	-1.55	0.00
FBgn0034646	Rae1	Rae1	-1.55	0.00
FBgn0051326	CG31326	CG31326	-1.55	0.00
FBgn0027073	CG4302	CG4302	-1.55	0.00
FBgn0039363	LpR1	LpR1	-1.55	0.00
FBgn0004374	neb	nebbish	-1.55	0.00
FBgn0024947	NTPase	NTPase	-1.55	0.00
FBgn0033917	CG8503	CG8503	-1.55	0.00
FBgn0031688	Cyp28d2	Cyp28d2	-1.55	0.00
FBgn0030552	mRpL38	mitochondrial ribosomal protein L38	-1.55	0.00
FBgn0034938	CG3803	CG3803	-1.55	0.00
FBgn0015324	Vha26	Vacuolar H[+]-ATPase 26kD E subunit	-1.55	0.00
FBgn0026430	Grip84	gamma-tubulin ring protein 84	-1.55	0.00
FBgn0051634	Oatp26F	Organic anion transporting polypeptide 26F	-1.55	0.00
FBgn0034697	GM130	GM130	-1.55	0.00
FBgn0031357	mRpL48	mitochondrial ribosomal protein L48	-1.55	0.00
FBgn0039694	fig	fos intronic gene	-1.55	0.00
FBgn0032167	CG5853	CG5853	-1.55	0.00
FBgn0004133	blow	blown fuse	-1.55	0.00
FBgn0027779	VhaSFD	Vacuolar H[+]-ATPase SFD subunit	-1.55	0.00
FBgn0011232	scat	scattered	-1.55	0.00
FBgn0029722	CG7024	CG7024	-1.55	0.00
FBgn0015608	Ca-beta	Ca[2+]-channel-protein-beta-subunit	-1.55	0.00
FBgn0039676	ppk20	pickpocket 20	-1.55	0.00
FBgn0030643	CG15641	CG15641	-1.55	0.00
FBgn0035392	CG1271	CG1271	-1.55	0.00
FBgn0039931	sphinx	sphinx	-1.55	0.00
FBgn0000163	baz	bazooka	-1.55	0.00

FBgn0037102	CG7172	CG7172	-1.55	0.00
FBgn0050118	CG30118	CG30118	-1.55	0.00
FBgn0015240	Hr96	Hormone receptor-like in 96	-1.55	0.00
FBgn0040536	CG12418	CG12418	-1.56	0.00
FBgn0030679	CG8206	CG8206	-1.56	0.00
FBgn0034267	CG4984	CG4984	-1.56	0.00
FBgn0039780	PH4alphaNE1	prolyl-4-hydroxylase-alpha NE1	-1.56	0.00
FBgn0023495	Lip3	Lip3	-1.56	0.01
FBgn0031129	CG1324	CG1324	-1.56	0.00
FBgn0031639	mRpS2	mitochondrial ribosomal protein S2	-1.56	0.00
FBgn0036833	CG3819	CG3819	-1.56	0.00
FBgn0035282	CG13936	CG13936	-1.56	0.00
FBgn0001332	L	Lobe	-1.56	0.00
FBgn0024189	sns	sticks and stones	-1.56	0.00
FBgn0036994	CG5199	CG5199	-1.56	0.00
FBgn0021944	Coprox	Coproporphyrinogen oxidase	-1.56	0.00
FBgn0033102	Epac	Epac	-1.56	0.00
FBgn0039645	CG11898	CG11898	-1.56	0.00
FBgn0015808	ScpX	Sterol carrier protein X-related thiolase	-1.56	0.00
FBgn0016047	nompA	no mechanoreceptor potential A	-1.56	0.00
FBgn0040798	CG13069	CG13069	-1.56	0.05
FBgn0011284	RpS4	Ribosomal protein S4	-1.56	0.00
FBgn0034197	CG15918	CG15918	-1.56	0.00
FBgn0002023	Lim3	Lim3	-1.56	0.00
FBgn0036334	CG11267	CG11267	-1.56	0.00
FBgn0028931	CG16863	CG16863	-1.57	0.00
FBgn0038168	CG9591	CG9591	-1.57	0.00
FBgn0035954	Doc3	Dorsocross3	-1.57	0.00
FBgn0052485	CG32485	CG32485	-1.57	0.00
FBgn0024988	CG14801	CG14801	-1.57	0.00
FBgn0027794	CG14786	CG14786	-1.57	0.00
FBgn0030346	CG11802	CG11802	-1.57	0.00
FBgn0034579	mRpL54	mitochondrial ribosomal protein L54	-1.57	0.00
FBgn0040281	Aplip1	APP-like protein interacting protein 1	-1.57	0.00
FBgn0038450	CG17560	CG17560	-1.57	0.00
FBgn0029629	CG8636	CG8636	-1.57	0.00

FBgn0040628	CG18672	CG18672	-1.57	0.01
FBgn0035032	CG4692	CG4692	-1.57	0.00
FBgn0010241	Mdr50	Multi drug resistance 50	-1.57	0.00
FBgn0052454	CG32454	CG32454	-1.57	0.00
FBgn0035126	CG13882	CG13882	-1.57	0.00
FBgn0033507	CG12909	CG12909	-1.57	0.00
FBgn0042083	CG3267	CG3267	-1.57	0.00
FBgn0005674	Aats-glupro	Glutamyl-prolyl-tRNA synthetase	-1.57	0.00
FBgn0040773	CG2249	CG2249	-1.57	0.00
FBgn0037093	CG7597	CG7597	-1.57	0.00
FBgn0034238	CG18432	CG18432	-1.57	0.00
FBgn0037469	CG1939	CG1939	-1.57	0.00
FBgn0029639	CG14419	CG14419	-1.57	0.00
FBgn0053156	CG33156	CG33156	-1.57	0.00
FBgn0030839	CG5613	CG5613	-1.57	0.00
FBgn0032882	CG9320	CG9320	-1.57	0.00
FBgn0029609	l(1)G0144	lethal (1) G0144	-1.57	0.00
FBgn0033380	Phax	Phosphorylated adaptor for RNA export	-1.57	0.00
FBgn0023169	SNF1A	SNF1A/AMP-activated protein kinase	-1.57	0.00
FBgn0033808	CG4627	CG4627	-1.57	0.00
FBgn0029850	CG14446	CG14446	-1.57	0.00
FBgn0026079	CG6133	CG6133	-1.57	0.00
FBgn0036698	CG7724	CG7724	-1.57	0.00
FBgn0004655	wapl	wings apart-like	-1.58	0.00
FBgn0053146	Mctp	Multiple C2 domain and transmembrane region protein	-1.58	0.00
FBgn0035503	nab	nab	-1.58	0.00
FBgn0005585	Crc	Calreticulin	-1.58	0.00
FBgn0015576	alpha-Est8	alpha-Esterase-8	-1.58	0.00
FBgn0053154	CG13175	CG13175	-1.58	0.00
FBgn0033058	CG14593	CG14593	-1.58	0.00
FBgn0024998	CG2685	CG2685	-1.58	0.00
FBgn0013763	Chit	Chitinase-like	-1.58	0.00
FBgn0032131	CG3841	CG3841	-1.58	0.00
FBgn0022700	Cht4	Chitinase 4	-1.58	0.00
FBgn0037530	CG2943	CG2943	-1.58	0.00
FBgn0051797	CG31797	CG31797	-1.58	0.00

FBgn0039580	Gfat2	Glutamine:fructose-6-phosphate aminotransferase 2	-1.58	0.00
FBgn0040801	CG13053	CG13053	-1.58	0.00
FBgn0034877	CG17280	CG17280	-1.58	0.00
FBgn0026173	slim	scruin like at the midline	-1.58	0.00
FBgn0034888	CG5431	CG5431	-1.58	0.00
FBgn0034087	CG8443	CG8443	-1.58	0.00
FBgn0031050	Arp11	Arp11	-1.58	0.00
FBgn0039112	CG10219	CG10219	-1.58	0.00
FBgn0033075	Pld	Phospholipase D	-1.58	0.00
FBgn0036272	CG4300	CG4300	-1.58	0.00
FBgn0023441	fus	fusilli	-1.59	0.00
FBgn0039667	CG2010	CG2010	-1.59	0.00
FBgn0003475	spir	spire	-1.59	0.00
FBgn0037610	CG8043	CG8043	-1.59	0.00
FBgn0015788	ltd	lightoid	-1.59	0.00
FBgn0030265	CG11126	CG11126	-1.59	0.00
FBgn0030688	CG8952	CG8952	-1.59	0.00
FBgn0020623	Pal	Peptidyl-alpha-hydroxyglycine-alpha-amidating lyase	-1.59	0.00
FBgn0028646	aralar1	aralar1	-1.59	0.00
FBgn0033224	CG1553	CG1553	-1.59	0.00
FBgn0035029	IFa	IFamide	-1.59	0.00
FBgn0033045	CG12551	CG12551	-1.59	0.00
FBgn0036612	CG4998	CG4998	-1.59	0.00
FBgn0021825	Dmn	Dynamitin	-1.59	0.00
FBgn0033783	CG17019	CG17019	-1.59	0.00
FBgn0027836	Dgp-1	Dgp-1	-1.59	0.00
FBgn0052648	Pde9	Phosphodiesterase 9	-1.59	0.00
FBgn0039840	CG11340	CG11340	-1.59	0.00
FBgn0050097	Menl-1	Menl-1	-1.59	0.00
FBgn0033160	CG11107	CG11107	-1.59	0.00
FBgn0052750	CG32750	CG32750	-1.59	0.00
FBgn0025645	CG3598	CG3598	-1.59	0.00
FBgn0025627	CG4194	CG4194	-1.59	0.00
FBgn0015591	Ast	Allatostatin	-1.59	0.00
FBgn0015571	alpha-Est3	alpha-Esterase-3	-1.60	0.00
FBgn0010808	l(3)03670	lethal (3) 03670	-1.60	0.00

FBgn0031514	CG3332	CG3332	-1.60	0.00
FBgn0030605	CG5548	CG5548	-1.60	0.00
FBgn0033454	CG1671	CG1671	-1.60	0.00
FBgn0035651	CG33523	CG33523	-1.60	0.00
FBgn0053214	CG33214	CG33214	-1.60	0.00
FBgn0000357	Cp18	Chorion protein 18	-1.60	0.00
FBgn0028665	VhaAC39	VhaAC39	-1.60	0.00
FBgn0037051	CG10565	CG10565	-1.60	0.00
FBgn0025937	cmp44E	conserved membrane protein at 44E	-1.60	0.00
FBgn0032832	CG10662	CG10662	-1.60	0.00
FBgn0037969	CG18347	CG18347	-1.60	0.00
FBgn0053113	Rtnl1	Rtnl1	-1.60	0.00
FBgn0038376	CG4225	CG4225	-1.60	0.00
FBgn0029716	CG3546	CG3546	-1.60	0.00
FBgn0015001	iotaTry	iotaTrypsin	-1.60	0.00
FBgn0029994	CG2254	CG2254	-1.60	0.00
FBgn0036592	CG13049	CG13049	-1.60	0.00
FBgn0023458	CG3585	CG3585	-1.60	0.00
FBgn0024997	CG2681	CG2681	-1.60	0.00
FBgn0037384	CG10981	CG10981	-1.60	0.00
FBgn0010621	Cct5	T-complex Chaperonin 5	-1.60	0.00
FBgn0003557	Su(dx)	Suppressor of deltex	-1.60	0.00
FBgn0003065	CG2150	CG2150	-1.60	0.00
FBgn0040616	CG12871	CG12871	-1.60	0.00
FBgn0034293	CG14495	CG14495	-1.61	0.00
FBgn0039443	CG14242	CG14242	-1.61	0.00
FBgn0039648	CG14515	CG14515	-1.61	0.00
FBgn0037057	CG10512	CG10512	-1.61	0.00
FBgn0039820	CG15554	CG15554	-1.61	0.00
FBgn0034793	asrij	asrij	-1.61	0.00
FBgn0003861	trp	transient receptor potential	-1.61	0.00
FBgn0038276	CG7026	CG7026	-1.61	0.00
FBgn0035271	CG2021	CG2021	-1.61	0.00
FBgn0026718	fu12	fu12	-1.61	0.00
FBgn0004779	Ccp84Ae	Ccp84Ae	-1.61	0.00
FBgn0032883	CG9323	CG9323	-1.61	0.00

FBgn0027453	Dnz1	DNZDHHC/NEW1 zinc finger protein 11	-1.61	0.00
FBgn0036975	CG5618	CG5618	-1.61	0.00
FBgn0031085	CG9570	CG9570	-1.61	0.00
FBgn0036589	CG13067	CG13067	-1.61	0.00
FBgn0053209	comm3	comm3	-1.61	0.00
FBgn0051937	CG31937	CG31937	-1.61	0.00
FBgn0035371	CG9977	CG9977	-1.61	0.00
FBgn0031117	CG1702	CG1702	-1.61	0.00
FBgn0034899	CG13560	CG13560	-1.62	0.00
FBgn0031945	CG7191	CG7191	-1.62	0.00
FBgn0037414	Osi7	Osiris 7	-1.62	0.00
FBgn0027560	Tps1	Trehalose-6-phosphate synthase 1	-1.62	0.00
FBgn0029748	CG15464	CG15464	-1.62	0.00
FBgn0030975	CG7349	CG7349	-1.62	0.00
FBgn0036623	CG4729	CG4729	-1.62	0.00
FBgn0034718	wdp	windpipe	-1.62	0.00
FBgn0040257	Ugt86Dc	Ugt86Dc	-1.62	0.00
FBgn0010294	ng2	new glue 2	-1.62	0.00
FBgn0039689	CG7598	CG7598	-1.62	0.00
FBgn0032449	CG17036	CG17036	-1.62	0.00
FBgn0032646	CG6412	CG6412	-1.62	0.00
FBgn0031002	CG14196	CG14196	-1.62	0.00
FBgn0032945	CG8665	CG8665	-1.62	0.01
FBgn0000644	Fcp3C	Follicle cell protein 3C	-1.62	0.00
FBgn0034427	CG10474	CG10474	-1.62	0.00
FBgn0038130	CG8630	CG8630	-1.62	0.00
FBgn0033204	CG2065	CG2065	-1.62	0.00
FBgn0035361	Aats-pro	Prolyl-tRNA synthetase	-1.62	0.00
FBgn0050350	CG30350	CG30350	-1.62	0.00
FBgn0015234	HLH106	Helix loop helix protein 106	-1.62	0.00
FBgn0033584	CG7737	CG7737	-1.62	0.00
FBgn0029967	CG15327	CG15327	-1.62	0.00
FBgn0029851	CG14445	CG14445	-1.63	0.00
FBgn0034420	CG10737	CG10737	-1.63	0.01
FBgn0031418	CG3609	CG3609	-1.63	0.00
FBgn0039856	CG1774	CG1774	-1.63	0.00

FBgn0035930	CG13307	CG13307	-1.63	0.00
FBgn0016687	Nurf-38	Nucleosome remodeling factor - 38kD	-1.63	0.00
FBgn0033733	CG8834	CG8834	-1.63	0.00
FBgn0023407	B4	B4	-1.63	0.00
FBgn0036318	CG11009	CG11009	-1.63	0.00
FBgn0002069	Aats-asp	Aspartyl-tRNA synthetase	-1.63	0.00
FBgn0017429	CG5989	CG5989	-1.63	0.00
FBgn0033914	CG8479	CG8479	-1.63	0.00
FBgn0050115	CG30115	CG30115	-1.63	0.00
FBgn0015245	Hsp60	Heat shock protein 60	-1.63	0.00
FBgn0036135	mRpL2	mitochondrial ribosomal protein L2	-1.63	0.00
FBgn0032719	CG17321	CG17321	-1.63	0.00
FBgn0035154	CG3344	CG3344	-1.63	0.00
FBgn0031812	CG9523	CG9523	-1.63	0.00
FBgn0035231	Cct2	CTP:phosphocholine cytidylyltransferase 2	-1.63	0.00
FBgn0004643	mit(1)15	mitotic 15	-1.63	0.00
FBgn0024510	dlt	discs lost	-1.63	0.00
FBgn0039541	CG12876	CG12876	-1.64	0.00
FBgn0000592	Est-6	Esterase 6	-1.64	0.00
FBgn0031438	Drp1	Dynamin related protein 1	-1.64	0.00
FBgn0034434	Rgk1	Rgk1	-1.64	0.00
FBgn0003074	Pgi	Phosphoglucose isomerase	-1.64	0.00
FBgn0001219	Hsc70-4	Heat shock protein cognate 4	-1.64	0.00
FBgn0027529	CG8920	CG8920	-1.64	0.00
FBgn0039472	CG17192	CG17192	-1.64	0.01
FBgn0030718	CG9172	CG9172	-1.64	0.00
FBgn0033417	Camta	Calmodulin-binding transcription activator	-1.64	0.00
FBgn0034406	Jheh3	Juvenile hormone epoxide hydrolase 3	-1.64	0.00
FBgn0036763	CG7441	CG7441	-1.64	0.00
FBgn0003996	w	white	-1.64	0.00
FBgn0039324	CG10553	CG10553	-1.64	0.00
FBgn0051752	CG31752	CG31752	-1.64	0.00
FBgn0011554	etaTry	etaTrypsin	-1.64	0.00
FBgn0037671	CG8444	CG8444	-1.64	0.00
FBgn0036211	CG5946	CG5946	-1.64	0.00
FBgn0015040	Cyp9c1	Cytochrome P450-9c1	-1.64	0.00

FBgn0005614	trpl	trp-like	-1.64	0.00
FBgn0027917	CG33968	CG33968	-1.64	0.00
FBgn0035409	CG14963	CG14963	-1.64	0.00
FBgn0004778	Ccp84Af	Ccp84Af	-1.64	0.00
FBgn0031807	CG13981	CG13981	-1.64	0.00
FBgn0034225	CG4827	CG4827	-1.64	0.00
FBgn0033033	scarface	scarface	-1.65	0.00
FBgn0034119	l(2)k07824	lethal (2) k07824	-1.65	0.00
FBgn0011700	spn-A	spindle A	-1.65	0.00
FBgn0016119	ATPsyn-Cf6	ATPase coupling factor 6	-1.65	0.00
FBgn0034501	CG13868	CG13868	-1.65	0.00
FBgn0027600	CG4778	CG4778	-1.65	0.00
FBgn0032812	CG10263	CG10263	-1.65	0.00
FBgn0040722	CG14349	CG14349	-1.65	0.00
FBgn0039745	CG7950	CG7950	-1.65	0.00
FBgn0037354	CG12171	CG12171	-1.65	0.00
FBgn0022097	Vha36	Vha36	-1.65	0.00
FBgn0029868	CG3446	CG3446	-1.65	0.00
FBgn0039182	CG5728	CG5728	-1.65	0.00
FBgn0027106	inx7	innexin 7	-1.65	0.00
FBgn0010043	GstD7	Glutathione S transferase D7	-1.65	0.00
FBgn0035325	CG13806	CG13806	-1.65	0.00
FBgn0031806	slam	slow as molasses	-1.65	0.00
FBgn0024238	Fim	Fimbrin	-1.65	0.00
FBgn0037293	CG12007	CG12007	-1.65	0.00
FBgn0033714	garz	gartenzwerg	-1.66	0.00
FBgn0032715	CG17597	CG17597	-1.66	0.00
FBgn0036588	CG13068	CG13068	-1.66	0.00
FBgn0038139	CG8795	CG8795	-1.66	0.00
FBgn0039567	betaTub97EF	beta-Tubulin at 97EF	-1.66	0.00
FBgn0029761	SK	small conductance calcium-activated potassium channel	-1.66	0.00
FBgn0023512	eIF2B-epsilon	eIF2B-epsilon	-1.66	0.00
FBgn0010391	Gtp-bp	GTP-binding protein	-1.66	0.00
FBgn0039052	CG6733	CG6733	-1.66	0.00
FBgn0040319	Gclc	Glutamate-cysteine ligase catalytic subunit	-1.66	0.00
FBgn0032387	CG16965	CG16965	-1.66	0.00

FBgn0031178	CG10918	CG10918	-1.66	0.00
FBgn0031832	CG9596	CG9596	-1.66	0.00
FBgn0029084	gom	gomdanji	-1.66	0.00
FBgn0027607	CG8230	CG8230	-1.66	0.00
FBgn0035438	PHGPx	PHGPx	-1.66	0.00
FBgn0039669	CG2014	CG2014	-1.66	0.00
FBgn0036594	CG13047	CG13047	-1.66	0.00
FBgn0019929	Ser7	Ser7	-1.67	0.00
FBgn0033375	CG8078	CG8078	-1.67	0.00
FBgn0040064	yip2	yippee interacting protein 2	-1.67	0.00
FBgn0036361	CG10154	CG10154	-1.67	0.00
FBgn0035495	CG14989	CG14989	-1.67	0.00
FBgn0022023	eIF-3p40	Eukaryotic initiation factor 3 p40 subunit	-1.67	0.00
FBgn0035247	metl	methyltransferase-like	-1.67	0.00
FBgn0037341	CG12746	CG12746	-1.67	0.00
FBgn0041707	7B2	7B2	-1.67	0.00
FBgn0031991	CG8506	CG8506	-1.67	0.00
FBgn0038949	CG7071	CG7071	-1.67	0.00
FBgn0031432	Cyp309a1	Cyp309a1	-1.67	0.00
FBgn0040895	CG14773	CG14773	-1.67	0.00
FBgn0038918	CG6690	CG6690	-1.67	0.00
FBgn0037165	CG11437	CG11437	-1.67	0.00
FBgn0033868	CG13340	CG13340	-1.68	0.00
FBgn0031818	CG9536	CG9536	-1.68	0.00
FBgn0053075	Shawn	Shawn	-1.68	0.00
FBgn0032315	CG14069	CG14069	-1.68	0.00
FBgn0035724	CG10064	CG10064	-1.68	0.00
FBgn0025836	RhoGAP1A	RhoGAP1A	-1.68	0.00
FBgn0035039	CG3608	CG3608	-1.68	0.00
FBgn0030141	Gga	Gga	-1.68	0.00
FBgn0003328	scb	scab	-1.68	0.00
FBgn0033394	CG13740	CG13740	-1.68	0.00
FBgn0039882	CG11576	CG11576	-1.68	0.00
FBgn0051757	CG31757	CG31757	-1.68	0.00
FBgn0037704	by	blistery	-1.68	0.00
FBgn0002441	l(3)mbt	lethal (3) malignant brain tumor	-1.68	0.00

FBgn0004834	Gprk2	G protein-coupled receptor kinase 2	-1.68	0.00
FBgn0039203	CG13618	CG13618	-1.68	0.00
FBgn0038924	CG6028	CG6028	-1.68	0.00
FBgn0036638	CG13033	CG13033	-1.68	0.00
FBgn0034482	CG18416	CG18416	-1.69	0.00
FBgn0038742	Arc42	Arc42	-1.69	0.00
FBgn0031392	CG7263	CG7263	-1.69	0.00
FBgn0037488	CG14607	CG14607	-1.69	0.00
FBgn0030263	CG2076	CG2076	-1.69	0.00
FBgn0037501	CG10101	CG10101	-1.69	0.00
FBgn0029502	COQ7	COQ7	-1.69	0.00
FBgn0036774	mRpS26	mitochondrial ribosomal protein S26	-1.69	0.00
FBgn0039656	CG11951	CG11951	-1.69	0.00
FBgn0038734	CG11453	CG11453	-1.69	0.00
FBgn0035243	CG13926	CG13926	-1.69	0.00
FBgn0032049	CG13095	CG13095	-1.69	0.00
FBgn0033366	Ance-4	Ance-4	-1.69	0.00
FBgn0008636	hbn	homeobrain	-1.69	0.00
FBgn0037261	CG9775	CG9775	-1.69	0.00
FBgn0051792	CG31792	CG31792	-1.69	0.00
FBgn0031428	CG9886	CG9886	-1.69	0.00
FBgn0030403	CG1824	CG1824	-1.70	0.00
FBgn0024558	Dph5	Diphthamide methyltransferase	-1.70	0.00
FBgn0004777	Ccp84Ag	Ccp84Ag	-1.70	0.00
FBgn0033543	CG12338	CG12338	-1.70	0.00
FBgn0031011	CG8034	CG8034	-1.70	0.00
FBgn0035190	CG13913	CG13913	-1.70	0.00
FBgn0035548	CG15023	CG15023	-1.70	0.00
FBgn0032908	CG9270	CG9270	-1.70	0.00
FBgn0032810	CG13077	CG13077	-1.70	0.00
FBgn0033866	CG6280	CG6280	-1.70	0.00
FBgn0034289	CG10910	CG10910	-1.70	0.00
FBgn0014024	Rnp4F	RNA-binding protein 4F	-1.70	0.00
FBgn0013307	Odc1	Ornithine decarboxylase 1	-1.70	0.00
FBgn0037140	CG7442	CG7442	-1.70	0.00
FBgn0035189	CG9119	CG9119	-1.70	0.00

FBgn0052918	CG15891	CG15891	-1.71	0.00
FBgn0040394	CG16903	CG16903	-1.71	0.00
FBgn0028480	CG17841	CG17841	-1.71	0.00
FBgn0034957	CG3121	CG3121	-1.71	0.00
FBgn0034851	CG11079	CG11079	-1.71	0.00
FBgn0014127	barr	barren	-1.71	0.00
FBgn0036144	GlcAT-P	GlcAT-P	-1.71	0.00
FBgn0033308	CG8736	CG8736	-1.71	0.00
FBgn0052604	l(1)G0007	lethal (1) G0007	-1.71	0.00
FBgn0033083	Dpit47	DNA polymerase interacting tpr containing protein of 47kD	-1.71	0.00
FBgn0037684	CG8129	CG8129	-1.71	0.00
FBgn0011555	thetaTry	thetaTrypsin	-1.71	0.00
FBgn0036157	CG7560	CG7560	-1.72	0.00
FBgn0050291	CG30291	CG30291	-1.72	0.00
FBgn0031478	CG8814	CG8814	-1.72	0.00
FBgn0003060	CG9757	CG9757	-1.72	0.00
FBgn0051006	CG31006	CG31006	-1.72	0.00
FBgn0051674	CG31674	CG31674	-1.72	0.00
FBgn0037399	Or83c	Odorant receptor 83c	-1.72	0.00
FBgn0013432	bcn92	bcn92	-1.72	0.00
FBgn0033645	CG13196	CG13196	-1.72	0.00
FBgn0014002	Pdi	Protein disulfide isomerase	-1.72	0.00
FBgn0028950	CG15255	CG15255	-1.72	0.00
FBgn0033351	CG8235	CG8235	-1.72	0.00
FBgn0034298	CG14497	CG14497	-1.72	0.00
FBgn0031817	CG9531	CG9531	-1.72	0.00
FBgn0039555	mRpS22	mitochondrial ribosomal protein S22	-1.73	0.00
FBgn0032983	CG5922	CG5922	-1.73	0.00
FBgn0032075	Tsp29Fb	Tetraspanin 29Fb	-1.73	0.00
FBgn0015277	Pi3K59F	Phosphotidylinositol 3 kinase 59F	-1.73	0.00
FBgn0027556	CG4928	CG4928	-1.73	0.00
FBgn0037329	CG12162	CG12162	-1.73	0.00
FBgn0035666	Jon65Aii	Jonah 65Aii	-1.73	0.00
FBgn0040691	CG13915	CG13915	-1.73	0.00
FBgn0035950	CG5288	CG5288	-1.73	0.00
FBgn0002930	nec	necrotic	-1.73	0.00

FBgn0039871	l(3)s1921	lethal (3) s1921	-1.73	0.00
FBgn0030981	CG14191	CG14191	-1.73	0.00
FBgn0016075	vkg	viking	-1.73	0.00
FBgn0004507	GlyP	Glycogen phosphorylase	-1.73	0.00
FBgn0039140	Miro	Mitochondrial Rho	-1.73	0.00
FBgn0037078	CG12971	CG12971	-1.73	0.00
FBgn0004867	sop	string of pearls	-1.73	0.00
FBgn0051004	CG31004	CG31004	-1.73	0.00
FBgn0039817	CG15553	CG15553	-1.73	0.00
FBgn0031000	CG7876	CG7876	-1.73	0.00
FBgn0028497	CG3530	CG3530	-1.73	0.00
FBgn0052392	CG32392	CG32392	-1.73	0.00
FBgn0037822	CG14683	CG14683	-1.73	0.00
FBgn0015316	Try29F	Trypsin 29F	-1.74	0.00
FBgn0033206	CG12042	CG12042	-1.74	0.00
FBgn0010053	Jheh1	Juvenile hormone epoxide hydrolase 1	-1.74	0.00
FBgn0015570	alpha-Est2	alpha-Esterase-2	-1.74	0.00
FBgn0034723	CG13506	CG13506	-1.74	0.00
FBgn0029896	CG3168	CG3168	-1.74	0.00
FBgn0014863	Mlp84B	Muscle LIM protein at 84B	-1.74	0.00
FBgn0032397	Tom70	Translocase of outer membrane 70	-1.74	0.00
FBgn0027580	CG1516	CG1516	-1.74	0.00
FBgn0040797	CG13066	CG13066	-1.74	0.00
FBgn0031092	CG9577	CG9577	-1.74	0.00
FBgn0034565	CG15650	CG15650	-1.74	0.00
FBgn0046281	CG33340	CG33340	-1.74	0.00
FBgn0036824	CG3902	CG3902	-1.74	0.00
FBgn0032266	CG18302	CG18302	-1.75	0.00
FBgn0036162	CG6140	CG6140	-1.75	0.00
FBgn0035388	CG2162	CG2162	-1.75	0.00
FBgn0039828	CG1542	CG1542	-1.75	0.00
FBgn0034840	CG3124	CG3124	-1.75	0.00
FBgn0052793	CG32793	CG32793	-1.75	0.00
FBgn0030737	CG9914	CG9914	-1.75	0.00
FBgn0052549	CG32549	CG32549	-1.75	0.00
FBgn0027615	CG6404	CG6404	-1.75	0.00

FBgn0025593	Glut1	Glucose transporter 1	-1.75	0.00
FBgn0031728	CG7235	CG7235	-1.75	0.00
FBgn0035077	CG9083	CG9083	-1.75	0.00
FBgn0032512	CG9305	CG9305	-1.75	0.00
FBgn0023516	CG14815	CG14815	-1.75	0.00
FBgn0025682	scf	supercoiling factor	-1.75	0.00
FBgn0025839	CG3621	CG3621	-1.76	0.00
FBgn0036362	CG10725	CG10725	-1.76	0.00
FBgn0051477	CG31477	CG31477	-1.76	0.00
FBgn0034215	CG4802	CG4802	-1.76	0.00
FBgn0002021	l(2)37Bb	lethal (2) 37Bb	-1.76	0.00
FBgn0034297	CG14496	CG14496	-1.76	0.00
FBgn0033158	CG12164	CG12164	-1.76	0.00
FBgn0052316	CG33791	CG33791	-1.76	0.00
FBgn0033292	Cyp4ad1	Cyp4ad1	-1.76	0.00
FBgn0026729	axo	axotactin	-1.76	0.00
FBgn0036607	CG13059	CG13059	-1.77	0.00
FBgn0028737	Ef1beta	Elongation factor 1 beta	-1.77	0.00
FBgn0034075	Asph	Aspartyl beta-hydroxylase	-1.77	0.00
FBgn0034134	CG4750	CG4750	-1.77	0.00
FBgn0027588	CG14476	CG14476	-1.77	0.00
FBgn0037270	CG9769	CG9769	-1.77	0.00
FBgn0033401	CG1968	CG1968	-1.77	0.00
FBgn0037018	CG4042	CG4042	-1.77	0.00
FBgn0003691	th	thread	-1.77	0.00
FBgn0036351	CG14107	CG14107	-1.77	0.00
FBgn0040625	CG18682	CG18682	-1.77	0.00
FBgn0031860	CG11236	CG11236	-1.77	0.00
FBgn0036871	CG14096	CG14096	-1.77	0.00
FBgn0033673	CG8298	CG8298	-1.78	0.00
FBgn0039118	CG10208	CG10208	-1.78	0.00
FBgn0034940	CG16787	CG16787	-1.78	0.00
FBgn0052445	CG32445	CG32445	-1.78	0.00
FBgn0001142	Gs1	Glutamine synthetase 1	-1.78	0.00
FBgn0003057	CG10598	CG10598	-1.78	0.00
FBgn0028916	CG33090	CG33090	-1.78	0.00

FBgn0031537	sec5	sec5	-1.78	0.00
FBgn0024973	CG2701	CG2701	-1.78	0.00
FBgn0037065	CG12974	CG12974	-1.78	0.00
FBgn0021979	l(2)k09913	lethal (2) k09913	-1.78	0.00
FBgn0011455	l(3)neo18	lethal (3) neo18	-1.78	0.00
FBgn0031942	CG7203	CG7203	-1.78	0.00
FBgn0035203	CG9149	CG9149	-1.78	0.00
FBgn0037973	CG18547	CG18547	-1.78	0.00
FBgn0033601	CG9076	CG9076	-1.78	0.00
FBgn0038482	CG4053	CG4053	-1.79	0.00
FBgn0003076	Pgm	Phosphogluconate mutase	-1.79	0.00
FBgn0031312	l(2)k00619	lethal (2) k00619	-1.79	0.00
FBgn0025383	CG14780	CG14780	-1.79	0.00
FBgn0037566	mRpL1	mitochondrial ribosomal protein L1	-1.79	0.00
FBgn0028543	CG31839	CG31839	-1.79	0.00
FBgn0016693	Past1	Putative Achaete Scute Target 1	-1.79	0.00
FBgn0002174	l(2)tid	lethal (2) tumorous imaginal discs	-1.79	0.00
FBgn0037653	CG11982	CG11982	-1.79	0.00
FBgn0037846	CG6574	CG6574	-1.79	0.00
FBgn0028436	ECSIT	ECSIT	-1.79	0.00
FBgn0029804	CG3097	CG3097	-1.80	0.00
FBgn0031305	Iris	Iris	-1.80	0.00
FBgn0039219	CG13630	CG13630	-1.80	0.00
FBgn0034147	CG5197	CG5197	-1.80	0.00
FBgn0033188	CG1600	CG1600	-1.80	0.00
FBgn0001230	Hsp68	Heat shock protein 68	-1.80	0.02
FBgn0038318	CG6236	CG6236	-1.80	0.00
FBgn0011787	mRpL12	mitochondrial ribosomal protein L12	-1.81	0.00
FBgn0028492	Aats-asn	Asparaginyl-tRNA synthetase	-1.81	0.00
FBgn0052736	CG32736	CG32736	-1.81	0.00
FBgn0030821	CG5010	CG5010	-1.81	0.00
FBgn0004878	cas	castor	-1.81	0.00
FBgn0015031	cype	cyclope	-1.81	0.00
FBgn0027081	Aats-thr	Threonyl-tRNA synthetase	-1.81	0.00
FBgn0034425	CG11906	CG11906	-1.81	0.00
FBgn0003366	sev	sevenless	-1.81	0.00

FBgn0031500	CG17221	CG17221	-1.81	0.00
FBgn0011603	ine	inebriated	-1.81	0.00
FBgn0033637	l(2)k14708	lethal (2) k14708	-1.82	0.00
FBgn0037678	CG16749	CG16749	-1.82	0.00
FBgn0025725	alphaCop	alpha-coatomer protein	-1.82	0.00
FBgn0024897	b6	b6	-1.82	0.00
FBgn0029891	CG4523	CG4523	-1.82	0.00
FBgn0036532	CG13445	CG13445	-1.82	0.00
FBgn0002031	l(2)37Cc	lethal (2) 37Cc	-1.82	0.00
FBgn0011834	Ser6	Serine protease 6	-1.82	0.00
FBgn0052533	CG32533	CG32533	-1.82	0.00
FBgn0034204	CG10953	CG10953	-1.82	0.01
FBgn0030908	CG15057	CG15057	-1.82	0.00
FBgn0014023	Rlc1	Rlc1	-1.82	0.00
FBgn0037144	CG7458	CG7458	-1.82	0.00
FBgn0028495	CG18616	CG18616	-1.82	0.00
FBgn0034971	CG3209	CG3209	-1.83	0.00
FBgn0033397	Cyp4p3	Cyp4p3	-1.83	0.00
FBgn0036872	CG12519	CG12519	-1.83	0.00
FBgn0031303	smi21F	smell impaired 21F	-1.83	0.00
FBgn0035931	CG13312	CG13312	-1.83	0.00
FBgn0027785	NP15.6	NP15.6	-1.83	0.00
FBgn0034494	CG10444	CG10444	-1.83	0.00
FBgn0029888	CG3192	CG3192	-1.83	0.00
FBgn0035947	CG5064	CG5064	-1.83	0.00
FBgn0038049	CG5844	CG5844	-1.84	0.00
FBgn0033524	Cyp49a1	Cyp49a1	-1.84	0.00
FBgn0038481	CG17475	CG17475	-1.84	0.00
FBgn0029176	Ef1gamma	Ef1gamma	-1.84	0.00
FBgn0030824	CG5070	CG5070	-1.84	0.00
FBgn0033428	Updo	Updo	-1.84	0.00
FBgn0032136	CG15828	CG15828	-1.84	0.00
FBgn0030934	Aats-his	Histidyl-tRNA synthetase	-1.84	0.00
FBgn0039561	CG4963	CG4963	-1.84	0.00
FBgn0026761	Trap1	Trap1	-1.84	0.00
FBgn0003067	Pepck	Phosphoenolpyruvate carboxykinase	-1.84	0.00

FBgn0031021	CG12203	CG12203	-1.84	0.00
FBgn0028573	prc	pericardin	-1.84	0.00
FBgn0037420	CG15597	CG15597	-1.85	0.00
FBgn0036182	CG6084	CG6084	-1.85	0.00
FBgn0034351	CG5190	CG5190	-1.85	0.00
FBgn0026372	RpL23A	Ribosomal protein L23A	-1.85	0.00
FBgn0034201	CG17290	CG17290	-1.85	0.00
FBgn0034343	GstE9	Glutathione S transferase E9	-1.85	0.00
FBgn0003075	Pgk	Phosphoglycerate kinase	-1.85	0.00
FBgn0028962	Aats-ala-m	mitochondrial alanyl-tRNA synthetase	-1.85	0.00
FBgn0010504	l(2)02045	lethal (2) 02045	-1.85	0.00
FBgn0003159	ptr	proximal to raf	-1.86	0.00
FBgn0033391	CG8026	CG8026	-1.86	0.00
FBgn0003178	PyK	Pyruvate kinase	-1.86	0.00
FBgn0051086	CG31086	CG31086	-1.86	0.00
FBgn0000630	f	forked	-1.86	0.00
FBgn0037762	CG16905	CG16905	-1.86	0.00
FBgn0035792	CG7548	CG7548	-1.86	0.00
FBgn0033751	CG8818	CG8818	-1.86	0.00
FBgn0031810	CG9511	CG9511	-1.87	0.00
FBgn0039674	CG1907	CG1907	-1.87	0.00
FBgn0025595	GRHR	Gonadotropin-releasing hormone receptor	-1.87	0.00
FBgn0030837	CG8661	CG8661	-1.87	0.00
FBgn0029762	CG3252	CG3252	-1.87	0.00
FBgn0038552	CG18012	CG18012	-1.87	0.00
FBgn0050272	CG30272	CG30272	-1.87	0.00
FBgn0052354	CG32354	CG32354	-1.87	0.00
FBgn0035431	CG14968	CG14968	-1.87	0.00
FBgn0029811	CG4052	CG4052	-1.87	0.00
FBgn0035787	CG8543	CG8543	-1.87	0.00
FBgn0003515	st	scarlet	-1.88	0.00
FBgn0030510	CG12177	CG12177	-1.88	0.00
FBgn0029897	RpL17	Ribosomal protein L17	-1.88	0.00
FBgn0014906	CG3488	CG3488	-1.88	0.00
FBgn0039428	CG14237	CG14237	-1.88	0.00
FBgn0051052	CG31052	CG31052	-1.88	0.00

FBgn0001248	Idh	Isocitrate dehydrogenase	-1.88	0.00
FBgn0034784	CG9826	CG9826	-1.88	0.00
FBgn0035886	Jon66Ci	Jonah 66Ci	-1.88	0.00
FBgn0032985	Mgstl-Psi	Microsomal glutathione S-transferase-like pseudogene	-1.88	0.00
FBgn0011577	dally	division abnormally delayed	-1.88	0.00
FBgn0000326	clt	cricklet	-1.89	0.00
FBgn0037371	CG2097	CG2097	-1.89	0.00
FBgn0033502	CG12910	CG12910	-1.89	0.00
FBgn0001565	Hlc	Helicase	-1.89	0.00
FBgn0035632	CG10575	CG10575	-1.89	0.00
FBgn0022359	Sodh-2	Sorbitol dehydrogenase-2	-1.89	0.00
FBgn0033093	CG3270	CG3270	-1.89	0.00
FBgn0038224	CG3321	CG3321	-1.89	0.00
FBgn0038516	CG5840	CG5840	-1.89	0.00
FBgn0030872	Ucp4A	Ucp4A	-1.89	0.00
FBgn0032393	CG12264	CG12264	-1.89	0.00
FBgn0036630	Aats-tyr	Tyrosyl-tRNA synthetase	-1.89	0.00
FBgn0051090	CG31090	CG31090	-1.89	0.00
FBgn0051029	CG31029	CG31029	-1.89	0.00
FBgn0025790	TBPH	TBPH	-1.89	0.00
FBgn0028970	betaggt-II	beta subunit of type II geranylgeranyl transferase	-1.90	0.00
FBgn0033913	CG8468	CG8468	-1.90	0.00
FBgn0034334	GstE10	Glutathione S transferase E10	-1.90	0.00
FBgn0010548	Aldh-III	Aldehyde dehydrogenase type III	-1.90	0.00
FBgn0035225	cue	cueball	-1.90	0.00
FBgn0033812	CG4663	CG4663	-1.90	0.00
FBgn0036702	CG6512	CG6512	-1.90	0.00
FBgn0036947	CG7306	CG7306	-1.90	0.00
FBgn0034885	CG4019	CG4019	-1.90	0.00
FBgn0037643	CG11963	CG11963	-1.90	0.00
FBgn0035586	CG10671	CG10671	-1.90	0.00
FBgn0035043	CG4781	CG4781	-1.91	0.00
FBgn0052541	CG32541	CG32541	-1.91	0.00
FBgn0032268	CG6138	CG6138	-1.91	0.00
FBgn0029591	CG14799	CG14799	-1.91	0.00
FBgn0036737	Jon74E	Jonah 74E	-1.91	0.00

FBgn0011638	La	La autoantigen-like	-1.92	0.00
FBgn0030615	Cyp4s3	Cyp4s3	-1.92	0.00
FBgn0031068	CG12534	CG12534	-1.92	0.00
FBgn0037763	CG16904	CG16904	-1.92	0.00
FBgn0038046	CG5641	CG5641	-1.92	0.00
FBgn0037129	CG14565	CG14565	-1.92	0.00
FBgn0010651	l(2)08717	lethal (2) 08717	-1.92	0.00
FBgn0052656	CG32656	CG32656	-1.92	0.00
FBgn0030746	CG9981	CG9981	-1.92	0.00
FBgn0035298	CG1140	CG1140	-1.92	0.00
FBgn0050502	CG30502	CG30502	-1.92	0.00
FBgn0051721	Trim9	Trim9	-1.92	0.00
FBgn0003738	Tpi	Triose phosphate isomerase	-1.92	0.00
FBgn0033189	Cyt-b5	Cyt-b5	-1.92	0.00
FBgn0038981	CG5346	CG5346	-1.93	0.00
FBgn0039719	CG15515	CG15515	-1.93	0.00
FBgn0003748	Treh	Trehalase	-1.93	0.00
FBgn0043471	kappaTry	kappaTry	-1.93	0.00
FBgn0016013	Faa	Fumarylacetoacetase	-1.93	0.00
FBgn0037750	CG12946	CG12946	-1.93	0.00
FBgn0011270	Pglym87	Pglym87	-1.93	0.00
FBgn0013949	Ela	Elastin-like	-1.93	0.00
FBgn0004551	Ca-P60A	Calcium ATPase at 60A	-1.93	0.00
FBgn0050159	CG30159	CG30159	-1.93	0.00
FBgn0015816	Slh	SLY-1 homologous	-1.93	0.00
FBgn0033717	CG8839	CG8839	-1.94	0.00
FBgn0033271	CG8708	CG8708	-1.94	0.00
FBgn0036857	CG9629	CG9629	-1.94	0.00
FBgn0033918	CG8531	CG8531	-1.94	0.00
FBgn0010551	l(2)03709	lethal (2) 03709	-1.94	0.00
FBgn0020545	kraken	kraken	-1.94	0.00
FBgn0031713	CG7277	CG7277	-1.94	0.00
FBgn0028670	Vha100-2	Vha100-2	-1.94	0.00
FBgn0021765	scu	scully	-1.94	0.00
FBgn0037769	CG12419	CG12419	-1.94	0.00
FBgn0033269	CG8709	CG8709	-1.94	0.00

FBgn0032340	CG6181	CG6181	-1.95	0.00
FBgn0028479	CG4389	CG4389	-1.95	0.00
FBgn0032889	CG9331	CG9331	-1.95	0.00
FBgn0041789	Pax	Paxillin	-1.95	0.00
FBgn0032384	CG14944	CG14944	-1.95	0.00
FBgn0037880	CG17726	CG17726	-1.95	0.00
FBgn0051265	CG31265	CG31265	-1.95	0.00
FBgn0039436	CG6478	CG6478	-1.96	0.00
FBgn0031024	l(1)G0156	lethal (1) G0156	-1.96	0.00
FBgn0030481	CG1662	CG1662	-1.96	0.00
FBgn0030442	CG15720	CG15720	-1.96	0.00
FBgn0016684	NaPi-T	Na[+]-dependent inorganic phosphate cotransporter	-1.96	0.00
FBgn0040279	Osi14	Osiris 14	-1.96	0.00
FBgn0028968	gammaCop	gamma-coatomer protein	-1.96	0.00
FBgn0034642	CG15674	CG15674	-1.96	0.00
FBgn0035241	CG12105	CG12105	-1.96	0.00
FBgn0030575	CG5321	CG5321	-1.97	0.00
FBgn0032881	CG9319	CG9319	-1.97	0.00
FBgn0028662	VhaPPA1-1	VhaPPA1-1	-1.97	0.00
FBgn0037225	CG14643	CG14643	-1.97	0.00
FBgn0031889	Pvf3	PDGF- and VEGF-related factor 3	-1.97	0.00
FBgn0031653	Jon25Biii	Jonah 25Biii	-1.97	0.00
FBgn0010497	l(2)01810	lethal (2) 01810	-1.97	0.00
FBgn0033949	CG10131	CG10131	-1.98	0.00
FBgn0051954	CG31954	CG31954	-1.98	0.00
FBgn0040256	Ugt86Dd	Ugt86Dd	-1.98	0.00
FBgn0033735	CG8525	CG8525	-1.98	0.00
FBgn0027792	l(1)G0431	lethal (1) G0431	-1.98	0.00
FBgn0051975	CG31975	CG31975	-1.98	0.00
FBgn0015834	Trip1	Trip1	-1.98	0.00
FBgn0039441	CG5476	CG5476	-1.98	0.00
FBgn0015010	Ag5r	Antigen 5-related	-1.98	0.00
FBgn0032379	Pde1c	Phosphodiesterase 1c	-1.98	0.00
FBgn0003071	Pfk	Phosphofructokinase	-1.98	0.00
FBgn0030777	CG9672	CG9672	-1.98	0.00
FBgn0032464	CG5075	CG5075	-1.98	0.00

FBgn0020906	Jon25Bi	Jonah 25Bi	-1.98	0.00
FBgn0037166	CG11426	CG11426	-1.98	0.00
FBgn0033448	CG1623	CG1623	-1.98	0.00
FBgn0033464	CG1441	CG1441	-1.99	0.00
FBgn0024978	CG2713	CG2713	-1.99	0.00
FBgn0032382	CG14935	CG14935	-1.99	0.00
FBgn0033690	CG8862	CG8862	-1.99	0.00
FBgn0000299	Cg25C	Collagen type IV	-1.99	0.00
FBgn0001091	Gapdh1	Glyceraldehyde 3 phosphate dehydrogenase 1	-1.99	0.00
FBgn0015247	Iap2	Inhibitor of apoptosis 2	-2.00	0.00
FBgn0034645	CG10320	CG10320	-2.00	0.00
FBgn0038347	CG18522	CG18522	-2.00	0.00
FBgn0004922	RpS6	Ribosomal protein S6	-2.00	0.00
FBgn0005670	Cyp4d1	Cytochrome P450-4d1	-2.00	0.00
FBgn0002570	LvpH	Larval visceral protein H	-2.00	0.00
FBgn0032271	CG7329	CG7329	-2.00	0.00
FBgn0036226	CG7252	CG7252	-2.00	0.00
FBgn0035858	CG13674	CG13674	-2.01	0.00
FBgn0040507	ACXD	ACXD	-2.01	0.00
FBgn0035664	Jon65Aiv	Jonah 65Aiv	-2.01	0.00
FBgn0037356	CG12170	CG12170	-2.01	0.00
FBgn0024361	Tsp2A	Tetraspanin 2A	-2.01	0.00
FBgn0035311	CR15821	CR15821	-2.01	0.00
FBgn0000487	Dox-A3	Diphenol oxidase A3	-2.01	0.00
FBgn0053071	CG33713	CG33713	-2.01	0.00
FBgn0035588	CG10672	CG10672	-2.02	0.00
FBgn0030990	CG7556	CG7556	-2.02	0.00
FBgn0035484	CG11594	CG11594	-2.02	0.00
FBgn0003162	Pu	Punch	-2.02	0.05
FBgn0038938	CG7084	CG7084	-2.02	0.00
FBgn0039223	CG5805	CG5805	-2.02	0.00
FBgn0010549	l(2)03659	lethal (2) 03659	-2.02	0.00
FBgn0031093	CG9581	CG9581	-2.02	0.00
FBgn0023540	CG3630	CG3630	-2.02	0.00
FBgn0037936	CG6908	CG6908	-2.02	0.00
FBgn0029721	l(1)G0334	lethal (1) G0334	-2.02	0.00

FBgn0035587	CG4623	CG4623	-2.02	0.00
FBgn0014869	Pglym78	Phosphoglyceromutase	-2.03	0.00
FBgn0032308	Porin2	Porin2	-2.03	0.00
FBgn0020018	Ppox	Protoporphyrinogen oxidase	-2.03	0.00
FBgn0017567	ND23	NADH:ubiquinone reductase 23kD subunit precursor	-2.03	0.00
FBgn0039252	CG11771	CG11771	-2.03	0.00
FBgn0019928	Ser8	Ser8	-2.03	0.00
FBgn0039311	CG10513	CG10513	-2.03	0.00
FBgn0035070	CG3650	CG3650	-2.04	0.00
FBgn0050035	CG30035	CG30035	-2.04	0.00
FBgn0000409	Cyt-c-p	Cytochrome c proximal	-2.04	0.00
FBgn0037389	CG10991	CG10991	-2.04	0.00
FBgn0035147	CG12030	CG12030	-2.04	0.00
FBgn0010516	wal	walrus	-2.05	0.00
FBgn0040321	GNBP3	Gram-negative bacteria binding protein 3	-2.05	0.00
FBgn0026409	Mpcp	Mitochondrial phosphate carrier protein	-2.05	0.00
FBgn0028938	CG16886	CG16886	-2.05	0.00
FBgn0000355	Cp15	Chorion protein 15	-2.05	0.00
FBgn0053161	CG32269	CG32269	-2.06	0.00
FBgn0033672	rho-7	rhomboid-7	-2.06	0.00
FBgn0035611	CG13285	CG13285	-2.06	0.00
FBgn0003450	snk	snake	-2.06	0.00
FBgn0037358	CG2185	CG2185	-2.06	0.00
FBgn0022709	Adk1	Adenylate kinase-1	-2.06	0.00
FBgn0025837	CG17636	CG17636	-2.07	0.00
FBgn0031380	Got2	Glutamate oxaloacetate transaminase 2	-2.07	0.00
FBgn0030759	CG13014	CG13014	-2.07	0.00
FBgn0011768	Fdh	Formaldehyde dehydrogenase	-2.07	0.00
FBgn0002569	LvpD	Larval visceral protein D	-2.08	0.00
FBgn0023477	Tal	Tal	-2.08	0.00
FBgn0032237	CG5362	CG5362	-2.08	0.00
FBgn0033294	CG8693	CG8693	-2.08	0.00
FBgn0004236	alphaTub67C	alpha-Tubulin at 67C	-2.08	0.00
FBgn0030980	CG7406	CG7406	-2.08	0.00
FBgn0031307	CG4726	CG4726	-2.09	0.00
FBgn0030992	CG33253	CG33253	-2.09	0.00

FBgn0020910	RpL3	Ribosomal protein L3	-2.09	0.00
FBgn0034716	Oatp58Dc	Organic anion transporting polypeptide 58Dc	-2.09	0.00
FBgn0032413	CG16997	CG16997	-2.09	0.00
FBgn0028569	robl37BC	robl37BC	-2.10	0.00
FBgn0038922	CG6439	CG6439	-2.11	0.00
FBgn0037298	CG2604	CG2604	-2.11	0.00
FBgn0029827	CG6048	CG6048	-2.11	0.00
FBgn0033787	CG13321	CG13321	-2.11	0.00
FBgn0031813	CG9527	CG9527	-2.11	0.00
FBgn0032412	CG16996	CG16996	-2.12	0.00
FBgn0037123	CG14569	CG14569	-2.12	0.00
FBgn0034141	CG8311	CG8311	-2.12	0.00
FBgn0035967	CG4641	CG4641	-2.12	0.00
FBgn0039143	CG5991	CG5991	-2.12	0.00
FBgn0034436	CG11961	CG11961	-2.12	0.00
FBgn0001995	mRpL4	mitochondrial ribosomal protein L4	-2.12	0.00
FBgn0017566	ND75	NADH:ubiquinone reductase 75kD subunit precursor	-2.12	0.00
FBgn0037724	Fst	Frost	-2.12	0.00
FBgn0032187	CG4839	CG4839	-2.12	0.00
FBgn0032312	CG14071	CG14071	-2.12	0.00
FBgn0020653	Trxr-1	Thioredoxin reductase-1	-2.13	0.00
FBgn0028945	CG7631	CG7631	-2.13	0.00
FBgn0030073	CG10962	CG10962	-2.13	0.00
FBgn0027610	CG8790	CG8790	-2.14	0.00
FBgn0003889	betaTub85D	beta-Tubulin at 85D	-2.14	0.00
FBgn0039857	RpL6	Ribosomal protein L6	-2.14	0.00
FBgn0033317	CG8635	CG8635	-2.14	0.00
FBgn0024891	ferrochelatase	ferrochelatase	-2.15	0.00
FBgn0039777	Jon99Fii	Jonah 99Fii	-2.15	0.00
FBgn0004465	Su(P)	Suppressor of ref(2)P sterility	-2.15	0.00
FBgn0051302	CG31302	CG31302	-2.15	0.00
FBgn0031544	CG17593	CG17593	-2.15	0.00
FBgn0000579	Eno	Enolase	-2.15	0.00
FBgn0033393	CG8029	CG8029	-2.15	0.00
FBgn0033999	CG8093	CG8093	-2.16	0.00
FBgn0035788	CG8541	CG8541	-2.16	0.00

FBgn0026872	CG14777	CG14777	-2.16	0.00
FBgn0020508	Ag5r2	Antigen 5-related 2	-2.16	0.00
FBgn0033644	CG8234	CG8234	-2.16	0.00
FBgn0003980	Vm26Ab	Vitelline membrane 26Ab	-2.16	0.00
FBgn0033296	CG11669	CG11669	-2.16	0.00
FBgn0026314	Ugt35b	UDP-glycosyltransferase 35b	-2.16	0.00
FBgn0037797	CG12420	CG12420	-2.16	0.00
FBgn0036663	CG9674	CG9674	-2.17	0.00
FBgn0003888	betaTub60D	beta-Tubulin at 60D	-2.17	0.00
FBgn0031497	CG17259	CG17259	-2.17	0.00
FBgn0038466	CG8907	CG8907	-2.17	0.00
FBgn0034417	CG15117	CG15117	-2.17	0.00
FBgn0035195	Sac1	Sac1	-2.18	0.00
FBgn0016031	lama	lamina ancestor	-2.18	0.00
FBgn0037024	CG4365	CG4365	-2.18	0.00
FBgn0003887	betaTub56D	beta-Tubulin at 56D	-2.18	0.00
FBgn0052600	dpr8	dpr8	-2.18	0.00
FBgn0033048	CG7881	CG7881	-2.18	0.00
FBgn0002723	Rst(1)JH	Resistance to Juvenile Hormone	-2.18	0.00
FBgn0024939	RpL8	Ribosomal protein L8	-2.18	0.00
FBgn0032177	CG33301	CG33301	-2.19	0.00
FBgn0050493	CG30493	CG30493	-2.19	0.00
FBgn0037163	CG11440	CG11440	-2.19	0.00
FBgn0039435	CG14240	CG14240	-2.19	0.00
FBgn0032024	CG14273	CG14273	-2.19	0.00
FBgn0034783	CG9825	CG9825	-2.20	0.00
FBgn0030731	CG3415	CG3415	-2.20	0.00
FBgn0032066	CG9463	CG9463	-2.21	0.01
FBgn0051463	CG31463	CG31463	-2.21	0.00
FBgn0035359	CG1143	CG1143	-2.21	0.00
FBgn0036639	CG4229	CG4229	-2.21	0.00
FBgn0030347	CG15739	CG15739	-2.22	0.00
FBgn0024315	Picot	Picot	-2.22	0.00
FBgn0019624	CoVa	Cytochrome c oxidase subunit Va	-2.22	0.00
FBgn0016691	Oscp	Oligomycin sensitivity-conferring protein	-2.22	0.00
FBgn0032222	CG5037	CG5037	-2.22	0.00

FBgn0031660	mRpL28	mitochondrial ribosomal protein L28	-2.23	0.00
FBgn0025640	CG13369	CG13369	-2.23	0.00
FBgn0038038	CG5167	CG5167	-2.23	0.00
FBgn0035667	Jon65Ai	Jonah 65Ai	-2.24	0.00
FBgn0001128	Gpdh	Glycerol 3 phosphate dehydrogenase	-2.24	0.00
FBgn0036682	Nc73EF	Neural conserved at 73EF	-2.24	0.00
FBgn0036597	CG4962	CG4962	-2.24	0.00
FBgn0023537	CG17896	CG17896	-2.24	0.00
FBgn0036227	CG17826	CG17826	-2.24	0.00
FBgn0032833	CG10664	CG10664	-2.25	0.00
FBgn0037718	CG8286	CG8286	-2.25	0.00
FBgn0003965	v	vermilion	-2.26	0.00
FBgn0034295	CG10911	CG10911	-2.26	0.00
FBgn0010609	l(2)44DEa	lethal (2) 44DEa	-2.26	0.00
FBgn0035780	CG18417	CG18417	-2.26	0.00
FBgn0039873	CG2191	CG2191	-2.26	0.00
FBgn0038646	CG7715	CG7715	-2.27	0.00
FBgn0020655	Gap69C	GTPase-activating protein 69C	-2.27	0.00
FBgn0014026	RpL7A	Ribosomal protein L7A	-2.27	0.00
FBgn0039045	CG17119	CG17119	-2.27	0.00
FBgn0010434	cora	coracle	-2.27	0.00
FBgn0033382	CG8058	CG8058	-2.27	0.00
FBgn0037801	CG3999	CG3999	-2.27	0.00
FBgn0039612	CG14523	CG14523	-2.28	0.00
FBgn0020509	Acp62F	Accessory gland peptide 62F	-2.28	0.00
FBgn0037125	CG14573	CG14573	-2.29	0.00
FBgn0001098	Gdh	Glutamate dehydrogenase	-2.29	0.00
FBgn0001187	Hex-C	Hexokinase C	-2.29	0.00
FBgn0004167	kst	karst	-2.29	0.00
FBgn0004780	Ccp84Ad	Ccp84Ad	-2.29	0.00
FBgn0041630	Hexo1	Hexosaminidase 1	-2.29	0.00
FBgn0020368	Vha68-1	Vha68-1	-2.30	0.00
FBgn0034553	CG9993	CG9993	-2.31	0.00
FBgn0035254	CG7974	CG7974	-2.31	0.00
FBgn0033903	CG8323	CG8323	-2.31	0.00
FBgn0034629	Acox57D-d	acyl-Coenzyme A oxidase at 57D distal	-2.32	0.00

FBgn0035023	itp	ion transport peptide	-2.32	0.00
FBgn0031706	mid	midline	-2.33	0.00
FBgn0027525	CG7686	CG7686	-2.33	0.00
FBgn0037131	CG14564	CG14564	-2.33	0.00
FBgn0038658	CG14292	CG14292	-2.34	0.00
FBgn0046114	Gclm	Glutamate-cysteine ligase modifier subunit	-2.34	0.00
FBgn0026721	fat-spondin	fat-spondin	-2.34	0.00
FBgn0035552	CG11350	CG11350	-2.34	0.00
FBgn0037873	CG6666	CG6666	-2.35	0.00
FBgn0039440	CG5471	CG5471	-2.35	0.00
FBgn0034711	CG3290	CG3290	-2.35	0.00
FBgn0032069	CG9468	CG9468	-2.35	0.00
FBgn0036949	CG7290	CG7290	-2.36	0.00
FBgn0036234	CG17824	CG17824	-2.36	0.00
FBgn0052473	CG32473	CG32473	-2.36	0.00
FBgn0032754	CG10700	CG10700	-2.36	0.00
FBgn0033883	CG16935	CG16935	-2.37	0.00
FBgn0027571	CG3523	CG3523	-2.37	0.00
FBgn0031940	CG7214	CG7214	-2.37	0.00
FBgn0032114	Aldh	Aldehyde dehydrogenase	-2.37	0.00
FBgn0016120	ATPsyn-d	ATP synthase, subunit d	-2.37	0.00
FBgn0036321	CG14120	CG14120	-2.37	0.00
FBgn0034432	CG7461	CG7461	-2.38	0.00
FBgn0020618	Rack1	Receptor of activated protein kinase C 1	-2.38	0.00
FBgn0019982	Gs1l	GS1-like	-2.38	0.00
FBgn0014903	CG14630	CG14630	-2.38	0.00
FBgn0034576	CG9350	CG9350	-2.39	0.00
FBgn0033101	CG9436	CG9436	-2.39	0.00
FBgn0026562	BM-40-SPARC	BM-40-SPARC	-2.39	0.00
FBgn0004888	Scsalpha	Succinyl coenzyme A synthetase alpha subunit	-2.39	0.00
FBgn0037715	CG9399	CG9399	-2.39	0.00
FBgn0033814	CG4670	CG4670	-2.40	0.00
FBgn0037387	CG1213	CG1213	-2.40	0.00
FBgn0040227	eIF-3p66	Eukaryotic initiation factor 3 p66 subunit	-2.40	0.00
FBgn0039537	CG5590	CG5590	-2.40	0.00
FBgn0036233	CG6947	CG6947	-2.40	0.00

FBgn0010611	Hmgs	HMG Coenzyme A synthase	-2.41	0.00
FBgn0037135	CG7414	CG7414	-2.42	0.00
FBgn0039434	CG5468	CG5468	-2.42	0.00
FBgn0033879	CG6543	CG6543	-2.42	0.00
FBgn0031206	CG12466	CG12466	-2.42	0.00
FBgn0035779	CG8562	CG8562	-2.42	0.00
FBgn0027868	Nup170	Nup170	-2.43	0.00
FBgn0030478	CG1640	CG1640	-2.43	0.00
FBgn0028983	Spn6	Serine protease inhibitor 6	-2.43	0.00
FBgn0033235	CG8728	CG8728	-2.43	0.00
FBgn0025117	und	uninitiated	-2.43	0.00
FBgn0020764	Alas	Aminolevulinate synthase	-2.44	0.00
FBgn0037955	CG6950	CG6950	-2.44	0.00
FBgn0030218	CG1628	CG1628	-2.44	0.00
FBgn0033229	CG12822	CG12822	-2.44	0.00
FBgn0035404	CG12079	CG12079	-2.44	0.00
FBgn0038135	CG8773	CG8773	-2.44	0.00
FBgn0032292	CG6750	CG6750	-2.45	0.00
FBgn0004782	Ccp84Ab	Ccp84Ab	-2.45	0.00
FBgn0037714	CG9396	CG9396	-2.45	0.00
FBgn0039774	CDase	Ceramidase	-2.45	0.00
FBgn0024992	CG2658	CG2658	-2.47	0.00
FBgn0051063	CG31063	CG31063	-2.47	0.00
FBgn0031069	CG12703	CG12703	-2.48	0.00
FBgn0026593	CG5707	CG5707	-2.48	0.00
FBgn0030932	CG6461	CG6461	-2.49	0.00
FBgn0014455	Ahcy13	Adenosylhomocysteinase at 13	-2.49	0.00
FBgn0000100	RpLP0	Ribosomal protein LP0	-2.49	0.00
FBgn0039769	CG15534	CG15534	-2.49	0.00
FBgn0039349	CG4685	CG4685	-2.49	0.00
FBgn0039886	CG2003	CG2003	-2.49	0.00
FBgn0019644	ATPsyn-b	ATP synthase, subunit b	-2.49	0.00
FBgn0033904	CG18327	CG18327	-2.49	0.00
FBgn0033703	CG13170	CG13170	-2.50	0.00
FBgn0010295	ng3	new glue 3	-2.51	0.00
FBgn0020236	ATPCL	ATP citrate lyase	-2.51	0.00

FBgn0039760	CG9682	CG9682	-2.51	0.00
FBgn0030853	CG5703	CG5703	-2.51	0.00
FBgn0037146	CG7470	CG7470	-2.51	0.00
FBgn0028533	CG7953	CG7953	-2.52	0.00
FBgn0021872	Xbp1	X box binding protein-1	-2.53	0.00
FBgn0038645	CG7714	CG7714	-2.53	0.00
FBgn0039849	CG11334	CG11334	-2.53	0.00
FBgn0033367	CG8193	CG8193	-2.54	0.00
FBgn0015623	Cpr	Cytochrome P450 reductase	-2.54	0.00
FBgn0004654	Pgd	Phosphogluconate dehydrogenase	-2.54	0.00
FBgn0003886	alphaTub85E	alpha-Tubulin at 85E	-2.54	0.00
FBgn0033765	nemy	no extended memory	-2.54	0.00
FBgn0010357	betaTry	betaTrypsin	-2.54	0.00
FBgn0030993	Mec2	Mec2	-2.55	0.00
FBgn0001220	Hsc70-5	Heat shock protein cognate 5	-2.55	0.00
FBgn0034582	CG10531	CG10531	-2.55	0.00
FBgn0034588	CG9394	CG9394	-2.55	0.00
FBgn0037517	CG10086	CG10086	-2.56	0.00
FBgn0040364	CG11378	CG11378	-2.56	0.00
FBgn0038986	CG5278	CG5278	-2.56	0.00
FBgn0039453	CG6403	CG6403	-2.58	0.00
FBgn0033184	CG12736	CG12736	-2.58	0.00
FBgn0003517	sta	stubarista	-2.58	0.00
FBgn0032779	CG16771	CG16771	-2.59	0.00
FBgn0028534	CG7916	CG7916	-2.59	0.00
FBgn0037891	CG5214	CG5214	-2.60	0.00
FBgn0032144	CG17633	CG17633	-2.60	0.00
FBgn0025620	CG13360	CG13360	-2.61	0.00
FBgn0024957	Irp-1B	Iron regulatory protein 1B	-2.61	0.00
FBgn0029969	CG10932	CG10932	-2.62	0.00
FBgn0032407	CG5325	CG5325	-2.62	0.00
FBgn0039697	CG7834	CG7834	-2.63	0.00
FBgn0003884	alphaTub84B	alpha-Tubulin at 84B	-2.63	0.00
FBgn0028948	CG15253	CG15253	-2.63	0.00
FBgn0020235	ATPsyn-gamma	ATP synthase-gamma chain	-2.63	0.00
FBgn0010425	epsilonTry	epsilonTrypsin	-2.63	0.00

FBgn0010213	Sod2	Superoxide dismutase 2 (Mn)	-2.63	0.00
FBgn0029889	l(1)G0255	lethal (1) G0255	-2.63	0.00
FBgn0040349	CG3699	CG3699	-2.64	0.00
FBgn0032511	CG9306	CG9306	-2.64	0.00
FBgn0039609	CG14529	CG14529	-2.65	0.00
FBgn0030400	CG11138	CG11138	-2.66	0.00
FBgn0034497	CG9090	CG9090	-2.66	0.00
FBgn0036551	CG17029	CG17029	-2.66	0.00
FBgn0003885	alphaTub84D	alpha-Tubulin at 84D	-2.66	0.00
FBgn0005671	Vha55	Vacuolar H[+]-ATPase 55kD B subunit	-2.68	0.00
FBgn0035887	Jon66Cii	Jonah 66Cii	-2.68	0.00
FBgn0040336	CG9904	CG9904	-2.68	0.00
FBgn0033047	CG7882	CG7882	-2.68	0.00
FBgn0029807	CG3108	CG3108	-2.69	0.00
FBgn0035619	CG10592	CG10592	-2.69	0.00
FBgn0035360	CG1246	CG1246	-2.69	0.00
FBgn0031912	CG5261	CG5261	-2.70	0.00
FBgn0039736	CG7912	CG7912	-2.73	0.00
FBgn0031801	CG9498	CG9498	-2.73	0.00
FBgn0029890	CG4095	CG4095	-2.74	0.00
FBgn0034490	CG9864	CG9864	-2.75	0.00
FBgn0033226	CG1882	CG1882	-2.77	0.00
FBgn0014028	SdhB	Succinate dehydrogenase B	-2.78	0.00
FBgn0035343	CG16762	CG16762	-2.78	0.00
FBgn0039438	CG6452	CG6452	-2.80	0.00
FBgn0035670	CG10472	CG10472	-2.80	0.00
FBgn0035770	pst	pastrel	-2.80	0.00
FBgn0039471	CG6295	CG6295	-2.80	0.00
FBgn0037699	CG8147	CG8147	-2.81	0.00
FBgn0032373	CG12602	CG12602	-2.81	0.00
FBgn0016123	Aph-4	Alkaline phosphatase 4	-2.81	0.00
FBgn0020367	Vha68-2	Vha68-2	-2.82	0.00
FBgn0039737	CG7920	CG7920	-2.83	0.00
FBgn0019957	ND42	NADH:ubiquinone reductase 42kD subunit precursor	-2.84	0.00
FBgn0036023	CG18179	CG18179	-2.84	0.00
FBgn0029869	l(1)G0030	lethal (1) G0030	-2.85	0.00

FBgn0035513	CG1259	CG1259	-2.86	0.00
FBgn0033760	CG8785	CG8785	-2.86	0.00
FBgn0003863	alphaTry	alphaTrypsin	-2.87	0.00
FBgn0021906	RFeSP	Rieske iron-sulfur protein	-2.93	0.00
FBgn0034294	CG5765	CG5765	-2.94	0.00
FBgn0034479	CG8654	CG8654	-2.95	0.00
FBgn0034388	CG15092	CG15092	-2.96	0.00
FBgn0039439	CG6460	CG6460	-2.96	0.00
FBgn0043470	lambdaTry	lambdaTry	-2.97	0.00
FBgn0032947	CG17571	CG17571	-2.97	0.00
FBgn0038925	CG6022	CG6022	-2.99	0.00
FBgn0033465	CG12140	CG12140	-2.99	0.00
FBgn0034712	CG3264	CG3264	-3.00	0.00
FBgn0036622	CG4753	CG4753	-3.01	0.00
FBgn0038271	CG3731	CG3731	-3.01	0.00
FBgn0030776	CG4653	CG4653	-3.02	0.00
FBgn0039756	CG9743	CG9743	-3.02	0.00
FBgn0035582	CG13705	CG13705	-3.02	0.00
FBgn0030775	CG9673	CG9673	-3.04	0.00
FBgn0038136	CG8774	CG8774	-3.04	0.00
FBgn0033319	Jon44E	Jonah 44E	-3.05	0.00
FBgn0050457	CG30457	CG30457	-3.06	0.00
FBgn0036367	CG10116	CG10116	-3.06	0.00
FBgn0037204	CG11131	CG11131	-3.07	0.00
FBgn0036762	CG7430	CG7430	-3.08	0.00
FBgn0051410	CG31410	CG31410	-3.08	0.00
FBgn0036950	CG6996	CG6996	-3.08	0.00
FBgn0015032	Cyp4c3	Cytochrome P450-4c3	-3.09	0.00
FBgn0052237	CG32237	CG32237	-3.10	0.00
FBgn0024556	EfTuM	Elongation factor Tu mitochondrial	-3.11	0.00
FBgn0051305	CG6782	CG6782	-3.11	0.00
FBgn0031741	CG11034	CG11034	-3.12	0.00
FBgn0019960	CG6455	CG6455	-3.12	0.00
FBgn0036024	CG18180	CG18180	-3.12	0.00
FBgn0019830	colt	congested-like trachea	-3.14	0.00
FBgn0028583	lcs	la costa	-3.15	0.00

FBgn0034710	CG3292	CG3292	-3.16	0.00
FBgn0015568	alpha-Est1	alpha-Esterase-1	-3.18	0.00
FBgn0002719	Men	Malic enzyme	-3.20	0.00
FBgn0030999	CG7874	CG7874	-3.20	0.00
FBgn0030264	CG1961	CG1961	-3.23	0.00
FBgn0039635	CG11876	CG11876	-3.23	0.00
FBgn0052564	CG32564	CG32564	-3.28	0.00
FBgn0036948	CG7298	CG7298	-3.30	0.00
FBgn0033720	CG13160	CG13160	-3.30	0.00
FBgn0028473	CG8801	CG8801	-3.33	0.00
FBgn0038400	CG5903	CG5903	-3.37	0.00
FBgn0024293	Spn43Ab	Serine protease inhibitor 43Ab	-3.37	0.00
FBgn0036951	CG7017	CG7017	-3.40	0.00
FBgn0036953	CG17145	CG17145	-3.42	0.00
FBgn0037001	CG6020	CG6020	-3.45	0.00
FBgn0040363	CG11384	CG11384	-3.46	0.00
FBgn0053138	CG33138	CG33138	-3.47	0.00
FBgn0030668	CG8128	CG8128	-3.48	0.00
FBgn0002571	LvpL	Larval visceral protein L	-3.50	0.00
FBgn0033604	CG9070	CG9070	-3.61	0.00
FBgn0011556	zetaTry	zetaTrypsin	-3.63	0.00
FBgn0052296	Mrtf	Myocardin-related transcription factor	-3.63	0.00
FBgn0031533	CG2772	CG2772	-3.65	0.00
FBgn0012034	AcCoAS	Acetyl Coenzyme A synthase	-3.71	0.00
FBgn0035600	CG4769	CG4769	-3.72	0.00
FBgn0032068	CG9466	CG9466	-3.75	0.00
FBgn0028949	CG15254	CG15254	-3.75	0.00
FBgn0033782	sug	sugarbabe	-3.83	0.00
FBgn0000473	Cyp6a2	Cytochrome P450-6a2	-3.86	0.00
FBgn0034390	CG15093	CG15093	-3.98	0.00
FBgn0031771	CG9140	CG9140	-4.04	0.00
FBgn0016078	wun	wunen	-4.06	0.00
FBgn0023507	CG3835	CG3835	-4.08	0.00
FBgn0036831	CG6839	CG6839	-4.11	0.00
FBgn0030774	CG9675	CG9675	-4.11	0.00
FBgn0031654	Jon25Bii	Jonah 25Bii	-4.11	0.00

FBgn0022160	l(2)k05713	lethal (2) k05713	-4.22	0.00
FBgn0036642	CG4169	CG4169	-4.24	0.00
FBgn0034247	CG6484	CG6484	-4.24	0.00
FBgn0030148	CG3106	CG3106	-4.34	0.00
FBgn0035781	CG8560	CG8560	-4.41	0.00
FBgn0031538	CG3246	CG3246	-4.74	0.00
FBgn0035006	CG4563	CG4563	-4.76	0.00
FBgn0003149	Prm	Paramyosin	-4.77	0.00
FBgn0033423	CG1809	CG1809	-4.83	0.00
FBgn0033327	PGRP-SC1b	PGRP-SC1b	-5.00	0.00
FBgn0033079	Fmo-2	Flavin-containing monooxygenase 2	-5.10	0.00
FBgn0046878	Obp83cd	Odorant-binding protein 83cd	-5.18	0.00
FBgn0037996	CG4830	CG4830	-5.49	0.00
FBgn0033830	CG10814	CG10814	-6.57	0.00
FBgn0037230	CG9780	CG9780	-6.67	0.00
FBgn0033246	CG11198	CG11198	-6.92	0.00
