# Supplementary material for: Mechanisms Underlying Hypoxia Tolerance in Drosophila melanogaster: hairy as a Metabolic Switch
Source: PLoS Genet. 2008 Oct 17;4(10):e1000221. doi: 10.1371/journal.pgen.1000221 (PMC2556400; doi:10.1371/journal.pgen.1000221)
Supplement: Table S2 — List of Significantly Altered Genes in Hypoxia-Selected Drosophila melanogaster at Adult Stage. (0.08 MB PDF) [file pgen.1000221.s003.pdf]

Table S2. List of Significantly Altered Genes in Hypoxia-Selected *Drosophila melanogaster* at Adult Stage

Flybase ID	Symbol	Full_name	Fold Change	q-value(%)
FBgn0041579	AttC	Attacin C	4.85	0.00
FBgn0034317	CR14499	CR14499	4.35	0.00
FBgn0036068	CG6640	CG6640	3.54	0.00
FBgn0030929	CG15043	CG15043	3.08	0.00
FBgn0038346	CG14872	CG14872	3.06	0.00
FBgn0035994	CG14179	CG14179	2.96	0.00
FBgn0033788	CG13323	CG13323	2.90	0.00
FBgn0040957	CG14272	CG14272	2.68	0.00
FBgn0033659	Damm	Death associated molecule related to Mch2	2.67	0.00
FBgn0035176	CG13905	CG13905	2.53	0.00
FBgn0033789	CG13324	CG13324	2.52	0.00
FBgn0041581	AttB	Attacin B	2.49	0.00
FBgn0033857	CG13335	CG13335	2.44	0.00
FBgn0035868	CG7194	CG7194	2.38	0.00
FBgn0004427	LysD	Lysozyme D	2.37	0.00
FBgn0032493	CG15479	CG15479	2.36	0.00
FBgn0033301	CG12780	CG12780	2.36	0.00
FBgn0040923	CG11368	CG11368	2.35	0.00
FBgn0012042	AttA	attacin A	2.32	0.00
FBgn0034407	DptB	Diptericin B	2.28	0.00
FBgn0025692	CG3814	CG3814	2.26	0.00
FBgn0036232	CG14125	CG14125	2.24	0.00
FBgn0037562	CG11671	CG11671	2.23	0.00
FBgn0010385	Def	defensin	2.22	0.00
FBgn0031176	CG1678	CG1678	2.20	0.00
FBgn0033787	CG13321	CG13321	2.19	0.00
FBgn0030262	Vago	Vago	2.16	0.00
FBgn0034160	CG5550	CG5550	2.14	0.00
FBgn0038353	CG5399	CG5399	2.12	0.00
FBgn0028583	lcs	la costa	2.10	0.00
FBgn0034294	CG5765	CG5765	2.05	0.00
FBgn0036951	CG7017	CG7017	2.05	0.00
FBgn0030040	CG15347	CG15347	2.04	0.00
FBgn0043578	PGRP-SB1	PGRP-SB1	2.03	0.00

FBgn0024361	Tsp2A	Tetraspanin 2A	1.99	0.00
FBgn0031489	CG17224	CG17224	1.93	0.00
FBgn0035743	CG15829	CG15829	1.91	0.00
FBgn0038074	CG6188	CG6188	1.90	0.00
FBgn0034758	CG13510	CG13510	1.89	0.00
FBgn0033560	CG30015	CG30015	1.89	0.00
FBgn0033173	CG30502	CG30502	1.89	0.00
FBgn0034871	CG3906	CG3906	1.87	0.00
FBgn0031033	CG14219	CG14219	1.86	0.00
FBgn0038292	CG3987	CG3987	1.84	0.00
FBgn0034331	CG15067	CG15067	1.84	0.00
FBgn0040887	CG33254	CG33254	1.82	0.00
FBgn0036833	CG3819	CG3819	1.80	0.00
FBgn0039756	CG9743	CG9743	1.79	0.00
FBgn0025454	Cyp6g1	Cyp6g1	1.79	0.00
FBgn0040832	CG8012	CG8012	1.78	0.00
FBgn0036203	CG6004	CG6004	1.77	0.00
FBgn0028932	CG16890	CG16890	1.73	0.00
FBgn0033124	Tsp42Ec	Tetraspanin 42Ec	1.72	0.00
FBgn0020416	Idgf1	Imaginal disc growth factor1	1.70	0.00
FBgn0031021	CG12203	CG12203	1.68	0.00
FBgn0036945	CG6981	CG6981	1.67	0.00
FBgn0028940	Cyp28a5	Cyp28a5	1.67	0.00
FBgn0004240	Dpt	Diptericin	1.66	0.00
FBgn0039109	CG10365	CG10365	1.65	0.00
FBgn0015010	Ag5r	antigen 5-related	1.65	0.00
FBgn0038957	CG7059	CG7059	1.64	0.00
FBgn0027562	CG10345	CG10345	1.63	0.00
FBgn0015772	Nak	Numb-associated kinase	1.63	0.00
FBgn0039774	CDase	Ceramidase	1.62	0.00
FBgn0030487	CG12726	CG12726	1.62	0.00
FBgn0037809	CG12818	CG12818	1.60	0.00
FBgn0002733	HLHmbeta	E(spl) region transcript mbeta	1.60	0.00
FBgn0032836	CG10680	CG10680	1.60	0.00
FBgn0035770	pst	pastrel	1.60	0.00
FBgn0032202	CG18619	CG18619	1.59	0.00
FBgn0003961	Uro	Urate oxidase	1.59	0.00

FBgn0031830	CG11015	CG11015	1.58	0.00
FBgn0038198	CG3153	CG3153	1.58	0.00
FBgn0013307	Odc1	ornithine decarboxylase	1.57	0.00
FBgn0038294	Zeelin1	Zeelin1	1.56	0.00
FBgn0022355	Tsf1	Transferrin	1.56	0.00
FBgn0034144	CG5089	CG5089	1.56	0.00
FBgn0003274	RpLP2	Ribosomal protein LP2	1.55	0.00
FBgn0040582	CG5791	CG5791	1.55	0.00
FBgn0039319	CG13659	CG13659	1.55	0.00
FBgn0027785	NP15.6	NP15.6	1.54	0.00
FBgn0030304	Cyp4g15	Cyp4g15	1.54	0.00
FBgn0020618	Rack1	Receptor of activated protein kinase C 1	1.54	0.00
FBgn0036433	CG9628	CG9628	1.54	0.00
FBgn0031502	CG3524	CG3524	1.53	0.00
FBgn0033953	CG12861	CG12861	1.53	0.00
FBgn0040993	CG17325	CG17325	1.53	0.00
FBgn0028381	decay	caspase-3 protein	1.52	0.00
FBgn0034128	CG4409	CG4409	1.52	0.00
FBgn0034538	CG16799	CG16799	1.52	0.00
FBgn0040735	CG16836	CG16836	1.51	0.00
FBgn0014184	Oda	gut feeling	1.51	0.00
FBgn0028542	CG33115	CG33115	1.50	0.00
FBgn0027525	CG7686	CG7686	1.50	0.00
FBgn0023549	Mct1	Monocarboxylate transporter 1	1.50	0.00
FBgn0031769	CG9135	CG9135	-1.51	0.00
FBgn0031057	CG14224	CG14224	-1.54	0.00
FBgn0030377	CG1924	CG1924	-1.54	0.00
FBgn0033361	CG8181	CG8181	-1.54	0.00
FBgn0015283	Pros54	Proteasome 54kD subunit	-1.54	0.00
FBgn0034629	Acox57D-d	acyl-Coenzyme A oxidase at 57D distal	-1.54	0.00
FBgn0010247	Parp	Poly-(ADP-ribose) polymerase	-1.55	0.00
FBgn0027505	rab3-GAP	rab3-GAP	-1.56	0.00
FBgn0034143	CG8303	CG8303	-1.56	0.00
FBgn0039257	tnc	tenectin	-1.58	0.00
FBgn0003717	Tl	toll / Toll	-1.58	0.00
FBgn0000427	dec-1	Defective chorion 1	-1.59	0.00
FBgn0036549	CG10516	CG10516	-1.59	0.00

FBgn0035802	CG33275	CG33275	-1.61	0.00
FBgn0033718	CG13162	CG13162	-1.61	0.00
FBgn0020517	Skeletor	Skeletor	-1.61	0.00
FBgn0013343	Syx1A	Syntaxin1A	-1.62	0.00
FBgn0029004	lmg	lemming	-1.64	0.00
FBgn0033816	CG4679	CG4679	-1.65	0.00
FBgn0024289	Sodh-1	Sorbitol dehydrogenase 1	-1.67	0.00
FBgn0031754	CG13997	CG13997	-1.67	0.00
FBgn0039891	CG31998	CG31998	-1.68	0.00
FBgn0039473	CG17191	CG17191	-1.68	0.00
FBgn0014076	Vm32E	Vitelline membrane 32E	-1.72	0.00
FBgn0001225	Hsp26	heat shock protein hsp26	-1.74	0.00
FBgn0038611	CG14309	CG14309	-1.75	0.00
FBgn0003358	Jon99Ci	Jonah 99Ci	-1.75	0.00
FBgn0039181	BRWD3	BRWD3	-1.78	0.00
FBgn0036996	CG5932	CG5932	-1.79	0.00
FBgn0032127	CG13114	CG13114	-1.80	0.00
FBgn0002562	Lsp1alpha	Larval serum protein 1 alpha	-1.81	0.00
FBgn0031342	CG31661	CG31661	-1.85	0.00
FBgn0037763	CG16904	CG16904	-1.88	0.00
FBgn0038914	fit	female-specific independent of transformer	-1.93	0.00
FBgn0039945	CG17159	CG17159	-2.00	0.00
FBgn0032736	CG31792/CG31793	CG31792/CG31793	-2.03	0.00
FBgn0020906	Jon25Bi	Jonah 25B / Jonah 25Bi / Serine protease 4	-2.04	0.00
FBgn0039471	CG6295	CG6295	-2.05	0.00
FBgn0031654	Jon25Bii	Jonah 25B / Jonah 25Bii	-2.12	0.00
FBgn0031653	Jon25Biii	Jonah 25B / Jonah 25Biii	-2.24	0.00
FBgn0032249	CG5337	CG5337	-2.29	0.00
FBgn0017424	CG11538	CG11538	-2.88	0.00
FBgn0039472	CG17192	CG17192	-2.97	0.00
