# Supplementary material for: Mechanisms Underlying Hypoxia Tolerance in Drosophila melanogaster: hairy as a Metabolic Switch
Source: PLoS Genet. 2008 Oct 17;4(10):e1000221. doi: 10.1371/journal.pgen.1000221 (PMC2556400; doi:10.1371/journal.pgen.1000221)
Supplement: Table S3 — List of Significantly Altered Biological Processes in Hypoxia-Selected Drosophila melanogaster at Larval Stage. (0.18 MB PDF) [file pgen.1000221.s004.pdf]

Table S3. List of Significantly Altered Biological Processes in Hypoxia-Selected *Drosophila melanogaster* at Larval Stage

GOID	GO Name	Number Changed	Number Measured	Number in GO	% Changed	% Present	p value
35159	regulation of tracheal tube length	4	4	7	100.0	57.143	0.009
7599	hemostasis	3	3	7	100.0	42.857	0.009
6106	fumarate metabolism	3	3	5	100.0	60.000	0.010
35046	pronuclear migration	3	3	10	100.0	30.000	0.011
6559	L-phenylalanine catabolism	3	3	5	100.0	60.000	0.014
6572	tyrosine catabolism	3	3	3	100.0	100.000	0.014
6558	L-phenylalanine metabolism	3	3	5	100.0	60.000	0.014
9074	aromatic amino acid family catabolism	3	3	5	100.0	60.000	0.014
6573	valine metabolism	3	3	8	100.0	37.500	0.020
6542	glutamine biosynthesis	2	2	4	100.0	50.000	0.052
48052	R1/R6 cell differentiation (sensu Endopterygota)	2	2	3	100.0	66.667	0.056
7462	R1/R6 cell fate commitment	2	2	3	100.0	66.667	0.056
50817	coagulation	2	2	3	100.0	66.667	0.057
7295	egg chamber growth (sensu Insecta)	2	2	3	100.0	66.667	0.057
9618	response to pathogenic bacteria	2	2	5	100.0	40.000	0.058
42829	defense response to pathogen	2	2	5	100.0	40.000	0.058
42828	response to pathogen	2	2	5	100.0	40.000	0.058
42830	defense response to pathogenic bacteria	2	2	5	100.0	40.000	0.058
6568	tryptophan metabolism	2	2	10	100.0	20.000	0.058
18195	peptidyl-arginine modification	2	2	3	100.0	66.667	0.060
19919	peptidyl-arginine methylation\, to asymmetrical-dimethyl arginine	2	2	3	100.0	66.667	0.060
18216	peptidyl-arginine methylation	2	2	3	100.0	66.667	0.060
8359	regulation of bicoid mRNA localization	2	2	2	100.0	100.000	0.060
35246	peptidyl-arginine N-methylation	2	2	3	100.0	66.667	0.060
35247	peptidyl-arginine omega-N-methylation	2	2	3	100.0	66.667	0.060
6856	eye pigment precursor transport	2	2	2	100.0	100.000	0.061
6433	prolyl-tRNA aminoacylation	2	2	4	100.0	50.000	0.062
51084	posttranslational protein folding	2	2	5	100.0	40.000	0.063
35044	sperm aster formation	2	2	3	100.0	66.667	0.064
42417	dopamine metabolism	2	2	7	100.0	28.571	0.066
42542	response to hydrogen peroxide	2	2	2	100.0	100.000	0.068
302	response to reactive oxygen species	2	2	2	100.0	100.000	0.068
42744	hydrogen peroxide catabolism	2	2	2	100.0	100.000	0.068

6750	glutathione biosynthesis	2	2	7	100.0	28.571	0.068
42743	hydrogen peroxide metabolism	2	2	2	100.0	100.000	0.068
6749	glutathione metabolism	2	2	7	100.0	28.571	0.068
5992	trehalose biosynthesis	2	2	3	100.0	66.667	0.069
46351	disaccharide biosynthesis	2	2	3	100.0	66.667	0.069
6637	acyl-CoA metabolism	2	2	3	100.0	66.667	0.073
9066	aspartate family amino acid metabolism	2	2	7	100.0	28.571	0.074
6555	methionine metabolism	2	2	5	100.0	40.000	0.074
16973	poly(A)+ mRNA export from nucleus	2	2	2	100.0	100.000	0.078
19439	aromatic compound catabolism	6	7	15	85.7	46.667	0.000
6084	acetyl-CoA metabolism	15	18	30	83.3	60.000	0.000
51187	cofactor catabolism	15	18	30	83.3	60.000	0.000
8362	embryonic cuticle biosynthesis (sensu Insecta)	5	6	20	83.3	30.000	0.007
9109	coenzyme catabolism	14	17	29	82.4	58.621	0.000
6099	tricarboxylic acid cycle	13	16	27	81.3	59.259	0.000
9060	aerobic respiration	13	16	27	81.3	59.259	0.000
45333	cellular respiration	13	16	27	81.3	59.259	0.000
46356	acetyl-CoA catabolism	13	16	27	81.3	59.259	0.000
6090	pyruvate metabolism	4	5	10	80.0	50.000	0.017
6094	gluconeogenesis	4	5	10	80.0	50.000	0.017
19991	septate junction assembly	4	5	11	80.0	45.455	0.019
9410	response to xenobiotic stimulus	4	5	15	80.0	33.333	0.021
6805	xenobiotic metabolism	4	5	15	80.0	33.333	0.021
6570	tyrosine metabolism	7	9	13	77.8	69.231	0.005
51258	protein polymerization	13	17	33	76.5	51.515	0.000
7561	imaginal disc eversion	3	4	7	75.0	57.143	0.041
5991	trehalose metabolism	3	4	12	75.0	33.333	0.045
5984	disaccharide metabolism	3	4	12	75.0	33.333	0.045
6941	striated muscle contraction	3	4	37	75.0	10.811	0.049
9081	branched chain family amino acid metabolism	3	4	9	75.0	44.444	0.050
42594	response to starvation	3	4	12	75.0	33.333	0.051
44242	cellular lipid catabolism	3	4	9	75.0	44.444	0.054
46693	sperm storage	3	4	7	75.0	57.143	0.057
6586	indolalkylamine metabolism	3	4	14	75.0	28.571	0.065
6004	fucose metabolism	3	4	7	75.0	57.143	0.070
6778	porphyrin metabolism	8	11	17	72.7	64.706	0.001
6098	pentose-phosphate shunt	5	7	9	71.4	77.778	0.018

6739	NADP metabolism	5	7	9	71.4	77.778	0.018
6740	NADPH regeneration	5	7	9	71.4	77.778	0.018
6779	porphyrin biosynthesis	7	10	16	70.0	62.500	0.003
6120	mitochondrial electron transport\, NADH to ubiquinone	7	10	17	70.0	58.824	0.005
9072	aromatic amino acid family metabolism	10	15	28	66.7	53.571	0.001
42168	heme metabolism	6	9	12	66.7	75.000	0.013
42430	indole and derivative metabolism	4	6	17	66.7	35.294	0.042
42434	indole derivative metabolism	4	6	17	66.7	35.294	0.042
42438	melanin biosynthesis	4	6	10	66.7	60.000	0.044
9253	peptidoglycan catabolism	7	11	14	63.6	78.571	0.007
272	polysaccharide catabolism	10	16	25	62.5	64.000	0.001
44247	cellular polysaccharide catabolism	10	16	25	62.5	64.000	0.001
6046	N-acetylglucosamine catabolism	10	16	25	62.5	64.000	0.001
6043	glucosamine catabolism	10	16	25	62.5	64.000	0.001
46348	amino sugar catabolism	10	16	25	62.5	64.000	0.001
6032	chitin catabolism	10	16	25	62.5	64.000	0.001
6446	regulation of translational initiation	5	8	14	62.5	57.143	0.033
6582	melanin metabolism	5	8	14	62.5	57.143	0.033
6783	heme biosynthesis	5	8	11	62.5	72.727	0.033
43297	apical junction assembly	8	13	38	61.5	34.211	0.004
270	peptidoglycan metabolism	8	13	22	61.5	59.091	0.005
6092	main pathways of carbohydrate metabolism	37	63	131	58.7	48.092	0.000
42775	ATP synthesis coupled electron transport (sensu Eukaryota)	7	12	23	58.3	52.174	0.016
15980	energy derivation by oxidation of organic compounds	42	73	155	57.5	47.097	0.000
35151	regulation of tracheal tube size	8	14	27	57.1	51.852	0.005
7043	intercellular junction assembly	8	14	39	57.1	35.897	0.011
42773	ATP synthesis coupled electron transport	8	14	26	57.1	53.846	0.012
6961	antibacterial humoral response (sensu Protostomia)	8	14	43	57.1	32.558	0.014
16080	synaptic vesicle targeting	4	7	13	57.1	53.846	0.080
6406	mRNA export from nucleus	4	7	10	57.1	70.000	0.084
45176	apical protein localization	4	7	15	57.1	46.667	0.087
46364	monosaccharide biosynthesis	4	7	14	57.1	50.000	0.097
46165	alcohol biosynthesis	4	7	14	57.1	50.000	0.097
19319	hexose biosynthesis	4	7	14	57.1	50.000	0.097
51186	cofactor metabolism	58	103	192	56.3	53.646	0.000
6012	galactose metabolism	5	9	14	55.6	64.286	0.048
6769	nicotinamide metabolism	5	9	14	55.6	64.286	0.050

9166	nucleotide catabolism	5	9	15	55.6	60.000	0.058
7097	nuclear migration	5	9	23	55.6	39.130	0.063
16052	carbohydrate catabolism	37	67	119	55.2	56.303	0.000
44275	cellular carbohydrate catabolism	37	67	119	55.2	56.303	0.000
45087	innate immune response	11	20	41	55.0	48.780	0.005
51647	nucleus localization	6	11	26	54.5	42.308	0.045
45793	positive regulation of cell size	6	11	25	54.5	44.000	0.046
42060	wound healing	6	11	23	54.5	47.826	0.046
40003	cuticle biosynthesis (sensu Insecta)	6	11	43	54.5	25.581	0.047
7592	cuticle biosynthesis (sensu Protostomia and Nematoda)	6	11	43	54.5	25.581	0.047
6732	coenzyme metabolism	49	91	173	53.8	52.601	0.000
18958	phenol metabolism	7	13	24	53.8	54.167	0.023
6584	catecholamine metabolism	7	13	24	53.8	54.167	0.023
51168	nuclear export	7	13	20	53.8	65.000	0.026
45216	intercellular junction assembly and/or maintenance	9	17	46	52.9	36.957	0.011
6986	response to unfolded protein	9	17	50	52.9	34.000	0.016
6631	fatty acid metabolism	15	29	56	51.7	51.786	0.004
6952	defense response	39	76	342	51.3	22.222	0.000
6955	immune response	32	63	186	50.8	33.871	0.000
19318	hexose metabolism	35	70	150	50.0	46.667	0.000
6119	oxidative phosphorylation	28	56	100	50.0	56.000	0.000
6006	glucose metabolism	23	46	109	50.0	42.202	0.000
35150	regulation of tube size	8	16	33	50.0	48.485	0.028
9611	response to wounding	8	16	35	50.0	45.714	0.044
9620	response to fungi	7	14	45	50.0	31.111	0.046
6733	oxidoreduction coenzyme metabolism	7	14	23	50.0	60.870	0.054
6633	fatty acid biosynthesis	7	14	31	50.0	45.161	0.057
9063	amino acid catabolism	7	14	27	50.0	51.852	0.061
44274	organismal biosynthesis	7	14	46	50.0	30.435	0.071
42335	cuticle biosynthesis	7	14	46	50.0	30.435	0.071
7291	sperm individualization	6	12	23	50.0	52.174	0.087
6100	tricarboxylic acid cycle intermediate metabolism	6	12	30	50.0	40.000	0.092
5996	monosaccharide metabolism	36	73	155	49.3	47.097	0.000
46034	ATP metabolism	22	45	78	48.9	57.692	0.002
46164	alcohol catabolism	21	43	88	48.8	48.864	0.000
9607	response to biotic stimulus	39	80	355	48.8	22.535	0.000
19320	hexose catabolism	19	39	78	48.7	50.000	0.001

46365	monosaccharide catabolism	19	39	78	48.7	50.000	0.001
6007	glucose catabolism	19	39	78	48.7	50.000	0.001
42742	defense response to bacteria	17	35	112	48.6	31.250	0.005
9617	response to bacteria	17	35	112	48.6	31.250	0.005
19731	antibacterial humoral response	11	23	56	47.8	41.071	0.025
6754	ATP biosynthesis	21	44	76	47.7	57.895	0.002
6753	nucleoside phosphate metabolism	21	44	76	47.7	57.895	0.002
15985	energy coupled proton transport\, down electrochemical gradient	20	42	74	47.6	56.757	0.003
15986	ATP synthesis coupled proton transport	20	42	74	47.6	56.757	0.003
16065	humoral defense mechanism (sensu Protostomia)	10	21	70	47.6	30.000	0.029
6960	antimicrobial humoral response (sensu Protostomia)	10	21	70	47.6	30.000	0.029
35152	regulation of tracheal tube architecture	9	19	52	47.4	36.538	0.033
9141	nucleoside triphosphate metabolism	24	51	90	47.1	56.667	0.003
9116	nucleoside metabolism	8	17	38	47.1	44.737	0.046
44270	nitrogen compound catabolism	8	17	33	47.1	51.515	0.054
9310	amine catabolism	8	17	33	47.1	51.515	0.054
6818	hydrogen transport	23	49	97	46.9	50.515	0.003
15992	proton transport	23	49	97	46.9	50.515	0.003
9205	purine ribonucleoside triphosphate metabolism	23	49	85	46.9	57.647	0.004
9144	purine nucleoside triphosphate metabolism	23	49	85	46.9	57.647	0.004
9199	ribonucleoside triphosphate metabolism	23	49	85	46.9	57.647	0.004
6752	group transfer coenzyme metabolism	22	47	91	46.8	51.648	0.003
46483	heterocycle metabolism	21	45	91	46.7	49.451	0.001
46148	pigment biosynthesis	14	30	59	46.7	50.847	0.012
6334	nucleosome assembly	7	15	45	46.7	33.333	0.083
44262	cellular carbohydrate metabolism	110	237	463	46.4	51.188	0.000
6414	translational elongation	6	13	17	46.2	76.471	0.098
51188	cofactor biosynthesis	34	74	146	45.9	50.685	0.000
9201	ribonucleoside triphosphate biosynthesis	22	48	83	45.8	57.831	0.005
9206	purine ribonucleoside triphosphate biosynthesis	22	48	83	45.8	57.831	0.005
9145	purine nucleoside triphosphate biosynthesis	22	48	83	45.8	57.831	0.005
6575	amino acid derivative metabolism	11	24	52	45.8	46.154	0.031
6096	glycolysis	15	33	70	45.5	47.143	0.012
6576	biogenic amine metabolism	10	22	49	45.5	44.898	0.048
8652	amino acid biosynthesis	10	22	58	45.5	37.931	0.055
6066	alcohol metabolism	50	111	237	45.0	46.835	0.000

5975	carbohydrate metabolism	135	300	606	45.0	49.505	0.000
9142	nucleoside triphosphate biosynthesis	22	49	86	44.9	56.977	0.007
42440	pigment metabolism	16	36	73	44.4	49.315	0.012
6082	organic acid metabolism	74	167	350	44.3	47.714	0.000
19752	carboxylic acid metabolism	74	167	350	44.3	47.714	0.000
9613	response to pest\, pathogen or parasite	19	44	130	43.2	33.846	0.007
6030	chitin metabolism	39	91	165	42.9	55.152	0.000
9259	ribonucleotide metabolism	27	63	114	42.9	55.263	0.004
6959	humoral immune response	15	35	102	42.9	34.314	0.025
6041	glucosamine metabolism	41	96	172	42.7	55.814	0.000
6044	N-acetylglucosamine metabolism	41	96	172	42.7	55.814	0.000
6040	amino sugar metabolism	41	96	172	42.7	55.814	0.000
6091	generation of precursor metabolites and energy	169	396	772	42.7	51.295	0.000
19730	antimicrobial humoral response	14	33	97	42.4	34.021	0.045
9108	coenzyme biosynthesis	27	64	130	42.2	49.231	0.005
6163	purine nucleotide metabolism	27	64	117	42.2	54.701	0.006
9150	purine ribonucleotide metabolism	26	62	111	41.9	55.856	0.007
9260	ribonucleotide biosynthesis	26	62	112	41.9	55.357	0.009
44271	nitrogen compound biosynthesis	13	31	74	41.9	41.892	0.069
9309	amine biosynthesis	13	31	74	41.9	41.892	0.069
44248	cellular catabolism	84	201	383	41.8	52.480	0.000
43207	response to external biotic stimulus	22	53	172	41.5	30.814	0.007
6725	aromatic compound metabolism	29	70	137	41.4	51.095	0.002
6413	translational initiation	12	29	49	41.4	59.184	0.071
6164	purine nucleotide biosynthesis	26	63	115	41.3	54.783	0.009
9152	purine ribonucleotide biosynthesis	25	61	109	41.0	55.963	0.010
9308	amine metabolism	97	237	458	40.9	51.747	0.000
6461	protein complex assembly	18	44	112	40.9	39.286	0.022
5976	polysaccharide metabolism	42	103	187	40.8	55.080	0.000
44264	cellular polysaccharide metabolism	42	103	187	40.8	55.080	0.000
6807	nitrogen compound metabolism	99	243	475	40.7	51.158	0.000
6519	amino acid and derivative metabolism	55	135	277	40.7	48.736	0.000
7028	cytoplasm organization and biogenesis	17	42	85	40.5	49.412	0.030
19748	secondary metabolism	17	42	83	40.5	50.602	0.034
48515	spermatid differentiation	12	30	75	40.0	40.000	0.095
7286	spermatid development	12	30	75	40.0	40.000	0.095
6520	amino acid metabolism	47	118	245	39.8	48.163	0.002

6445	regulation of translation	15	38	95	39.5	40.000	0.069
6118	electron transport	112	287	552	39.0	51.993	0.000
9056	catabolism	98	253	607	38.7	41.680	0.000
9889	regulation of biosynthesis	17	44	106	38.6	41.509	0.064
31326	regulation of cellular biosynthesis	17	44	106	38.6	41.509	0.064
48232	male gamete generation	23	64	295	35.9	21.695	0.090
7283	spermatogenesis	23	64	295	35.9	21.695	0.090
44255	cellular lipid metabolism	37	104	214	35.6	48.598	0.027
9605	response to external stimulus	41	116	316	35.3	36.709	0.026
9057	macromolecule catabolism	53	151	283	35.1	53.357	0.012
43037	translation	38	109	217	34.9	50.230	0.025
9165	nucleotide biosynthesis	34	99	201	34.3	49.254	0.070
44265	cellular macromolecule catabolism	46	134	248	34.3	54.032	0.034
9058	biosynthesis	190	595	1195	31.9	49.791	0.000
44249	cellular biosynthesis	167	534	1058	31.3	50.473	0.003
51641	cellular localization	68	219	484	31.1	45.248	0.074
6810	transport	373	1238	2667	30.1	46.419	0.000
51234	establishment of localization	413	1384	3202	29.8	43.223	0.000
51179	localization	425	1436	3341	29.6	42.981	0.002
6508	proteolysis	148	506	999	29.2	50.651	0.097
8152	metabolism	1044	3599	7699	29.0	46.746	0.000
43170	macromolecule metabolism	596	2087	4513	28.6	46.244	0.001
44237	cellular metabolism	919	3225	6954	28.5	46.376	0.000
7582	physiological process	1377	4904	10712	28.1	45.780	0.000
50875	cellular physiological process	1226	4394	9679	27.9	45.397	0.000
44260	cellular macromolecule metabolism	435	1567	3386	27.8	46.279	0.086
