# Supplementary material for: Mechanisms Underlying Hypoxia Tolerance in Drosophila melanogaster: hairy as a Metabolic Switch
Source: PLoS Genet. 2008 Oct 17;4(10):e1000221. doi: 10.1371/journal.pgen.1000221 (PMC2556400; doi:10.1371/journal.pgen.1000221)
Supplement: Table S4 — List of Significantly Altered Biological Processes in Hypoxia-Selected Drosophila melanogaster at Adult Stage. (0.11 MB PDF) [file pgen.1000221.s005.pdf]

Table S4. List of Significantly Altered Biological Processes in Hypoxia-Selected *Drosophila melanogaster* at Adult Stage

GOID	GO Name	Number Changed	Number Measured	Number in GO	% Changed	% Present	p value
46683	response to organophosphorus	1	1	1	100.00	100.00	0.011
18130	heterocycle biosynthetic process	1	1	1	100.00	100.00	0.017
19428	allantoin biosynthetic process	1	1	1	100.00	100.00	0.017
255	allantoin metabolic process	1	1	1	100.00	100.00	0.017
6964	positive regulation of biosynthetic process of antibacterial peptides active against Gram-negative bacteria	1	1	1	100.00	100.00	0.019
2813	regulation of biosynthetic process of antibacterial peptides active against Gram-negative bacteria	1	1	1	100.00	100.00	0.019
2812	biosynthetic process of antibacterial peptides active against Gram-negative bacteria	1	1	1	100.00	100.00	0.019
30104	water homeostasis	1	1	1	100.00	100.00	0.019
7000	nucleolus organization and biogenesis	1	1	1	100.00	100.00	0.020
35160	maintenance of epithelial integrity\, open tracheal system	1	1	2	100.00	50.00	0.020
51295	establishment of meiotic spindle localization	1	1	1	100.00	100.00	0.020
35080	heat shock-mediated polytene chromosome puffing	1	1	1	100.00	100.00	0.020
35079	polytene chromosome puffing	1	1	1	100.00	100.00	0.020
46958	nonassociative learning	1	1	1	100.00	100.00	0.022
7029	endoplasmic reticulum organization and biogenesis	1	1	1	100.00	100.00	0.023
9229	thiamin diphosphate biosynthetic process	1	1	1	100.00	100.00	0.025
42724	thiamin and derivative biosynthetic process	1	1	1	100.00	100.00	0.025
6772	thiamin metabolic process	1	1	1	100.00	100.00	0.025
42357	thiamin diphosphate metabolic process	1	1	1	100.00	100.00	0.025
42723	thiamin and derivative metabolic process	1	1	1	100.00	100.00	0.025
6961	antibacterial humoral response	9	13	19	69.23	68.42	0.000
6805	xenobiotic metabolic process	3	5	5	60.00	100.00	0.000
9410	response to xenobiotic stimulus	3	5	5	60.00	100.00	0.000
19835	cytolysis	4	7	9	57.14	77.78	0.000
19732	antifungal humoral response	5	9	9	55.56	100.00	0.000
50829	defense response to Gram-negative bacterium	5	10	14	50.00	71.43	0.000

6966	antifungal humoral response	4	8	8	50.00	100.00	0.000
9405	pathogenesis	2	4	4	50.00	100.00	0.004
44403	symbiosis\, encompassing mutualism through parasitism	2	4	4	50.00	100.00	0.004
44419	interspecies interaction between organisms	2	4	4	50.00	100.00	0.004
46680	response to DDT	1	2	2	50.00	100.00	0.026
18987	osmoregulation	1	2	2	50.00	100.00	0.030
8627	induction of apoptosis by ionic changes	1	2	2	50.00	100.00	0.033
48132	female germ-line stem cell division	1	2	2	50.00	100.00	0.034
46131	pyrimidine ribonucleoside metabolic process	1	2	2	50.00	100.00	0.036
46087	cytidine metabolic process	1	2	2	50.00	100.00	0.036
9303	rRNA transcription	1	2	2	50.00	100.00	0.037
8306	associative learning	1	2	2	50.00	100.00	0.037
42742	defense response to bacterium	17	36	45	47.22	80.00	0.000
9617	response to bacterium	17	37	47	45.95	78.72	0.000
50830	defense response to Gram-positive bacterium	5	11	16	45.45	68.75	0.000
6967	positive regulation of antifungal peptide biosynthetic process	3	7	7	42.86	100.00	0.000
2810	regulation of antifungal peptide biosynthetic process	3	7	7	42.86	100.00	0.000
2783	antifungal peptide biosynthetic process	3	7	7	42.86	100.00	0.000
2781	antifungal peptide production	3	7	7	42.86	100.00	0.000
50832	defense response to fungus	5	12	12	41.67	100.00	0.000
19730	antimicrobial humoral response	13	32	41	40.63	78.05	0.000
16998	cell wall catabolic process	4	10	12	40.00	83.33	0.000
9620	response to fungus	5	13	13	38.46	100.00	0.000
6959	humoral immune response	13	35	45	37.14	77.78	0.000
19731	antibacterial humoral response	5	14	18	35.71	77.78	0.000
51707	response to other organism	17	48	58	35.42	82.76	0.000
6963	positive regulation of antibacterial peptide biosynthetic process	4	12	13	33.33	92.31	0.000
2808	regulation of antibacterial peptide biosynthetic process	4	12	13	33.33	92.31	0.000
2780	antibacterial peptide biosynthetic process	4	12	13	33.33	92.31	0.000
2778	antibacterial peptide production	4	12	13	33.33	92.31	0.000
17085	response to insecticide	1	3	3	33.33	100.00	0.044

42423	catecholamine biosynthetic process	1	3	3	33.33	100.00	0.053
8624	induction of apoptosis by extracellular signals	1	3	3	33.33	100.00	0.056
6596	polyamine biosynthetic process	1	3	3	33.33	100.00	0.059
6685	sphingomyelin catabolic process	1	3	3	33.33	100.00	0.061
9395	phospholipid catabolic process	1	3	3	33.33	100.00	0.061
51227	mitotic spindle assembly	1	3	3	33.33	100.00	0.067
45087	innate immune response	12	40	54	30.00	74.07	0.000
2807	positive regulation of antimicrobial peptide biosynthetic process	5	17	18	29.41	94.44	0.000
43043	peptide biosynthetic process	5	17	18	29.41	94.44	0.000
2805	regulation of antimicrobial peptide biosynthetic process	5	17	18	29.41	94.44	0.000
2440	production of molecular mediator of immune response	5	17	18	29.41	94.44	0.000
2775	antimicrobial peptide production	5	17	18	29.41	94.44	0.000
2777	antimicrobial peptide biosynthetic process	5	17	18	29.41	94.44	0.000
2252	immune effector process	5	18	22	27.78	81.82	0.000
6952	defense response	19	69	87	27.54	79.31	0.000
6960	antimicrobial humoral response	1	4	4	25.00	100.00	0.058
6835	dicarboxylic acid transport	1	4	4	25.00	100.00	0.063
30723	ovarian fusome organization and biogenesis	1	4	5	25.00	80.00	0.069
6213	pyrimidine nucleoside metabolic process	1	4	4	25.00	100.00	0.071
7352	zygotic determination of dorsal/ventral axis	1	4	4	25.00	100.00	0.071
6826	iron ion transport	1	4	4	25.00	100.00	0.074
6684	sphingomyelin metabolic process	1	4	4	25.00	100.00	0.078
42045	epithelial fluid transport	1	4	4	25.00	100.00	0.079
22401	adaptation of signaling pathway	1	4	4	25.00	100.00	0.079
16062	adaptation of rhodopsin mediated signaling	1	4	4	25.00	100.00	0.079
42044	fluid transport	1	4	4	25.00	100.00	0.079
46466	membrane lipid catabolic process	1	4	4	25.00	100.00	0.079
30149	sphingolipid catabolic process	1	4	4	25.00	100.00	0.079
6595	polyamine metabolic process	1	4	4	25.00	100.00	0.081
6144	purine base metabolic process	1	4	5	25.00	80.00	0.087
9951	polarity specification of dorsal/ventral axis	1	4	5	25.00	80.00	0.093

65001	specification of axis polarity	1	4	5	25.00	80.00	0.093
9949	polarity specification of anterior/posterior axis	1	4	5	25.00	80.00	0.093
9607	response to biotic stimulus	18	80	96	22.50	83.33	0.000
6955	immune response	14	66	83	21.21	79.52	0.000
6518	peptide metabolic process	5	24	25	20.83	96.00	0.000
30037	actin filament reorganization during cell cycle	1	5	5	20.00	100.00	0.093
42401	biogenic amine biosynthetic process	2	11	11	18.18	100.00	0.020
7311	maternal determination of dorsal/ventral axis\, oocyte\, germ-line encoded	1	6	6	16.67	100.00	0.098
42398	amino acid derivative biosynthetic process	2	13	13	15.38	100.00	0.024
2376	immune system process	15	104	123	14.42	84.55	0.000
6997	nuclear organization and biogenesis	2	16	16	12.50	100.00	0.040
6917	induction of apoptosis	2	16	16	12.50	100.00	0.042
8063	Toll signaling pathway	3	25	28	12.00	89.29	0.004
6633	fatty acid biosynthetic process	2	17	19	11.76	89.47	0.044
46394	carboxylic acid biosynthetic process	2	18	20	11.11	90.00	0.047
16053	organic acid biosynthetic process	2	18	20	11.11	90.00	0.047
7317	regulation of pole plasm oskar mRNA localization	2	18	18	11.11	100.00	0.049
9116	nucleoside metabolic process	2	19	20	10.53	95.00	0.045
43065	positive regulation of apoptosis	2	19	19	10.53	100.00	0.054
51704	multi-organism process	4	42	47	9.52	89.36	0.009
48878	chemical homeostasis	2	21	21	9.52	100.00	0.054
30706	oocyte differentiation (sensu Insecta)	2	22	23	9.09	95.65	0.076
12502	induction of programmed cell death	2	22	22	9.09	100.00	0.076
6030	chitin metabolic process	8	90	109	8.89	82.57	0.000
6044	N-acetylglucosamine metabolic process	8	95	114	8.42	83.33	0.002
6040	amino sugar metabolic process	8	95	114	8.42	83.33	0.002
6041	glucosamine metabolic process	8	95	114	8.42	83.33	0.002
6631	fatty acid metabolic process	3	36	38	8.33	94.74	0.025
6576	biogenic amine metabolic process	2	24	26	8.33	92.31	0.065
5976	polysaccharide metabolic process	8	102	122	7.84	83.61	0.002

44264	cellular polysaccharide metabolic process	8	102	122	7.84	83.61	0.002
6575	amino acid derivative metabolic process	2	27	29	7.41	93.10	0.083
8298	intracellular mRNA localization	3	41	42	7.32	97.62	0.043
7293	egg chamber formation (sensu Insecta)	3	42	46	7.14	91.30	0.046
32787	monocarboxylic acid metabolic process	3	45	48	6.67	93.75	0.047
9950	dorsal/ventral axis specification	3	46	48	6.52	95.83	0.064
9994	oocyte differentiation	5	79	83	6.33	95.18	0.020
6403	RNA localization	3	55	59	5.45	93.22	0.088
50896	response to stimulus	26	518	584	5.02	88.70	0.000
8219	cell death	7	143	155	4.90	92.26	0.019
16265	death	7	144	156	4.86	92.31	0.021
5975	carbohydrate metabolic process	15	317	349	4.73	90.83	0.002
9308	amine metabolic process	11	237	270	4.64	87.78	0.008
6807	nitrogen compound metabolic process	11	243	279	4.53	87.10	0.008
44249	cellular biosynthetic process	14	317	342	4.42	92.69	0.006
6629	lipid metabolic process	8	189	206	4.23	91.75	0.030
44262	cellular carbohydrate metabolic process	9	213	240	4.23	88.75	0.031
9056	catabolic process	11	267	282	4.12	94.68	0.014
42221	response to chemical stimulus	6	147	161	4.08	91.30	0.058
9798	axis specification	5	126	133	3.97	94.74	0.090
