# Supplementary material for: Mechanisms Underlying Hypoxia Tolerance in Drosophila melanogaster: hairy as a Metabolic Switch
Source: PLoS Genet. 2008 Oct 17;4(10):e1000221. doi: 10.1371/journal.pgen.1000221 (PMC2556400; doi:10.1371/journal.pgen.1000221)
Supplement: Table S5 — Primers for qRT-PCR and ChIP-PCR Assays. (0.02 MB PDF) [file pgen.1000221.s006.pdf]

Table S5. Primers for qRT-PCR and ChIP-PCR Assays

Primers for qRT-PCR:

Gene ID	Symbol	Primer Sequences
FBgn0039112	CG10219	Forward: 5′-GCTGCCTTCATAGCTCCATC-3′<br>Reverse: 5′-GGACGTTTCCAACGACAGAT-3′
FBgn0035611	CG13285	Forward: 5′-TTATCAGGGTCAGCGGAATC-3′<br>Reverse: 5′-AGCCACGATAGGAACTCGAA-3′
FBgn0040559	CG14359	Forward: 5′-CATTGCGCTCCTTGTAGGAT-3′<br>Reverse: 5′-TTGGACACGGTCCAGTTGTA-3′
FBgn0030341	CG1967	Forward: 5′-CCCCGAAATGTTGTCAATCT-3′<br>Reverse: 5′-TTACCGGGAGGGGTCTTAAC-3′
FBgn0030148	CG3106	Forward: 5′-ATCTTGTGCTTGGGTGGAAC-3′<br>Reverse: 5′-CCAAGTGGGAATTCGAGTGT-3′
FBgn0033005	CG3107	Forward: 5′-TTGATTCCCAGCATGTTGAA-3′<br>Reverse: 5′-TCGCCATCATGATTCCATAA-3′
FBgn0039453	CG6403	Forward: 5′-AAGGTGATAGCCGACGAATG-3′<br>Reverse: 5′-ACCTTGGTGTCGAGGTTCAC-3′
FBgn0038922	CG6439	Forward: 5′-ATCAATCCCGTGCTTAGTGC-3′<br>Reverse: 5′-AAAACGCCCTTAATGCACAC-3′
FBgn0037873	CG6666	Forward: 5′-GTCCCACGACATTAGCCACT-3′<br>Reverse: 5′-AATACCATTGGCGGTGTGAT-3′
FBgn0037955	CG6950	Forward: 5′-AAGCTCTGAGCCAGACAAGC-3′<br>Reverse: 5′-AAAGTCCTCCCCCAGATGTT-3′
FBgn0037230	CG9780	Forward: 5′-AACTGGCCCAAATGTACTCG-3′<br>Reverse: 5′-GTCCAATCGCTCATCTCCAT-3′
FBgn0001105	Gbeta13F	Forward: 5′-TCACATTTTTCCCGAATGGT-3′<br>Reverse: 5′-GTTGTCGTGCGAATACATGG-3′
FBgn0001248	Idh	Forward: 5′-AAGCGCGTAGAGGAGTTCAA-3′<br>Reverse: 5′-AAGACGGTTCCTCCCAAGAT-3′
FBgn0029869	l(1)G0030	Forward: 5′-AATGTGGGAGCCTATGTTCG-3′<br>Reverse: 5′-GCTTGCGGAAGTTCTTTACG-3′
FBgn0031024	l(1)G0156	Forward: 5′-CAAGGAGGGCAAATACCTCA-3′<br>Reverse: 5′-TGCTACAGCTTATGCGGATG-3′
FBgn0029889	l(1)G0255	Forward: 5′-CTGATCGTGTCCAATGTGCT-3′<br>Reverse: 5′-TGGATGCCATTCACACAGTT-3′
FBgn0033327	PGRP-SC1b	Forward: 5′-GGGCAACTACCTCAGCTACG-3′<br>Reverse: 5′-GGTAGTTCTGGACGCTCTGC-3′
FBgn0014028	SdhB	Forward: 5′-TCGACATCAACACCTCCAAG-3′<br>Reverse: 5′-TGTTGCGGTACTGCTCGTAG-3′

Primers for ChIP-PCR:

Genomic Location of Amplicon	Adjacent Gene	Primer Sequences
2R: 6722799..6723495	CG12344	Forward: 5′-CGGATGAAAGCCATTTGTTT-3′<br>Reverse: 5′-AAACACATTTCCAGCGAACC-3′
3R: 6941829..6942330	CG6629	Forward: 5′-GCGCCTTTTTGTTAGCTTTG-3′<br>Reverse: 5′-ATTTTCCTTTTGCCCAGCTT-3′
X: 6244048..6244656	l(1)G0030	Forward: 5′-ACGGCTGTCACAACCACATA-3′<br>Reverse: 5′-AGGAAAGCGATTGCGATAAA-3′
2R: 2695083..2695595	SdhB	Forward: 5′-TGCGCGCATGGTATATTTTA-3′<br>Reverse: 5′-TCTGCAAGTTCGGAGAATCA-3′
