# Supplementary material for: Mechanisms Underlying Hypoxia Tolerance in Drosophila melanogaster: hairy as a Metabolic Switch
Source: PLoS Genet. 2008 Oct 17;4(10):e1000221. doi: 10.1371/journal.pgen.1000221 (PMC2556400; doi:10.1371/journal.pgen.1000221)
Supplement: Table S6 — Summary of Genomic Locations of hairy Transcription Repressor Binding Elements in the cis-Regulatory Regions of Genes Encoding TCA Cycle Enzymes. (0.03 MB PDF) [file pgen.1000221.s007.pdf]

Table S6. Summary of Genomic Locations of hairy Transcription Repressor Binding Elements in the *cis*-Regulatory Regions of Genes Encoding TCA Cycle Enzymes

Gene ID	Fold Change	Size of *cis*-regulatory region*	hairy Binding Element	
			**Location / Orientation**	**Sequence**
l(1)G0030	-2.86	4154 bp	70-80 / -<br>1026-1036 / -<br>1088-1098 / +<br>1513-1523 / +	catCACGCtcc<br>aagCACGCacc<br>gggCACGCttg<br>tggCACGCggg
Idh	-1.89	5496 bp	44-54 / +<br>953-963 / +<br>1152-1162 / +<br>1807-1817 / +<br>1983-1993 / +<br>3642-3652 / +	cagCACGCtcc<br>gggCACGCact<br>cggCACGCgtt<br>acgCACGCtgc<br>ccaCACGCgcc<br>tgcCACGCgct
CG6439	-2.08	2500 bp	602-612 / -<br>865-875 / -<br>1096-1106 / -<br>2278-2288 / -	agcCACGCtac<br>gtgCACGCcag<br>cgcCACGCgct<br>ggcCACGCgaa
l(1)G0156	-1.96	2500 bp	517-527 / -<br>1022-1032 / -<br>1046-1056 / -<br>2438-2448 / -	aggCACGCcgg<br>ataCACGCgca<br>ataCACGCgtc<br>catCACGCgct
Nc73EF	-2.24	2500 bp	2161-2175 / -<br>2259-2273 / -	aagCACGCgaa<br>gaaCACGCgg
CG33791	-1.76	4260 bp	2620-2634 / -	agcCACGCaac
CG7430	-3.03	2500 bp	920-930 / +<br>931-941 / -<br>2287-2297 / +	gatCACGCtcc<br>tagCACGCtgc<br>attCACGCtcc
Scsalpha	-2.38	2500 bp	ND***	
CG11963	-1.89	2500 bp	494-504 / -	cacCACGCccc
SdhB	-2.78	2500 bp	1259-1269 / -<br>2176-2186 / -<br>2255-2265 / -<br>2354-2364 / +	cagCACGCcgc<br>tccCACGCggc<br>cacCACGCccc<br>aagCACGCgaa
CG10219	-1.59	2500 bp	550-560 / +<br>1307-1317 / -<br>1439-1449 / -	ttgCACGCtac<br>cggCACGCtga<br>cgtCACGCtcc
CG6666	-2.33	2500 bp	84-94 / +<br>996-1006 / +<br>1565-1575 / +	tctCACGCgac<br>gggCACGCtgt<br>tgcCACGCgct
l(1)G0255	-2.63	3652 bp	1048-1058 / -<br>3167-3177 / +<br>3374-3384 / -	cgcCACGCtac<br>gagCACGCctc<br>ctcCACGCgaa

CG6140	-1.75	2500 bp	816-826 / +	ataCACGCgat
CG4095	-2.70	2500 bp	85-95 / -<br>999-1009 / -	ctcCACGCgaa<br>tggCACGCagc
CG5214	-2.63	2500 bp	1148-1158 / +	gaaCACGCgca
Sucb	NS**	2500 bp	ND	
CG10748	NS	2500 bp	ND	
CG10749	NS	2500 bp	ND	
CG14740	NS	2500 bp	ND	
CG4706	NS	2500 bp	ND	
CG5718	NS	2500 bp	514-528 / +<br>589-603 / -<br>1192-1206 / +	gatCACGCgcc<br>tgaCACGCggc<br>aaaCACGCgag
CG5599	NS	2500 bp	ND	
CG6629	1.76	2500 bp	ND	

Note: * *cis*-regulatory region includes 2000bp upstream of the first transcription start site and 500bp downstream of the last transcription start site. ** NS: Not Significant; ***ND: Not Detected
