# Supplementary material for: Mechanisms Underlying Hypoxia Tolerance in Drosophila melanogaster: hairy as a Metabolic Switch
Source: PLoS Genet. 2008 Oct 17;4(10):e1000221. doi: 10.1371/journal.pgen.1000221 (PMC2556400; doi:10.1371/journal.pgen.1000221)
Supplement: Table S7 — Comparison of the TPI Genomic Locus between NF and AF with Multiple Sequence Alignment. (0.06 MB PDF) [file pgen.1000221.s008.pdf]

Table S7. Comparison of the TPI Genomic Locus between NF and AF with Multiple Sequence Alignment

---

Note:

ATGC: non-coding region
ATGC: 5′-untranslated region (5′-UTR)
ATGC: coding region/Open Reading Frame (ORF)
ATGC: 3′-untranslated region (3′-UTR)
ATGC: **Polymorphism**
#: *p*<0.0001 (CHI-Test)

```
NF-8            ATTTTGGCAAGCCTGTTGATAAAAGCTTTTCCAGAGCTTTTTGGAAGCGACGCCAAAACT 60
NF-6            ATTTTGGCAAGCCTGTTGATAAAAGCTTTTCCAGAGCTTTTTGGAAGCGACGCCAAAACT 60
NF-9            ATTTTGGCAAGCCTGTTGATAAAAGCTTTTCCAGAGCTTTTTGGAAGCGACGCCAAAACT 60
NF-7            ATTTTGGCAAGCCTGTTGATAAAAGCTTTTCCAGAGCTTTTTGGAAGCGACGCCAAAACT 60
NF-1            ATTTTGGCAAGCCTGTTGATAAAAGCTTTTCCAGAGCTTTTTGGAAGCGACGCCAAAACT 60
NF-5            ATTTTGGCAAGCCTGTTGATAAAAGCTTTTCCAGAGCTTTTTGGAAGCGACGCCAAAACT 60
NF-4            ATTTTGGCAAGCCTGTTGATAAAAGCTTTTCCAGAGCTTTTTGGAAGCGACGCCAAAACT 60
NF-2            ATTTTGGCAAGCCTGTTGATAAAAGCTTTTCCAGAGCTTTTTGGAAGCGACGCCAAAACT 60
AF-1            ATTTTGGCAAGCCTGTTGATAAAAGCTTTTCCAGAGCTTTTTGGAAGCGACGCCAAAACT 60
AF-3            ATTTTGGCAAGCCTGTTGATAAAAGCTTTTCCAGAGCTTTTTGGAAGCGACGCCAAAACT 60
AF-9            ATTTTGGCAAGCCTGTTGATAAAAGCTTTTCCAGAGCTTTTTGGAAGCGACGCCAAAACT 60
AF-8            ATTTTGGCAAGCCTGTTGATAAAAGCTTTTCCAGAGCTTTTTGGAAGCGACGCCAAAACT 60
AF-6            ATTTTGGCAAGCCTGTTGATAAAAGCTTTTCCAGAGCTTTTTGGAAGCGACGCCAAAACT 60
AF-2            ATTTTGGCAAGCCTGTTGATAAAAGCTTTTCCAGAGCTTTTTGGAAGCGACGCCAAAACT 60
AF-10           ATTTTGGCAAGCCTGTTGATAAAAGCTTTTCCAGAGCTTTTTGGAAGCGACGCCAAAACT 60
AF-7            ATTTTGGCAAGCCTGTTGATAAAAGCTTTTCCAGAGCTTTTTGGAAGCGACGCCAAAACT 60
AF-5            ATTTTGGCAAGCCTGTTGATAAAAGCTTTTCCAGAGCTTTTTGGAAGCGACGCCAAAACT 60
AF-4            ATTTTGGCAAGCCTGTTGATAAAAGCTTTTCCAGAGCTTTTTGGAAGCGACGCCAAAACT 60
NF-10           ATTTTGGCAAGCCTGTTGATAAAAGCTTTTCCAGAGCTTTTTGGAAGCGACGCCAAAACT 60
NF-3            ATTTTGGCAAGCCTGTTGATAAAAGCTTTTCCAGAGCTTTTTGGAAGCGACGCCAAAACT 60
                ************************************************************


                        #  #########            # #
NF-8            GTTGGAGCTCACTTGCTCAAAGTTCCCCATCAAAAGGTTGGAGTCGCTGCTAAATCTAAA 120
NF-6            GTTGGAGCTCACTTGCTCAAAGTTCCCCATCAAAAGGTTGGAGTCGCTGCTAAATCTAAA 120
NF-9            GTTGGAGCTCACTTGCTCAAAGTTCCCCATCAAAAGGTTGGAGTCGCTGCTAAATCTAAA 120
NF-7            GTTGGAGCTCACTTGCTCAAAGTTCCCCATCAAAAGGTTGGAGTCGCTGCTAAATCTAAA 120
NF-1            GTTGGAGCTCACTTGCTCAAAGTTCCCCATCAAAAGGTTGGAGTCGCTGCTAAATCTAAA 120
NF-5            GTTGGAGCTCACTTGCTCAAAGTTCCCCATCAAAAGGTTGGAGTCGCTGCTAAATCTAAA 120
NF-4            GTTGGAGCTCACTTGCTCAAAGTTCCCCATCAAAAGGTTGGAGTCGCTGCTAAATCTAAA 120
NF-2            GTTGGAGCTCACTTGCTCAAAGTTCCCCATCAAAAGGTTGGAGTCGCTGCTAAATCTAAA 120
AF-1            GTTGGAGCTCACTTGCTCAAAGTTCCCCATCAGATGGTTGGAGTCACTGCTAAATCTAAA 120
AF-3            GTTGGAGCCCA---------AGTTCCCCATCAGATGGTTGGAGTCACTGCTAAATCTAAA 111
AF-9            GTTGGAGCCCA---------AGTTCCCCATCAGATGGTTGGAGTCACTGCTAAATCTAAA 111
AF-8            GTTGGAGCCCA---------AGTTCCCCATCAGATGGTTGGAGTCACTGCTAAATCTAAA 111
AF-6            GTTGGAGCCCA---------AGTTCCCCATCAGATGGTTGGAGTCACTGCTAAATCTAAA 111
AF-2            GTTGGAGCCCA---------AGTTCCCCATTAGATGGTTGGAGTCGCTGCTAAATCTAAA 111
AF-10           GTTGGAGCCCA---------AGTTCCCCATTAGATGGTTGGAGTCGCTGCTAAATCTAAA 111
AF-7            GTTGGAGCCCA---------AGTTCCCCATTAGATGGTTGGAGTCGCTGCTAAATCTAAA 111
AF-5            GTTGGAGCCCA---------AGTTCCCCATTAGATGGTTGGAGTCGCTGCTAAATCTAAA 111
AF-4            GTTGGAGCCCA---------AGTTCCCCATTAGATGGTTGGAGTCGCTGCTAAATCTAAA 111
NF-10           GTTGGAGCCCA---------AGTTCCCCATTAGATGGTTGGAGTCGCTGCTAAATCTAAA 111
NF-3            GTTGGAGCCCA---------AGTTCCCCATTAGATGGTTGGAGTCGCTGCTAAATCTAAA 111
                ******** **         ********** * * ********** **************
```

```
NF-8      ACCGGCAACATTTTTAAAACCCCAACAAGCCGTTTATATAATTTCAGAATAACTTTTTTA 180
NF-6      ACCGGCAACATTTTTAAAACCCCAACAAGCCGTTTATATAATTTCAGAATAACTTTTTTA 180
NF-9      ACCGGCAACATTTTTAAAACCCCAACAAGCCGTTTATATAATTTCAGAATAACTTTTTTA 180
NF-7      ACCGGCAACATTTTTAAAACCCCAACAAGCCGTTTATATAATTTCAGAATAACTTTTTTA 180
NF-1      ACCGGCAACATTTTTAAAACCCCAACAAGCCGTTTATATAATTTCAGAATAACTTTTTTA 180
NF-5      ACCGGCAACATTTTTAAAACCCCAACAAGCCGTTTATATAATTTCAGAATAACTTTTTTA 180
NF-4      ACCGGCAACATTTTTAAAACCCCAACAAGCCGTTTATATAATTTCAGAATAACTTTTTTA 180
NF-2      ACCGGCAACATTTTTAAAACCCCAACAAGCCGTTTATATAATTTCAGAATAACTTTTTTA 180
AF-1      ACCGGCAACATTTTTAAAATCCCAACAAGCCGTTTATATAATTTCAGAATAACTTTTTTA 180
AF-3      ACCGGCAACATTTTTAAAATCCCAACAAGCCGTTTATATAATTTCAGAATAACTTTTTTA 171
AF-9      ACCGGCAACATTTTTAAAATCCCAACAAGCCGTTTATATAATTTCAGAATAACTTTTTTA 171
AF-8      ACCGGCAACATTTTTAAAATCCCAACAAGCCGTTTATATAATTTCAGAATAACTTTTTTA 171
AF-6      ACCGGCAACATTTTTAAAACCCCAACAAGCCGTTTATATAATTTCAGAATAACTTTTTTA 171
AF-2      ACCGGCAACATTTTTAAAACCCCAACAAGCCGTTTATATAATTTCAGAATAACTTTTTTA 171
AF-10     ACCGGCAACATTTTTAAAACCCCAACAAGCCGTTTATATAATTTCAGAATAACTTTTTTA 171
AF-7      ACCGGCAACATTTTTAAAACCCCAACAAGCCGTTTATATAATTTCAGAATAACTTTTTTA 171
AF-5      ACCGGCAACATTTTTAAAACCCCAACAAGCCGTTTATATAATTTCAGAATAACTTTTTTA 171
AF-4      ACCGGCAACATTTTTAAAACCCCAACAAGCCGTTTATATAATTTCAGAATAACTTTTTTA 171
NF-10     ACCGGCAACATTTTTAAAACCCCAACAAGCCGTTTATATAATTTCAGAATAACTTTTTTA 171
NF-3      ACCGGCAACATTTTTAAAACCCCAACAAGCCGTTTATATAATTTCAGAATAACTTTTTTA 171
          ******************* ****************************************

NF-8      CTACTTCTGACGAACACAAATTGGTTACCACAATAGAAATGCCGCCTGGAGTACTCAATG 240
NF-6      CTACTTCTGACGAACACAAATTGGTTACCACAATAGAAATGCCGCCTGGAGTACTCAATG 240
NF-9      CTACTTCTGACGAACACAAATTGGTTACCACAATAGAAATGCCGCCTGGAGTACTCAATG 240
NF-7      CTACTTCTGACGAACACAAATTGGTTACCACAATAGAAATGCCGCCTGGAGTACTCAATG 240
NF-1      CTACTTCTGACGAACACAAATTGGTTACCACAATAGAAATGCCGCCTGGAGTACTCAATG 240
NF-5      CTACTTCTGACGAACACAAATTGGTTACCACAATAGAAATGCCGCCTGGAGTACTCAATG 240
NF-4      CTACTTCTGACGAACACAAATTGGTTACCACAATAGAAATGCCGCCTGGAGTACTCAATG 240
NF-2      CTACTTCTGACGAACACAAATTGGTTACCACAATAGAAATGCCGCCTGGAGTACTCAATG 240
AF-1      CTACTTCTGACGAACACAAATTGGTTACCACAATAGAAATGCCGCCTGGAGTACTCAATA 240
AF-3      CTACTTCTGACTAACACAAATTGGTTACCACAATAGAAATGCCGCCTGGAGTACTCAATG 231
AF-9      CTACTTCTGACTAACACAAATTGGTTACCACAATAGAAATGCCGCCTGGAGTACTCAATG 231
AF-8      CTACTTCTGACTAACACAAATTGGTTACCACAATAGAAATGCCGCCTGGAGTACTCAATG 231
AF-6      CTACTTCTGACTAACACAAATTGGTTACCACAATAGAAATGCCGCCTGGAGTACTCAATG 231
AF-2      CTACTTCTGACGAACACAAATTGGTTACCACAATAGAAATGCCGCCTGGAGTACTCAATG 231
AF-10     CTACTTCTGACGAACACAAATTGGTTACCACAATAGAAATGCCGCCTGGAGTACTCAATG 231
AF-7      CTACTTCTGACGAACACAAATTGGTTACCACAATAGAAATGCCGCCTGGAGTACTCAATG 231
AF-5      CTACTTCTGACGAACACAAATTGGTTACCACAATAGAAATGCCGCCTGGAGTACTCAATG 231
AF-4      CTACTTCTGACGAACACAAATTGGTTACCACAATAGAAATGCCGCCTGGAGTACTCAATG 231
NF-10     CTACTTCTGACGAACACAAATTGGTTACCACAATAGAAATGCCGCCTGGAGTACTCAATG 231
NF-3      CTACTTCTGACGAACACAAATTGGTTACCACAATAGAAATGCCGCCTGGAGTACTCAATG 231
          *********** ***********************************************

NF-8      ATCTGAACAGGAACGGTATCCAGTGCATTGCATCAGTAACGCTTCTCCAGCGATTCCCAT 300
NF-6      ATCTGAACAGGAACGGTATCCAGTGCATTGCATCAGTAACGCTTCTCCAGCGATTCCCAT 300
NF-9      ATCTGAACAGGAACGGTATCCAGTGCATTGCATCAGTAACGCTTCTCCAGCGATTCCCAT 300
NF-7      ATCTGAACAGGAACGGTATCCAGTGCATTGCATCAGTAACGCTTCTCCAGCGATTCCCAT 300
NF-1      ATCTGAACAGGAACGGTATCCAGTGCATTGCATCAGTAACGCTTCTCCAGCGATTCCCAT 300
NF-5      ATCTGAACAGGAACGGTATCCAGTGCATTGCATCAGTAACGCTTCTCCAGCGATTCCCAT 300
NF-4      ATCTGAACAGGAACGGTATCCAGTGCATTGCATCAGTAACGCTTCTCCAGCGATTCCCAT 300
NF-2      ATCTGAACAGGAACGGTATCCAGTGCATTGCATCAGTAACGCTTCTCCAGCGATTCCCAT 300
AF-1      ATCTGAACAGGAACGGTATCCAGTGCATTGCATCAGTAACGCTTCTCCAGCGATTCCCGT 300
AF-3      ATCTGAACAGGAACAGTATCCAGTGCATTGCATCAGTAACGCTTCTCCAGCGATTCCCAT 291
AF-9      ATCTGAACAGGAACAGTATCCAGTGCATTGCATCAGTAACGCTTCTCCAGCGATTCCCAT 291
AF-8      ATCTGAACAGGAACAGTATCCAGTGCATTGCATCAGTAACGCTTCTCCAGCGATTCCCAT 291
AF-6      ATCTGAACAGGAACGGTATCCAGTGCATTGCATCAGTAACGCTTCTCCAGCGATTCCCAT 291
AF-2      ATCTGAACAGGAACGGTATCCAGTGCATTGCATCAGTAACGCTTCTCCAGCGATTCCCGT 291
```

```
AF-10          ATCTGAACAGGAACGGTATCCAGTGCATTGCATCAGTAACGCTTCTCCAGCGATTCCCGT 291
AF-7           ATCTGAACAGGAACGGTATCCAGTGCATTGCATCAGTAACGCTTCTCCAGCGATTCCCGT 291
AF-5           ATCTGAACAGGAACGGTATCCAGTGCATTGCATCAGTAACGCTTCTCCAGCGATTCCCGT 291
AF-4           ATCTGAACAGGAACGGTATCCAGTGCATTGCATCAGTAACGCTTCTCCAGCGATTCCCGT 291
NF-10          ATCTGAACAGGAACGGTATCCAGTGCATTGCATCAGTAACGCTTCTCCAGCGATTCCCGT 291
NF-3           ATCTGAACAGGAACGGTATCCAGTGCATTGCATCAGTAACGCTTCTCCAGCGATTCCCGT 291
               ************** ******************************************* *

NF-8           ATCATCGGAACCACCACACCATCCCCACCATGGACCAGAACCGACCCACTGTGGGTGGTG 360
NF-6           ATCATCGGAACCACCACACCATCCCCACCATGGACCAGAACCGACCCACTGTGGGTGGTG 360
NF-9           ATCATCGGAACCACCACACCATCCCCACCATGGACCAGAACCGACCCACTGTGGGTGGTG 360
NF-7           ATCATCGGAACCACCACACCATCCCCACCATGGACCAGAACCGACCCACTGTGGGTGGTG 360
NF-1           ATCATCGGAACCACCACACCATCCCCACCATGGACCAGAACCGACCCACTGTGGGTGGTG 360
NF-5           ATCATCGGAACCACCACACCATCCCCACCATGGACCAGAACCGACCCACTGTGGGTGGTG 360
NF-4           ATCATCGGAACCACCACACCATCCCCACCATGGACCAGAACCGACCCACTGTGGGTGGTG 360
NF-2           ATCATCGGAACCACCACACCATCCCCACCATGGACCAGAACCGACCCACTGTGGGTGGTG 360
AF-1           ATCATCGGAACCACCACACCATCCCCACCATGGACCAGAACCGACCCACTGTGGGTGGTG 360
AF-3           ATCATCGGAACCACCACACCATCCCCACCATGGACCAGAACCGACCCACTGTGGGTGGTG 351
AF-9           ATCATCGGAACCACCACACCATCCCCACCATGGACCAGAACCGACCCACTGTGGGTGGTG 351
AF-8           ATCATCGGAACCACCACACCATCCCCACCATGGACCAGAACCGACCCACTGTGGGTGGTG 351
AF-6           ATCATCGGAACCACCACACCATCCCCACCATGGACCAGAACCGACCCACTGTGGGTGGTG 351
AF-2           ATCATCGGAACCACCACACCATCCCCACCATGGACCAGAACCGACCCACTGTGGGTGGTG 351
AF-10          ATCATCGGAACCACCACACCATCCCCACCATGGACCAGAACCGACCCACTGTGGGTGGTG 351
AF-7           ATCATCGGAACCACCACACCATCCCCACCATGGACCAGAACCGACCCACTGTGGGTGGTG 351
AF-5           ATCATCGGAACCACCACACCATCCCCACCATGGACCAGAACCGACCCACTGTGGGTGGTG 351
AF-4           ATCATCGGAACCACCACACCATCCCCACCATGGACCAGAACCGACCCACTGTGGGTGGTG 351
NF-10          ATCATCGGAACCACCACACCATCCCCACCATGGACCAGAACCGACCCACTGTGGGTGGTG 351
NF-3           ATCATCGGAACCACCACACCATCCCCACCATGGACCAGAACCGACCCACTGTGGGTGGTG 351
               ************************************************************

NF-8           TTACGGCGGAGGTACTAAAAGCCGTCGTTTCGGAAGCTCTGCTGGGCAAATTCACAGCTG 420
NF-6           TTACGGCGGAGGTACTAAAAGCCGTCGTTTCGGAAGCTCTGCTGGGCAAATTCACAGCTG 420
NF-9           TTACGGCGGAGGTACTAAAAGCCGTCGTTTCGGAAGCTCTGCTGGGCAAATTCACAGCTG 420
NF-7           TTACGGCGGAGGTACTAAAAGCCGTCGTTTCGGAAGCTCTGCTGGGCAAATTCACAGCTG 420
NF-1           TTACGGCGGAGGTACTAAAAGCCGTCGTTTCGGAAGCTCTGCTGGGCAAATTCACAGCTG 420
NF-5           TTACGGCGGAGGTACTAAAAGCCGTCGTTTCGGAAGCTCTGCTGGGCAAATTCACAGCTG 420
NF-4           TTACGGCGGAGGTACTAAAAGCCGTCGTTTCGGAAGCTCTGCTGGGCAAATTCACAGCTG 420
NF-2           TTACGGCGGAGGTACTAAAAGCCGTCGTTTCGGAAGCTCTGCTGGGCAAATTCACAGCTG 420
AF-1           TTACGGCGGAGGTACTAAAAGCCGTCGTTTCGGAAGCTCTGCTGGGCAAATTCACAGCTG 420
AF-3           TTACGGCGGAGGTACTAAAAGCCGTCGTTTCGGAAGCTCTGCTGGGCAAATTCACAGCTG 411
AF-9           TTACGGCGGAGGTACTAAAAGCCGTCGTTTCGGAAGCTCTGCTGGGCAAATTCACAGCTG 411
AF-8           TTACGGCGGAGGTACTAAAAGCCGTCGTTTCGGAAGCTCTGCTGGGCAAATTCACAGCTG 411
AF-6           TTACGGCGGAGGTACTAAAAGCCGTCGTTTCGGAAGCTCTGCTGGGCAAATTCACAGCTG 411
AF-2           TTACGGCGGAGGTACTAAAAGCCGTCGTTTCGGAAGCTCTGCTGGGCAAATTCACAGCTG 411
AF-10          TTACGGCGGAGGTACTAAAAGCCGTCGTTTCGGAAGCTCTGCTGGGCAAATTCACAGCTG 411
AF-7           TTACGGCGGAGGTACTAAAAGCCGTCGTTTCGGAAGCTCTGCTGGGCAAATTCACAGCTG 411
AF-5           TTACGGCGGAGGTACTAAAAGCCGTCGTTTCGGAAGCTCTGCTGGGCAAATTCACAGCTG 411
AF-4           TTACGGCGGAGGTACTAAAAGCCGTCGTTTCGGAAGCTCTGCTGGGCAAATTCACAGCTG 411
NF-10          TTACGGCGGAGGTACTAAAAGCCGTCGTTTCGGAAGCTCTGCTGGGCAAATTCACAGCTG 411
NF-3           TTACGGCGGAGGTACTAAAAGCCGTCGTTTCGGAAGCTCTGCTGGGCAAATTCACAGCTG 411
               ************************************************************

NF-8           TTTTCCCAAAGCTTTTCAAGTGCCAGTGGAAGACTTACGAAGGTCAAAAGAAGGTTAGTA 480
NF-6           TTTTCCCAAAGCTTTTCAAGTGCCAGTGGAAGACTTACGAAGGTCAAAAGAAGGTTAGTA 480
NF-9           TTTTCCCAAAGCTTTTCAAGTGCCAGTGGAAGACTTACGAAGGTCAAAAGAAGGTTAGTA 480
NF-7           TTTTCCCAAAGCTTTTCAAGTGCCAGTGGAAGACTTACGAAGGTCAAAAGAAGGTTAGTA 480
NF-1           TTTTCCCAAAGCTTTTCAAGTGCCAGTGGAAGACTTACGAAGGTCAAAAGAAGGTTAGTA 480
NF-5           TTTTCCCAAAGCTTTTCAAGTGCCAGTGGAAGACTTACGAAGGTCAAAAGAAGGTTAGTA 480
NF-4           TTTTCCCAAAGCTTTTCAAGTGCCAGTGGAAGACTTACGAAGGTCAAAAGAAGGTTAGTA 480
```

```
NF-2                TTTTCCCAAAGCTTTTCAAGTGCCAGTGGAAGACTTACGAAGGTCAAAAGAAGGTTAGTA 480
AF-1                TTTTCCCAAAGCTTTTCAAGTGCCAGTGGAAGACTTACGAAGGTCAAAAGAAGGTTAGTA 480
AF-3                TTTTCCCAAAGCTTTTCAAGTGCCAGTGGAAGACTTACGAAGGTCAAAAGAAGGTTAGTA 471
AF-9                TTTTCCCAAAGCTTTTCAAGTGCCAGTGGAAGACTTACGAAGGTCAAAAGAAGGTTAGTA 471
AF-8                TTTTCCCAAAGCTTTTCAAGTGCCAGTGGAAGACTTACGAAGGTCAAAAGAAGGTTAGTA 471
AF-6                TTTTCCCAAAGCTTTTCAAGTGCCAGTGGAAGACTTACGAAGGTCAAAAGAAGGTTAGTA 471
AF-2                TTTTCCCAAAGCTTTTCAAGTGCCAGTGGAAGACTTACGAAGGTCAAAAGAAGGTTAGTA 471
AF-10               TTTTCCCAAAGCTTTTCAAGTGCCAGTGGAAGACTTACGAAGGTCAAAAGAAGGTTAGTA 471
AF-7                TTTTCCCAAAGCTTTTCAAGTGCCAGTGGAAGACTTACGAAGGTCAAAAGAAGGTTAGTA 471
AF-5                TTTTCCCAAAGCTTTTCAAGTGCCAGTGGAAGACTTACGAAGGTCAAAAGAAGGTTAGTA 471
AF-4                TTTTCCCAAAGCTTTTCAAGTGCCAGTGGAAGACTTACGAAGGTCAAAAGAAGGTTAGTA 471
NF-10               TTTTCCCAAAGCTTTTCAAGTGCCAGTGGAAGACTTACGAAGGTCAAAAGAAGGTTAGTA 471
NF-3                TTTTCCCAAAGCTTTTCAAGTGCCAGTGGAAGACTTACGAAGGTCAAAAGAAGGTTAGTA 471
                    ************************************************************

NF-8                CATAAGCAGACGAACTTCGAGGTGGGTGTGTGCTCTGTGCCGGGATTAATCTCGAATCTG 540
NF-6                CATAAGCAGACGAACTTCGAGGTGGGTGTGTGCTCTGTGCCGGGATTAATCTCGAATCTG 540
NF-9                CATAAGCAGACGAACTTCGAGGTGGGTGTGTGCTCTGTGCCGGGATTAATCTCGAATCTG 540
NF-7                CATAAGCAGACGAACTTCGAGGTGGGTGTGTGCTCTGTGCCGGGATTAATCTCGAATCTG 540
NF-1                CATAAGCAGACGAACTTCGAGGTGGGTGTGTGCTCTGTGCCGGGATTAATCTCGAATCTG 540
NF-5                CATAAGCAGACGAACTTCGAGGTGGGTGTGTGCTCTGTGCCGGGATTAATCTCGAATCTG 540
NF-4                CATAAGCAGACGAACTTCGAGGTGGGTGTGTGCTCTGTGCCGGGATTAATCTCGAATCTG 540
NF-2                CATAAGCAGACGAACTTCGAGGTGGGTGTGTGCTCTGTGCCGGGATTAATCTCGAATCTG 540
AF-1                CATAAGCAGACGAACTTCGAGGTGGGTGTGTGCTCTGTGCCGGGATTAATCTCGAATCTG 540
AF-3                CATAAGCAGACGAACTTCGAGGTGGGTGTGTGCTCTGTGCCGGGATTAATCTCGAATCTG 531
AF-9                CATAAGCAGACGAACTTCGAGGTGGGTGTGTGCTCTGTGCCGGGATTAATCTCGAATCTG 531
AF-8                CATAAGCAGACGAACTTCGAGGTGGGTGTGTGCTCTGTGCCGGGATTAATCTCGAATCTG 531
AF-6                CATAAGCAGACGAACTTCGAGGTGGGTGTGTGCTCTGTGCCGGGATTAATCTCGAATCTG 531
AF-2                CATAAGCAGACGAACTTCGAGGTGGGTGTGTGCTCTGTGCCGGGATTAATCTCGAATCTG 531
AF-10               CATAAGCAGACGAACTTCGAGGTGGGTGTGTGCTCTGTGCCGGGATTAATCTCGAATCTG 531
AF-7                CATAAGCAGACGAACTTCGAGGTGGGTGTGTGCTCTGTGCCGGGATTAATCTCGAATCTG 531
AF-5                CATAAGCAGACGAACTTCGAGGTGGGTGTGTGCTCTGTGCCGGGATTAATCTCGAATCTG 531
AF-4                CATAAGCAGACGAACTTCGAGGTGGGTGTGTGCTCTGTGCCGGGATTAATCTCGAATCTG 531
NF-10               CATAAGCAGACGAACTTCGAGGTGGGTGTGTGCTCTGTGCCGGGATTAATCTCGAATCTG 531
NF-3                CATAAGCAGACGAACTTCGAGGTGGGTGTGTGCTCTGTGCCGGGATTAATCTCGAATCTG 531
                    ************************************************************

NF-8                GGAAAAATCTGAGTGGAAAAGTCGACGGCGAGCCTCCAGTCATCGAGTTACCCACTTGAA 600
NF-6                GGAAAAATCTGAGTGGAAAAGTCGACGGCGAGCCTCCAGTCATCGAGTTACCCACTTGAA 600
NF-9                GGAAAAATCTGAGTGGAAAAGTCGACGGCGAGCCTCCAGTCATCGAGTTACCCACTTGAA 600
NF-7                GGAAAAATCTGAGTGGAAAAGTCGACGGCGAGCCTCCAGTCATCGAGTTACCCACTTGAA 600
NF-1                GGAAAAATCTGAGTGGAAAAGTCGACGGCGAGCCTCCAGTCATCGAGTTACCCACTTGAA 600
NF-5                GGAAAAATCTGAGTGGAAAAGTCGACGGCGAGCCTCCAGTCATCGAGTTACCCACTTGAA 600
NF-4                GGAAAAATCTGAGTGGAAAAGTCGACGGCGAGCCTCCAGTCATCGAGTTACCCACTTGAA 600
NF-2                GGAAAAATCTGAGTGGAAAAGTCGACGGCGAGCCTCCAGTCATCGAGTTACCCACTTGAA 600
AF-1                GGAAAAATCTGAGTGGAAAAGTCGACGGCGAGCCTCCAGTCATCGAGTTACCCACTCGAA 600
AF-3                GGAAAAATCTGAGTGGAAAAGTCGACGGCGAGCCTCCAGTCATCGAGTTACCCACTTGAA 591
AF-9                GGAAAAATCTGAGTGGAAAAGTCGACGGCGAGCCTCCAGTCATCGAGTTACCCACTTGAA 591
AF-8                GGAAAAATCTGAGTGGAAAAGTCGACGGCGAGCCTCCAGTCATCGAGTTACCCACTTGAA 591
AF-6                GGAAAAATCTGAGTGGAAAAGTCGACGGCGAGCCTCCAGTCATCGAGTTACCCACTTGAA 591
AF-2                GGAAAAATCTGAGTGGAAAAGTCGACGGCGAGCCTCCAGTCATCGAGTTACCCACTTGAA 591
AF-10               GGAAAAATCTGAGTGGAAAAGTCGACGGCGAGCCTCCAGTCATCGAGTTACCCACTTGAA 591
AF-7                GGAAAAATCTGAGTGGAAAAGTCGACGGCGAGCCTCCAGTCATCGAGTTACCCACTTGAA 591
AF-5                GGAAAAATCTGAGTGGAAAAGTCGACGGCGAGCCTCCAGTCATCGAGTTACCCACTTGAA 591
AF-4                GGAAAAATCTGAGTGGAAAAGTCGACGGCGAGCCTCCAGTCATCGAGTTACCCACTTGAA 591
NF-10               GGAAAAATCTGAGTGGAAAAGTCGACGGCGAGCCTCCAGTCATCGAGTTACCCACTTGAA 591
NF-3                GGAAAAATCTGAGTGGAAAAGTCGACGGCGAGCCTCCAGTCATCGAGTTACCCACTTGAA 591
                    ******************************************************** ***
```

```
NF-8            ATTATCAGTTCCAAACACTCTAATAGCAGTCCCCTTGTTTTGTCCCCCGATCCGCAGTTC 660
NF-6            ATTATCAGTTCCAAACACTCTAATAGCAGTCCCCTTGTTTTGTCCCCCGATCCGCAGTTC 660
NF-9            ATTATCAGTTCCAAACACTCTAATAGCAGTCCCCTTGTTTTGTCCCCCGATCCGCAGTTC 660
NF-7            ATTATCAGTTCCAAACACTCTAATAGCAGTCCCCTTGTTTTGTCCCCCGATCCGCAGTTC 660
NF-1            ATTATCAGTTCCAAACACTCTAATAGCAGTCCCCTTGTTTTGTCCCCCGATCCGCAGTTC 660
NF-5            ATTATCAGTTCCAAACACTCTAATAGCAGTCCCCTTGTTTTGTCCCCCGATCCGCAGTTC 660
NF-4            ATTATCAGTTCCAAACACTCTAATAGCAGTCCCCTTGTTTTGTCCCCCGATCCGCAGTTC 660
NF-2            ATTATCAGTTCCAAACACTCTAATAGCAGTCCCCTTGTTTTGTCCCCCGATCCGCAGTTC 660
AF-1            ATTATCAGTTCCAAACACTCTAATAGCAGTCCCCTTGTTTTGTCCCCCGATCCGCAGTTC 660
AF-3            ATTATCAGTTCCAAACACTCTAATAGTAGTCCCCTTGTTTTGTCCCCCGATCCGCAGTTC 651
AF-9            ATTATCAGTTCCAAACACTCTAATAGTAGTCCCCTTGTTTTGTCCCCCGATCCGCAGTTC 651
AF-8            ATTATCAGTTCCAAACACTCTAATAGTAGTCCCCTTGTTTTGTCCCCCGATCCGCAGTTC 651
AF-6            ATTATCAGTTCCAAACACTCTAATAGTAGTCCCCTTGTTTTGTCCCCCGATCCGCAGTTC 651
AF-2            ATTATCAGTTCCAAACACTCTAATAGCAGTCCCCTTGTTTTGTCCCCCGATCCGCAGTTC 651
AF-10           ATTATCAGTTCCAAACACTCTAATAGCAGTCCCCTTGTTTTGTCCCCCGATCCGCAGTTC 651
AF-7            ATTATCAGTTCCAAACACTCTAATAGCAGTCCCCTTGTTTTGTCCCCCGATCCGCAGTTC 651
AF-5            ATTATCAGTTCCAAACACTCTAATAGCAGTCCCCTTGTTTTGTCCCCCGATCCGCAGTTC 651
AF-4            ATTATCAGTTCCAAACACTCTAATAGCAGTCCCCTTGTTTTGTCCCCCGATCCGCAGTTC 651
NF-10           ATTATCAGTTCCAAACACTCTAATAGCAGTCCCCTTGTTTTGTCCCCCGATCCGCAGTTC 651
NF-3            ATTATCAGTTCCAAACACTCTAATAGCAGTCCCCTTGTTTTGTCCCCCGATCCGCAGTTC 651
                ************************** *********************************

NF-8            TACGCCAATTTCAGCACCGATTGCACCGACAGCAACAGCAACAACATGAGCCGAAAGTTC 720
NF-6            TACGCCAATTTCAGCACCGATTGCACCGACAGCAACAGCAACAACATGAGCCGAAAGTTC 720
NF-9            TACGCCAATTTCAGCACCGATTGCACCGACAGCAACAGCAACAACATGAGCCGAAAGTTC 720
NF-7            TACGCCAATTTCAGCACCGATTGCACCGACAGCAACAGCAACAACATGAGCCGAAAGTTC 720
NF-1            TACGCCAATTTCAGCACCGATTGCACCGACAGCAACAGCAACAACATGAGCCGAAAGTTC 720
NF-5            TACGCCAATTTCAGCACCGATTGCACCGACAGCAACAGCAACAACATGAGCCGAAAGTTC 720
NF-4            TACGCCAATTTCAGCACCGATTGCACCGACAGCAACAGCAACAACATGAGCCGAAAGTTC 720
NF-2            TACGCCAATTTCAGCACCGATTGCACCGACAGCAACAGCAACAACATGAGCCGAAAGTTC 720
AF-1            TACGCCAATTTCAGCACCGATTGCACCGACAGCAACAGCAACAACATGAGCCGAAAGTTC 720
AF-3            TACGCCAATTTCAGCACCGATTGCACCGACAGCAACAGCAACAACATGAGCCGAAAGTTC 711
AF-9            TACGCCAATTTCAGCACCGATTGCACCGACAGCAACAGCAACAACATGAGCCGAAAGTTC 711
AF-8            TACGCCAATTTCAGCACCGATTGCACCGACAGCAACAGCAACAACATGAGCCGAAAGTTC 711
AF-6            TACGCCAATTTCAGCACCGATTGCACCGACAGCAACAGCAACAACATGAGCCGAAAGTTC 711
AF-2            TACGCCAATTTCAGCACCGATTGCACCGACAGCAACAGCAACAACATGAGCCGAAAGTTC 711
AF-10           TACGCCAATTTCAGCACCGATTGCACCGACAGCAACAGCAACAACATGAGCCGAAAGTTC 711
AF-7            TACGCCAATTTCAGCACCGATTGCACCGACAGCAACAGCAACAACATGAGCCGAAAGTTC 711
AF-5            TACGCCAATTTCAGCACCGATTGCACCGACAGCAACAGCAACAACATGAGCCGAAAGTTC 711
AF-4            TACGCCAATTTCAGCACCGATTGCACCGACAGCAACAGCAACAACATGAGCCGAAAGTTC 711
NF-10           TACGCCAATTTCAGCACCGATTGCACCGACAGCAACAGCAACAACATGAGCCGAAAGTTC 711
NF-3            TACGCCAATTTCAGCACCGATTGCACCGACAGCAACAGCAACAACATGAGCCGAAAGTTC 711
                ************************************************************

NF-8            TGCGTGGGAGGCAACTGGAAGATGAACGGCGACCAGAAGTCCATCGCCGAGATCGCCAAG 780
NF-6            TGCGTGGGAGGCAACTGGAAGATGAACGGCGACCAGAAGTCCATCGCCGAGATCGCCAAG 780
NF-9            TGCGTGGGAGGCAACTGGAAGATGAACGGCGACCAGAAGTCCATCGCCGAGATCGCCAAG 780
NF-7            TGCGTGGGAGGCAACTGGAAGATGAACGGCGACCAGAAGTCCATCGCCGAGATCGCCAAG 780
NF-1            TGCGTGGGAGGCAACTGGAAGATGAACGGCGACCAGAAGTCCATCGCCGAGATCGCCAAG 780
NF-5            TGCGTGGGAGGCAACTGGAAGATGAACGGCGACCAGAAGTCCATCGCCGAGATCGCCAAG 780
NF-4            TGCGTGGGAGGCAACTGGAAGATGAACGGCGACCAGAAGTCCATCGCCGAGATCGCCAAG 780
NF-2            TGCGTGGGAGGCAACTGGAAGATGAACGGCGACCAGAAGTCCATCGCCGAGATCGCCAAG 780
AF-1            TGCGTGGGAGGCAACTGGAAGATGAACGGCGACCAGAAGTCCATCGCCGAGATCGCCAAG 780
AF-3            TGCGTGGGAGGCAACTGGAAGATGAACGGCGACCAGAAGTCCATCGCCGAGATCGCCAAG 771
AF-9            TGCGTGGGAGGCAACTGGAAGATGAACGGCGACCAGAAGTCCATCGCCGAGATCGCCAAG 771
AF-8            TGCGTGGGAGGCAACTGGAAGATGAACGGCGACCAGAAGTCCATCGCCGAGATCGCCAAG 771
AF-6            TGCGTGGGAGGCAACTGGAAGATGAACGGCGACCAGAAGTCCATCGCCGAGATCGCCAAG 771
AF-2            TGCGTGGGAGGCAACTGGAAGATGAACGGCGACCAGAAGTCCATCGCCGAGATCGCCAAG 771
AF-10           TGCGTGGGAGGCAACTGGAAGATGAACGGCGACCAGAAGTCCATCGCCGAGATCGCCAAG 771
```

```
AF-7           TGCGTGGGAGGCAACTGGAAGATGAACGGCGACCAGAAGTCCATCGCCGAGATCGCCAAG 771
AF-5           TGCGTGGGAGGCAACTGGAAGATGAACGGCGACCAGAAGTCCATCGCCGAGATCGCCAAG 771
AF-4           TGCGTGGGAGGCAACTGGAAGATGAACGGCGACCAGAAGTCCATCGCCGAGATCGCCAAG 771
NF-10          TGCGTGGGAGGCAACTGGAAGATGAACGGCGACCAGAAGTCCATCGCCGAGATCGCCAAG 771
NF-3           TGCGTGGGAGGCAACTGGAAGATGAACGGCGACCAGAAGTCCATCGCCGAGATCGCCAAG 771
               ************************************************************

NF-8           ACCCTGAGCTCGGCCGCCCTCGACCCCAACACGGAGGTGGTCATCGGCTGCCCGGCCATC 840
NF-6           ACCCTGAGCTCGGCCGCCCTCGACCCCAACACGGAGGTGGTCATCGGCTGCCCGGCCATC 840
NF-9           ACCCTGAGCTCGGCCGCCCTCGACCCCAACACGGAGGTGGTCATCGGCTGCCCGGCCATC 840
NF-7           ACCCTGAGCTCGGCCGCCCTCGACCCCAACACGGAGGTGGTCATCGGCTGCCCGGCCATC 840
NF-1           ACCCTGAGCTCGGCCGCCCTCGACCCCAACACGGAGGTGGTCATCGGCTGCCCGGCCATC 840
NF-5           ACCCTGAGCTCGGCCGCCCTCGACCCCAACACGGAGGTGGTCATCGGCTGCCCGGCCATC 840
NF-4           ACCCTGAGCTCGGCCGCCCTCGACCCCAACACGGAGGTGGTCATCGGCTGCCCGGCCATC 840
NF-2           ACCCTGAGCTCGGCCGCCCTCGACCCCAACACGGAGGTGGTCATCGGCTGCCCGGCCATC 840
AF-1           ACCCTGAGCTCGGCCGCCCTCGACCCCAACACGGAGGTGGTCATCGGCTGCCCGGCCATC 840
AF-3           ACCCTGAGCTCGGCCGCCCTCGACCCCAACACGGAGGTGGTCATCGGCTGCCCGGCCATC 831
AF-9           ACCCTGAGCTCGGCCGCCCTCGACCCCAACACGGAGGTGGTCATCGGCTGCCCGGCCATC 831
AF-8           ACCCTGAGCTCGGCCGCCCTCGACCCCAACACGGAGGTGGTCATCGGCTGCCCGGCCATC 831
AF-6           ACCCTGAGCTCGGCCGCCCTCGACCCCAACACGGAGGTGGTCATCGGCTGCCCGGCCATC 831
AF-2           ACCCTGAGCTCGGCCGCCCTCGACCCCAACACGGAGGTGGTCATCGGCTGCCCGGCCATC 831
AF-10          ACCCTGAGCTCGGCCGCCCTCGACCCCAACACGGAGGTGGTCATCGGCTGCCCGGCCATC 831
AF-7           ACCCTGAGCTCGGCCGCCCTCGACCCCAACACGGAGGTGGTCATCGGCTGCCCGGCCATC 831
AF-5           ACCCTGAGCTCGGCCGCCCTCGACCCCAACACGGAGGTGGTCATCGGCTGCCCGGCCATC 831
AF-4           ACCCTGAGCTCGGCCGCCCTCGACCCCAACACGGAGGTGGTCATCGGCTGCCCGGCCATC 831
NF-10          ACCCTGAGCTCGGCCGCCCTCGACCCCAACACGGAGGTGGTCATCGGCTGCCCGGCCATC 831
NF-3           ACCCTGAGCTCGGCCGCCCTCGACCCCAACACGGAGGTGGTCATCGGCTGCCCGGCCATC 831
               ************************************************************

NF-8           TACCTGATGTACGCCCGCAACCTGCTGCCCTGCGAGCTGGGTCTGGCCGGCCAGAATGCC 900
NF-6           TACCTGATGTACGCCCGCAACCTGCTGCCCTGCGAGCTGGGTCTGGCCGACCAGAATGCC 900
NF-9           TACCTGATGTACGCCCGCAACCTGCTGCCCTGCGAGCTGGGTCTGGCCGGCCAGAATGCC 900
NF-7           TACCTGATGTACGCCCGCAACCTGCTGCCCTGCGAGCTGGGTCTGGCCGGCCAGAATGCC 900
NF-1           TACCTGATGTACGCCCGCAACCTGCTGCCCTGCGAGCTGGGTCTGGCCGGCCAGAATGCC 900
NF-5           TACCTGATGTACGCCCGCAACCTGCTGCCCTGCGAGCTGGGTCTGGCCGGCCAGAATGCC 900
NF-4           TACCTGATGTACGCCCGCAACCTGCTGCCCTGCGAGCTGGGTCTGGCCGGCCAGAATGCC 900
NF-2           TACCTGATGTACGCCCGCAACCTGCTGCCCTGCGAGCTGGGTCTGGCCGGCCAGAATGCC 900
AF-1           TACCTGATGTACGCCCGCAACCTGCTGCCCTGCGAGCTGGGTCTGGCCGGCCAGAATGCC 900
AF-3           TACCTGATGTACGCCCGCAACCTGCTGCCCTGCGAGCTGGGTCTGGCCGGCCAGAATGCC 891
AF-9           TACCTGATGTACGCCCGCAACCTGCTGCCCTGCGAGCTGGGTCTGGCCGGCCAGAATGCC 891
AF-8           TACCTGATGTACGCCCGCAACCTGCTGCCCTGCGAGCTGGGTCTGGCCGGCCAGAATGCC 891
AF-6           TACCTGATGTACGCCCGCAACCTGCTGCCCTGCGAGCTGGGTCTGGCCGGCCAGAATGCC 891
AF-2           TACCTGATGTACGCCCGCAACCTGCTGCCCTGCGAGCTGGGTCTGGCCGGCCAGAATGCC 891
AF-10          TACCTGATGTACGCCCGCAACCTGCTGCCCTGCGAGCTGGGTCTGGCCGGCCAGAATGCC 891
AF-7           TACCTGATGTACGCCCGCAACCTGCTGCCCTGCGAGCTGGGTCTGGCCGGCCAGAATGCC 891
AF-5           TACCTGATGTACGCCCGCAACCTGCTGCCCTGCGAGCTGGGTCTGGCCGGCCAGAATGCC 891
AF-4           TACCTGATGTACGCCCGCAACCTGCTGCCCTGCGAGCTGGGTCTGGCCGGCCAGAATGCC 891
NF-10          TACCTGATGTACGCCCGCAACCTGCTGCCCTGCGAGCTGGGTCTGGCCGGCCAGAATGCC 891
NF-3           TACCTGATGTACGCCCGCAACCTGCTGCCCTGCGAGCTGGGTCTGGCCGGCCAGAATGCC 891
               ************************************************* **********

NF-8           TACAAGGTGGCCAAGGGCGCATTCACCGGCGAGATCTCGCCTGCGATGCTGAAGGACATC 960
NF-6           TACAAGGTGGCCAAGGGCGCATTCACCGGCGAGATCTCGCCTGCGATGCTGAAGGACATC 960
NF-9           TACAAGGTGGCCAAGGGCGCATTCACCGGCGAGATCTCGCCTGCGATGCTGAAGGACATC 960
NF-7           TACAAGGTGGCCAAGGGCGCATTCACCGGCGAGATCTCGCCTGCGATGCTGAAGGACATC 960
NF-1           TACAAGGTGGCCAAGGGCGCATTCACCGGCGAGATCTCGCCTGCGATGCTGAAGGACATC 960
NF-5           TACAAGGTGGCCAAGGGCGCATTCACCGGCGAGATCTCGCCTGCGATGCTGAAGGACATC 960
NF-4           TACAAGGTGGCCAAGGGCGCATTCACCGGCGAGATCTCGCCTGCGATGCTGAAGGACATC 960
NF-2           TACAAGGTGGCCAAGGGCGCATTCACCGGCGAGATCTCGCCTGCGATGCTGAAGGACATC 960
```

```
AF-1        TACAAGGTGGCCAAGGGCGCATTCACCGGCGAGATCTCGCCTGCGATGCTGAAGGACATC 960
AF-3        TACAAGGTGGCCAAGGGCGCATTCACCGGCGAGATCTCGCCTGCGATGCTGAAGGACATC 951
AF-9        TACAAGGTGGCCAAGGGCGCATTCACCGGCGAGATCTCGCCTGCGATGCTGAAGGACATC 951
AF-8        TACGAGGTGGCCAAGGGCGCATTCACCGGCGAGATCTCGCCTGCGATGCTGAAGGACATC 951
AF-6        TACAAGGTGGCCAAGGGCGCATTCACCGGCGAGATCTCGCCTGCGATGCTGAAGGACATC 951
AF-2        TACAAGGTGGCCAAGGGCGCATTCACCGGCGAGATCTCGCCTGCGATGCTGAAGGACATC 951
AF-10       TACAAGGTGGCCAAGGGCGCATTCACCGGCGAGATCTCGCCTGCGATGCTGAAGGACATC 951
AF-7        TACAAGGTGGCCAAGGGCGCATTCACCGGCGAGATCTCGCCTGCGATGCTGAAGGACATC 951
AF-5        TACAAGGTGGCCAAGGGCGCATTCACCGGCGAGATCTCGCCTGCGATGCTGAAGGACATC 951
AF-4        TACAAGGTGGCCAAGGGCGCATTCACCGGCGAGATCTCGCCTGCGATGCTGAAGGACATC 951
NF-10       TACAAGGTGGCCAAGGGCGCATTCACCGGCGAGATCTCGCCTGCGATGCTGAAGGACATC 951
NF-3        TACAAGGTGGCCAAGGGCGCATTCACCGGCGAGATCTCGCCTGCGATGCTGAAGGACATC 951
            *** ********************************************************

NF-8        GGCGCCGACTGGGTGATCCTGGGACACTCGGAGCGTCGCGCCATCTTCGGCGAATCGGAC 1020
NF-6        GGCGCCGACTGGGTGATCCTGGGACACTCGGAGCGTCGCGCCATCTTCGGCGAATCGGAC 1020
NF-9        GGCGCCGACTGGGTGATCCTGGGACACTCGGAGCGTCGCGCCATCTTCGGCGAATCGGAC 1020
NF-7        GGCGCCGACTGGGTGATCCTGGGACACTCGGAGCGTCGCGCCATCTTCGGCGAATCGGAC 1020
NF-1        GGCGCCGACTGGGTGATCCTGGGACACTCGGAGCGTCGCGCCATCTTCGGCGAATCGGAC 1020
NF-5        GGCGCCGACTGGGTGATCCTGGGACACTCGGAGCGTCGCGCCATCTTCGGCGAATCGGAC 1020
NF-4        GGCGCCGACTGGGTGATCCTGGGACACTCGGAGCGTCGCGCCATCTTCGGCGAATCGGAC 1020
NF-2        GGCGCCGACTGGGTGATCCTGGGACACTCGGAGCGTCGCGCCATCTTCGGCGAATCGGAC 1020
AF-1        GGCGCCGACTGGGTGATCCTGGGGCACTCGGAGCGTCGCGCCATCTTCGGCGAATCGGAC 1020
AF-3        GGCGCCGACTGGGTGATCCTGGGACACTCGGAGCGTCGCGCCATCTTCGGCGAATCGGAC 1011
AF-9        GGCGCCGACTGGGTGATCCTGGGACACTCGGAGCGTCGCGCCATCTTCGGCGAATCGGAC 1011
AF-8        GGCGCCGACTGGGTGATCCTGGGACACTCGGAGCGTCGCGCCATCTTCGGCGAATCGGAC 1011
AF-6        GGCGCCGACTGGGTGATCCTGGGACACTCGGAGCGTCGCGCCATCTTCGGCGAATCGGAC 1011
AF-2        GGCGCCGACTGGGTGATCCTGGGACACTCGGAGCGTCGCGCCATCTTCGGCGAATCGGAC 1011
AF-10       GGCGCCGACTGGGTGATCCTGGGACACTCGGAGCGTCGCGCCATCTTCGGCGAATCGGAC 1011
AF-7        GGCGCCGACTGGGTGATCCTGGGACACTCGGAGCGTCGCGCCATCTTCGGCGAATCGGAC 1011
AF-5        GGCGCCGACTGGGTGATCCTGGGACACTCGGAGCGTCGCGCCATCTTCGGCGAATCGGAC 1011
AF-4        GGCGCCGACTGGGTGATCCTGGGACACTCGGAGCGTCGCGCCATCTTCGGCGAATCGGAC 1011
NF-10       GGCGCCGACTGGGTGATCCTGGGACACTCGGAGCGTCGCGCCATCTTCGGCGAATCGGAC 1011
NF-3        GGCGCCGACTGGGTGATCCTGGGACACTCGGAGCGTCGCGCCATCTTCGGCGAATCGGAC 1011
            *********************** ************************************

NF-8        GCCCTGATCGCCGAGAAGGCCGAGCACGCCCTGGCCGAGGGCCTCAAGGTCATCGCGTGC 1080
NF-6        GCCCTGATCGCCGAGAAGGCCGAGCACGCCCTGGCCGAGGGCCTCAAGGTCATCGCCTGC 1080
NF-9        GCCCTGATCGCCGAGAAGGCCGAGCACGCCCTGGCCGAGGGCCTCAAGGTCATCGCCTGC 1080
NF-7        GCCCTGATCGCCGAGAAGGCCGAGCACGCCCTGGCCGAGGGCCTCAAGGTCATCGCCTGC 1080
NF-1        GCCCTGATCGCCGAGAAGGCCGAGCACGCCCTGGCCGAGGGCCTCAAGGTCATCGCCTGC 1080
NF-5        GCCCTGATCGCCGAGAAGGCCGAGCACGCCCTGGCCGAGGGCCTCAAGGTCATCGCCTGC 1080
NF-4        GCCCTGATCGCCGAGAAGGCCGAGCACGCCCTGGCCGAGGGCCTCAAGGTCATCGCCTGC 1080
NF-2        GCCCTGATCGCCGAGAAGGCCGAGCACGCCCTGGCCGAGGGCCTCAAGGTCATCGCCTGC 1080
AF-1        GCCCTGATCGCCGAGAAGGCCGAGCACGCCCTGGCCGAGGGCCTCAAGGTCATCGCCTGC 1080
AF-3        GCCCTGATCGCCGAGAAGGCCGAGCACGCCCTGGCCGAGGGCCTGAAGGTCATCGCCTGC 1071
AF-9        GCCCTGATCGCCGAGAAGGCCGAGCACGCCCTGGCCGAGGGCCTGAAGGTCATCGCCTGC 1071
AF-8        GCCCTGATCGCCGAGAAGGCCGAGCACGCCCTGGCCGAGGGCCTGAAGGTCATCGCCTGC 1071
AF-6        GCCCTGATCGCCGAGAAGGCCGAGCACGCCCTGGCCGAGGGCCTGAAGGTCATCGCCTGC 1071
AF-2        GCCCTGATCGCCGAGAAGGCCGAGCACGCCCTGGCCGAGGGCCTCAAGGTCATCGCCTGC 1071
AF-10       GCCCTGATCGCCGAGAAGGCCGAGCACGCCCTGGCCGAGGGCCTCAAGGTCATCGCCTGC 1071
AF-7        GCCCTGATCGCCGAGAAGGCCGAGCACGCCCTGACCGAGGGCCTCAAGGTCATCGCCTGC 1071
AF-5        GCCCTGATCGCCGAGAAGGCCGAGCACGCCCTGGCCGAGGGACTCAAGGTCATCGCCTGC 1071
AF-4        GCCCTGATCGCCGAGAAGGCCGAGCACGCCCTGGCCGAGGGCCTCAAGGTCATCGCCTGC 1071
NF-10       GCCCTGATCGCCGAGAAGGCCGAGCACGCCCTGGCCGAGGGCCTCAAGGTCATCGCCTGC 1071
NF-3        GCCCTGATCGCCGAGAAGGCCGAGCACGCCCTGGCCGAGGGCCTCAAGGTCATCGCCTGC 1071
            ********************************* ******* ** *********** ***

NF-8        ATTGGTGAGACCCTGGAGGAGCGCGAGGCCGGCAAGACCAACGAGGTGGTGGCCCGCCAA 1140
```

```
NF-6      ATTGGTGAGACCCTGGAGGAGCGCGAGGCCGGCAAGACCAACGAGGTGGTGGCCCGCCAA 1140
NF-9      ATTGGTGAGACCCTGGAGGAGCGCGAGGCCGGCAAGACCAACGAGGTGGTGGCCCGCCAA 1140
NF-7      ATTGGTGAGACCCTGGAGGAGCGCGAGGCCGGCAAGACCAACGAGGTGGTGGCCCGCCAA 1140
NF-1      ATTGGTGAGACCCTGGAGGAGCGCGAGGCCGGCAAGACCAACGAGGTGGTGGCCCGCCAA 1140
NF-5      ATTGGTGAGACCCTGGAGGAGCGCGAGGCCGGCAAGACCAACGAGGTGGTGGCCCGCCAA 1140
NF-4      ATTGGTGAGACCCTGGAGGAGCGCGAGGCCGGCAAGACCAACGAGGTGGTGGCCCGCCAA 1140
NF-2      ATTGGTGAGACCCTGGAGGAGCGCGAGGCCGGCAAGACCAACGAGGTGGTGGCCCGCCAA 1140
AF-1      ATTGGTGAGACCCTGGAGGAGCGTGAGGCCGGCAAGACCAACGAGGTGGTGGCCCGCCAA 1140
AF-3      ATTGGTGAGACCCTGGAGGAGCGCGAGGCCGGCAAGACCAACGAGGTGGTGGCCCGGCAA 1131
AF-9      ATTGGTGAGACCCTGGAGGAGCGCGAGGCCGGCAAGACCAACGAGGTGGTGGCCCGGCAA 1131
AF-8      ATTGGTGAGACCCTGGAGGAGCGCGAGGCCGGCAAGACCAACGAGGTGGTGGCCCGGCAA 1131
AF-6      ATTGGTGAGACCCTGGAGGAGCGCGAGGCCGGCAAGACCAACGAGGTGGTGGCCCGGCAA 1131
AF-2      ATTGGTGAGACCCTGGAGGAGCGTGAGGCCGGCAAGACCAACGAGGTGGTGGGCCGCCAA 1131
AF-10     ATTGGTGAGACCCTGGAGGAGCGTGAGGCCGGCAAGACCAACGAGGTGGTGGCCCGCCAA 1131
AF-7      ATTGGTGAGACCCTGGAGGAGCGTGAGGCCGGCAAGACCAACGAGGTGGTGGCCCGCCAA 1131
AF-5      ATTGGTGAGACCCTGGAGGAGCGTGAGGCCGGCAAGACCAACGAGGTGGTGGCCCGCCAA 1131
AF-4      ATTGGTGAGACCCTGGAGGAGCGTGAGGCCGGCAAGACCAACGAGGTGGTGGCCCGCCAA 1131
NF-10     ATTGGTGAGACCCTGGAGGAGCGTGAGGCCGGCAAGACCAACGAGGTGGTGGCCCGCCAA 1131
NF-3      ATTGGTGAGACCCTGGAGGAGCGTGAGGCCGGCAAGACCAACGAGGTGGTGGCCCGCCAA 1131
          *********************** **************************** *** ***

                                                                 #
NF-8      ATGTGCGCCTACGCCCAGAAGATCAAGGACTGGAAGAACGTGGTGGTGGCCTACGAACCC 1200
NF-6      ATGTGCGCCTACGCCCAGAAGATCAAGGACTGGAAGAACGTGGTGGTGGCCTACGAACCC 1200
NF-9      ATGTGCGCCTACGCCCAGAAGATCAAGGACTGGAAGAACGTGGTGGTGGCCTACGAACCC 1200
NF-7      ATGTGCGCCTACGCCCAGAAGATCAAGGACTGGAAGAACGTGGTGGTGGCCTACGAACCC 1200
NF-1      ATGTGCGCCTACGCCCAGAAGATCAAGGACTGGAAGAACGTGGTGGTGGCCTACGAACCC 1200
NF-5      ATGTGCGCCTACGCCCAGAAGATCAAGGACTGGAAGAACGTGGTGGTGGCCTACGAACCC 1200
NF-4      ATGTGCGCCTACGCCCAGAAGATCAAGGACTGGAAGAACGTGGTGGTGGCCTACGAACCC 1200
NF-2      ATGTGCGCCTACGCCCAGAAGATCAAGGACTGGAAGAACGTGGTGGTGGCCTACGAGCCC 1200
AF-1      ATGTGCGCCTACGCCCAGAAGATCAAGGACTGGAAGAACGTGGTGGTGGCCTACGAACCC 1200
AF-3      ATGTGCGCCTACGCCCAGAAGATCAAGGACTGGAAGAACGTGGTGGTGGCCTACGAGCCC 1191
AF-9      ATGTGCGCCTACGCCCAGAAGATCAAGGACTGGAAGAACGTGGTGGTGGCCTACGAGCCC 1191
AF-8      ATGTGCGCCTACGCCCAGAAGATCAAGGACTGGAAGAACGTGGTGGTGGCCTACGAGCCC 1191
AF-6      ATGTGCGCCTACGCCCAGAAGATCAAGGACTGGAAGAACGTGGTGGTGGCCTACGAGCCC 1191
AF-2      ATGTGCGCCTACGCCCAGAAGATCAAGGACTGGAAGAACGTGGTGGTGGCCTACGAGCCC 1191
AF-10     ATGTGCGCCTACGCCCAGAAGATCAAGGACTGGAAGAACGTGGTGGTGGCCTACGAGCCC 1191
AF-7      ATGTGCGCCTACGCCCAGAAGATCAAGGACTGGAAGAACGTGGTGGTGGCCTACGAGCCC 1191
AF-5      ATGTGCGCCTACGCCCAGAAGATCAAGGACTGGAAGAACGTGGTGGTGGCCTACGAGCCC 1191
AF-4      ATGTGCGCCTACGCCCAGAAGATCAAGGACTGGAAGAACGTGGTGGTGGCCTACGAGCCC 1191
NF-10     ATGTGCGCCTACGCCCAGAAGATCAAGGACTGGAAGAACGTGGTGGTGGCCTACGAGCCC 1191
NF-3      ATGTGCGCCTACGCCCAGAAGATCAAGGACTGGAAGAACGTGGTGGTGGCCTACGAGCCC 1191
          ******************************************************** ***

NF-8      GTCTGGGCCATTGGCACCGGCAAGACCGCCACACCCGATCAGGTAAGGTCGCCC-----C 1255
NF-6      GTCTGGGCCATTGGCACCGGCAAGACCGCCACACCCGATCAGGTAAGGTCGCCC-----C 1255
NF-9      GTCTGGGCCATTGGCACCGGCAAGACCGCCACACCCGATCAGGTAAGGTCGCCC-----C 1255
NF-7      GTCTGGGCCATTGGCACCGGCAAGACCGCCACACCCGATCAGGTAAGGTCGCCC-----C 1255
NF-1      GTCTGGGCCATTGGCACCGGCAAGACCGCCACACCCGATCAGGTAAGGTCGCCC-----C 1255
NF-5      GTCTGGGCCATTGGCACCGGCAAGACCGCCACACCCGATCAGGTAAGGTCGCCC-----C 1255
NF-4      GTCTGGGCCATTGGCACCGGCAAGACCGCCACACCCGATCAGGTAAGGTCGCCC-----C 1255
NF-2      GTCTGGGCCATTGGCACCGGCAAGACCGCCACACCCGATCAGGTAAGGTCGCCCTTAGCC 1260
AF-1      GTCTGGGCCATTGGCACCGGCAAGACCGCCACACCCGATCAGGTAAGGTCGCCC-----C 1255
AF-3      GTCTGGGCCATTGGCACCGGCAAGACCGCCACACCCGATCAGGTAAGGTCGCCC-----C 1246
AF-9      GTCTGGGCCATTGGCACCGGCAAGACCGCCACACCCGATCAGGTAAGGTCGCCC-----C 1246
AF-8      GTCTGGGCCATTGGCACCGGCAAGACCGCCACACCCGATCAGGTAAGGTCGCCC-----C 1246
AF-6      GTCTGGGCCATTGGCACCGGCAAGACCGCCACACCCGATCAGGTAAGGTCGCCC-----C 1246
AF-2      GTCTGGGCCATTGGCACCGGCAAGACCGCCACACCCGATCAGGTAAGGTCGCCCTTAGCC 1251
AF-10     GTCTGGGCCATTGGCACCGGCAAGACCGCCACACCCGATCAGGTAAGGTCGCCCTTAGCC 1251
```

```
AF-7            GTCTGGGCCATTGGCACCGGCAAGACCGCCACACCCGATCAGGTAAGGTCGCCCTTAGCC 1251
AF-5            GTCTGGGCCATTGGCACCGGCAAGACCGCCACACCCGATCAGGTAAGGTCGCCCTTAGCC 1251
AF-4            GTCTGGGCCATTGGCACCGGCAAGACCGCCACACCCGATCAGGTAAGGTCGCCCTTAGCC 1251
NF-10           GTCTGGGCCATTGGCACCGGCAAGACCGCCACACCCGATCAGGTAAGGTCGCCCTTAGCC 1251
NF-3            GTCTGGGCCATTGGCACCGGCAAGACCGCCACACCCGATCAGGTAAGGTCGCCCTTAGCC 1251
                ******************************************************     *

NF-8            CAGAGACTAATCCCTTCCAGAACTGATATTTTCCCTTCCCAATTGCAGGCTCAAGAGGTC 1315
NF-6            CAGAGACTAATCCCTTCCAGAACTGATATTTTCCCTTCCCAATTGCAGGCTCAAGAGGTC 1315
NF-9            CAGAGACTAATCCCTTCCAGAACTGATATTTTCCCTTCCCAATTGCAGGCTCAAGAGGTC 1315
NF-7            CAGAGACTAATCCCTTCCAGAACTGATATTTTCCCTTCCCAATTGCAGGCTCAAGAGGTC 1315
NF-1            CAGAGACTAATCCCTTCCAGAACTGATATTTTCCCTTCCCAATTGCAGGCTCAAGAGGTC 1315
NF-5            CAGAGACTAATCCCTTCCAGAACTGATATTTTCCCTTCCCAATTGCAGGCTCAAGAGGTC 1315
NF-4            CAGAGACTAATCCCTTCCAGAACTGATATTTTCCCTTCCCAATTGCAGGCTCAAGAGGTC 1315
NF-2            CAGAGGCTAATCCCTTCCAGAACTGATATTTTCCCTTCCCAATTGCAGGCTCAAGAGGTC 1320
AF-1            CAGAGACTAATCCCTTCCAGAACTGATATTTTCCCTTCCCAATTGCAGGCTCAAGAGGTC 1315
AF-3            CAGAGACTAATCCCTTCCAGAACTGATATTTTCCCTTCCCAATTGCAGGCTCAAGAGGTC 1306
AF-9            CAGAGACTAATCCCTTCCAGAACTGATATTTTCCCTTCCCAATTGCAGGCTCAAGAGGTC 1306
AF-8            CAGAGACTAATCCCTTCCAGAACTGATATTTTCCCTTCCCAATTGCAGGCTCAAGAGGTC 1306
AF-6            CAGAGACTAATCCCTTCCAGAACTGATATTTTCCCTTCCCAATTGCAGGCTCAAGAGGTC 1306
AF-2            CAGAGGCTAATCCCTTCCAGAACTGATATTTTCCCTTCCCAATTGCAGGCTCAAGAGGTC 1311
AF-10           CAGAGGCTAATCCCTTCCAGAACTGATATTTTCCCTTCCCAATTGCAGGCTCAAGAGGTC 1311
AF-7            CAGAGGCTAATCCCTTCCAGAACTGATATTTTCCCTTCCCAATTGCAGGCTCAAGAGGTC 1311
AF-5            CAGAGGCTAATCCCTTCCAGAACTGATATTTTCCCTTCCCAATTGCAGGCTCAAGAGGTC 1311
AF-4            CAGAGGCTAATCCCTTCCAGAACTGATATTTTCCCTTCCCAATTGCAGGCTCAAGAGGTC 1311
NF-10           CAGAGGCTAATCCCTTCCAGAACTGATATTTTCCCTTCCCAATTGCAGGCTCAAGAGGTC 1311
NF-3            CAGAGGCTAATCCCTTCCAGAACTGATATTTTCCCTTCCCAATTGCAGGCTCAAGAGGTC 1311
                ***** ******************************************************

NF-8            CACGCCTTCCTGCGCCAGTGGCTGAGCGACAACATCTCCAAGGAGGTGTCCGCCAGCCTG 1375
NF-6            CACGCCTTCCTGCGCCAGTGGCTGAGCGACAACATCTCCAAGGAGGTGTCCGCCAGCCTG 1375
NF-9            CACGCCTTCCTGCGCCAGTGGCTGAGCGACAACATCTCCAAGGAGGTGTCCGCCAGCCTG 1375
NF-7            CACGCCTTCCTGCGCCAGTGGCTGAGCGACAACATCTCCAAGGAGGTGTCCGCCAGCCTG 1375
NF-1            CACGCCTTCCTGCGCCAGTGGCTGAGCGACAACATCTCCAAGGAGGTGTCCGCCAGCCTG 1375
NF-5            CACGCCTTCCTGCGCCAGTGGCTGAGCGACAACATCTCCAAGGAGGTGTCCGCCAGCCTG 1375
NF-4            CACGCCTTCCTGCGCCAGTGGCTGAGCGACAACATCTCCAAGGAGGTGTCCGCCAGCCTG 1375
NF-2            CACGCCTTCCTGCGCCAGTGGCTGAGCGACAACATCTCCAAGGAGGTGTCCGCCAGCCTG 1380
AF-1            CACGCCTTCCTGCGCCAGTGGCTGAGCGACAACATCTCCAAGGAGGTGTCCGCCAGCCTG 1375
AF-3            CACGCCTTCCTGCGCCAGTGGCTGAGCGACAACATCTCCAAGGAGGTGTCCGCCAGCCTG 1366
AF-9            CACGCCTTCCTGCGCCAGTGGCTGAGCGACAACATCTCCAAGGAGGTGTCCGCCAGCCTG 1366
AF-8            CACGCCTTCCTGCGCCAGTGGCTGAGCGACAACATCTCCAAGGAGGTGTCCGCCAGCCTG 1366
AF-6            CACGCCTTCCTGCGCCAGTGGCTGAGCGACAACATCTCCAAGGAGGTGTCCGCCAGCCTG 1366
AF-2            CACGCCTTCCTGCGCCAGTGGCTGAGCGACAACATCTCCAAGGAGGTGTCCGCCAGCCTG 1371
AF-10           CACGCCTTCCTGCGCCAGTGGCTGAGCGACAACATCTCCAAGGAGGTGTCCGCCAGCCCG 1371
AF-7            CACGCCTTCCTGCGCCAGTGGCTGAGCGACAACATCTCCAAGGAGGTGTCCGCCAGCCTG 1371
AF-5            CACGCCTTCCTGCGCCAGTGGCTGAGCGACAACATCTCCAAGGAGGTGTCCGCCAGCCTG 1371
AF-4            CACGCCTTCCTGCGCCAGTGGCTGAGCGACAACATCTCCAAGGAGGTGTCCGCCAGCCTG 1371
NF-10           CACGCCTTCCTGCGCCAGTGGCTGAGCGACAACATCTCCAAGGAGGTGTCCGCCAGCCTG 1371
NF-3            CACGCCTTCCTGCGCCAGTGGCTGAGCGACAACATCTCCAAGGAGGTGTCCGCCAGCCTG 1371
                ********************************************************** *

NF-8            CGCATCCAGTACGGTGGATCCGTGACCGCCGCCAACGCCAAGGAGCTGGCCAAGAAGCCC 1435
NF-6            CGCATCCAGTACGGTGGATCCGTGACCGCCGCCAACGCCAAGGAGCTGGCCAAGAAGCCC 1435
NF-9            CGCATCCAGTACGGTGGATCCGTGACCGCCGCCAACGCCAAGGAGCTGGCCAAGAAGCCC 1435
NF-7            CGCATCCAGTACGGTGGATCCGTGACCGCCGCCAACGCCAAGGAGCTGGCCAAGAAGCCC 1435
NF-1            CGCATCCAGTACGGTGGATCCGTGACCGCCGCCAACGCCAAGGAGCTGGCCAAGAAGCCC 1435
NF-5            CGCATCCAGTACGGTGGATCCGTGACCGCCGCCAACGCCAAGGAGCTGGCCAAGAAGCCC 1435
NF-4            CGCATCCAGTACGGTGGATCCGTGACCGCCGCCAACGCCAAGGAGCTGGCCAAGAAGCCC 1435
NF-2            CGCATCCAGTACGGTGGATCCGTGACCGCCGCCAACGCCAAGGAGCTGGCCAAGAAGCCC 1440
```

```
AF-1            CGCATCCAGTACGGTGGATCCGTGACCGCCGCCAACGCCAAGGAGCTGGCCAAGAAGCCC 1435
AF-3            CGCATCCAGTACGGTGGATCCGTGACCGCCGCCAACGCCAAGGAGCTGGCCAAGAAGCCC 1426
AF-9            CGCATCCAGTACGGTGGATCCGTGACCGCCGCCAACGCCAAGGAGCTGGCCAAGAAGCCC 1426
AF-8            CGCATCCAGTACGGTGGATCCGTGACCGCCGCCAACGCCAAGGAGCTGGCCAAGAAGCCC 1426
AF-6            CGCATCCAGTACGGTGGATCCGTGACCGCCGCCAACGCCAAGGAGCTGGCCAAGAAGCCC 1426
AF-2            CGCATCCAGTACGGTGGATCCGTGACCGCCGCCAACGCCAAGGAGCTGGCCAAGAAGCCC 1431
AF-10           CGCATCCAGTACGGTGGATCCGTGACCGCCGCCAACGCCAAGGAGCTGGCCAAGAAGCCC 1431
AF-7            CGCATCCAGTACGGTGGATCCGTGACCGCCGCCAACGCCAAGGAGCTGGCCAAGAAGCCC 1431
AF-5            CGCATCCAGTACGGTGGATCCGTGACCGCCGCCAACGCCAAGGAGCTGGCCAAGAAGCCC 1431
AF-4            CGCATCCAGTACGGTGGATCCGTGACCGCCGCCAACGCCAAGGAGCTGGCCAAGAAGCCC 1431
NF-10           CGCATCCAGTACGGTGGATCCGTGACCGCCGCCAACGCCAAGGAGCTGGCCAAGAAGCCC 1431
NF-3            CGCATCCAGTACGGTGGATCCGTGACCGCCGCCAACGCCAAGGAGCTGGCCAAGAAGCCC 1431
                ************************************************************

NF-8            GACATCGATGGCTTCCTGGTCGGAGGCGCCTCCCTGAAGCCCGAGTTCGTGGACATCATC 1495
NF-6            GACATCGATGGCTTCCTGGTCGGAGGCGCCTCCCTGAAGCCCGAGTTCGTGGACATCATC 1495
NF-9            GACATCGATGGCTTCCTGGTCGGAGGCGCCTCCCTGAAGCCCGAGTTCGTGGACATCATC 1495
NF-7            GACATCGATGGCTTCCTGGTCGGAGGCGCCTCCCTGAAGCCCGAGTTCGTGGACATCATC 1495
NF-1            GACATCGATGGCTTCCTGGTCGGAGGCGCCTCCCTGAAGCCCGAGTTCGTGGACATCATC 1495
NF-5            GACATCGATGGCTTCCTGGTCGGAGGCGCCTCCCTGAAGCCCGAGTTCGTGGACATCATC 1495
NF-4            GACATCGATGGCTTCCTGGTCGGAGGCGCCTCCCTGAAGCCCGAGTTCGTGGACATCATC 1495
NF-2            GACATCGATGGCTTCCTGGTCGGAGGCGCCTCCCTGAAGCCCGAGTTCGTGGACATCATC 1500
AF-1            GACATCGATGGCTTCCTGGTCGGAGGCGCCTCCCTGAAGCCCGAGTTCGTGGACATCATC 1495
AF-3            GACATCGATGGCTTCCTGGTCGGAGGCGCCTCCCTGAAGCCCGAGTTCGTGGACATCATC 1486
AF-9            GACATCGATGGCTTCCTGGTCGGAGGCGCCTCCCTGAAGCCCGAGTTCGTGGACATCATC 1486
AF-8            GACATCGATGGCTTCCTGGTCGGAGGCGCCTCCCTGAAGCCCGAGTTCGTGGACATCATC 1486
AF-6            GACATCGATGGCTTCCTGGTCGGAGGCGCCTCCCTGAAGCCCGAGTTCGTGGACATCATC 1486
AF-2            GACATCGATGGCTTCCTGGTCGGAGGCGCCTCCCTGAAGCCCGAGTTCGTGGACATCATC 1491
AF-10           GACATCGATGGCTTCCTGGTCGGAGGCGCCTCCCTGAAGCCCGAGTTCGTGGACATCATC 1491
AF-7            GACATCGATGGCTTCCTGGTCGGAGGCGCCTCCCTGAAGCCCGAGTTCGTGGACATCATC 1491
AF-5            GACATCGATGGCTTCCTGGTCGGAGGCGCCTCCCTGAAGCCCGAGTTCGTGGACATCATC 1491
AF-4            GACATCGATGGCTTCCTGGTCGGAGGCGCCTCCCTGAAGCCCGAGTTCGTGGACATCATC 1491
NF-10           GACATCGATGGCTTCCTGGTCGGAGGCGCCTCCCTGAAGCCCGAGTTCGTGGACATCATC 1491
NF-3            GACATCGATGGCTTCCTGGTCGGAGGCGCCTCCCTGAAGCCCGAGTTCGTGGACATCATC 1491
                ************************************************************

NF-8            AACGCCCGGCAGTAAGCAACGCGATCTCAAGCATCAGATCGCTTTCGGCCAGCGAACTGC 1555
NF-6            AACGCCCGGCAGTAAGCAACGCGATCTCAAGCATCAGATCGCTTTCGGCCAGCGAACTGC 1555
NF-9            AACGCCCGGCAGTAAGCAACGCGATCTCAAGCATCAGATCGCTTTCGGCCAGCGAACTGC 1555
NF-7            AACGCCCGGCAGTAAGCAACGCGATCTCAAGCATCAGATCGCTTTCGGCCAGCGAACTGC 1555
NF-1            AACGCCCGGCAGTAAGCAACGCGATCTCAAGCATCAGATCGCTTTCGGCCAGCGAACTGC 1555
NF-5            AACGCCCGGCAGTAAGCAACGCGATCTCAAGCATCAGATCGCTTTCGGCCAGCGAACTGC 1555
NF-4            AACGCCCGGCAGTAAGCAACGCGATCTCAAGCATCAGATCGCTTTCGGCCAGCGAACTGC 1555
NF-2            AACGCCCGGCAGTAAGCAACGCGATCTCAAGCATCAGATCGCTTTCGGCCAGCGAACTGC 1560
AF-1            AACGCCCGGCAGTAAGCAACGCGATCTCAAGCATCAGATCGCTTTCGGCCAGCGAACTGC 1555
AF-3            AACGCCCGGCAGTAAGCAACGCGATCTCAAGCATCAGATCGCTTTCGGCCAGCGAACTGC 1546
AF-9            AACGCCCGGCAGTAAGCAACGCGATCTCAAGCATCAGATCGCTTTCGGCCAGCGAACTGC 1546
AF-8            AACGCCCGGCAGTAAGCAACGCGATCTCAAGCATCAGATCGCTTTCGGCCAGCGAACTGC 1546
AF-6            AACGCCCGGCAGTAAGCAACGCGATCTCAAGCATCAGATCGCTTTCGGCCAGCGAACTGC 1546
AF-2            AACGCCCGGCAGTAAGCAACGCGATCTCAAGCATCAGATCGCTTTCGGCCAGCGAACTGC 1551
AF-10           AACGCCCGGCAGTAAGCAACGCGATCTCAAGCATCAGATCGCTTTCGGCCAGCGAACTGC 1551
AF-7            AACGCCCGGCAGTAAGCAACGCGATCTCAAGCATCAGATCGCTTTCGGCCAGCGAACTGC 1551
AF-5            AACGCCCGGCAGTAAGCAACGCGATCTCAAGCATCAGATCGCTTTCGGCCAGCGAACTGC 1551
AF-4            AACGCCCGGCAGTAAGCAACGCGATCTCAAGCATCAGATCGCTTTCGGCCAGCGAACTGC 1551
NF-10           AACGCCCGGCAGTAAGCAACGCGATCTCAAGCATCAGATCGCTTTCGGCCAGCGAACTGC 1551
NF-3            AACGCCCGGCAGTAAGCAACGCGATCTCAAGCATCAGATCGCTTTCGGCCAGCGAACTGC 1551
                ************************************************************

NF-8            CCGCGAAGGAGGCACTGGAGGGCAGGACTCCATACTCCTGCGCGCGAATGCATTACGCAC 1615
```

```
NF-6      CCGCGAAGGAGGCACTGGAGGGCAGGACTCCATACTCCTGCGCGCGAATGCATTACGCAC 1615
NF-9      CCGCGAAGGAGGCACTGGAGGGCAGGACTCCATACTCCTGCGCGCGAATGCATTACGCAC 1615
NF-7      CCGCGAAGGAGGCACTGGAGGGCAGGACTCCATACTCCTGCGCGCGAATGCATTACGCAC 1615
NF-1      CCGCGAAGGAGGCACTGGAGGGCAGGACTCCATACTCCTGCGCGCGAATGCATTACGCAC 1615
NF-5      CCGCGAAGGAGGCACTGGAGGGCAGGACTCCATACTCCTGCGCGCGAATGCATTACGCAC 1615
NF-4      CCGCGAAGGAGGCACTGGAGGGCAGGACTCCATACTCCTGCGCGCGAATGCATTACGCAC 1615
NF-2      CCGCGAAGGAGACACTGGAGGGCAGGACTCCATACTCCTGCGCGCGAATGCATTACGCAC 1620
AF-1      CCGCGAAGGAGGCACTGGAGGGCAGGACTCCATACTCCTGCGCGCGAATGCATTACGCAC 1615
AF-3      CCGCGAAGGAGGCACTGGAGGGCAGGACTCCATACTCCTGCGCGCGAATGCATTACGCAC 1606
AF-9      CCGCGAAGGAGGCACTGGAGGGCAGGACTCCATACTCCTGCGCGCGAATGCATTACGCAC 1606
AF-8      CCGCGAAGGAGGCACTGGAGGGCAGGACTCCATACTCCTGCGCGCGAATGCATTACGCAC 1606
AF-6      CCGCGAAGGAGGCACTGGAGGGCAGGACTCCATACTCCTGCGCGCGAATGCATTACGCAC 1606
AF-2      CCGCGAAGGAGACACTGGAGGGCAGGACTCCATACTCCTGCGCGCGAATGCATTACGCAC 1611
AF-10     CCGCGAAGGAGACACTGGAGGGCAGGACTCCATACTCCTGCGCGCGAATGCATTACGCAC 1611
AF-7      CCGCGAAGGAGACACTGGAGGGCAGGACTCCATACTCCTGCGCGCGAATGCATTACGCAC 1611
AF-5      CCGCGAAGGAGACACTGGAGGGCAGGACTCCATACTCCTGCGCGCGAATGCATTACGCAC 1611
AF-4      CCGCGAAGGAGACACTGGAGGGCAGGACTCCATACTCCTGCGCGCGAATGCATTACGCAC 1611
NF-10     CCGCGAAGGAGACACTGGAGGGCAGGACTCCATACTCCTGCGCGCGAATGCATTACGCAC 1611
NF-3      CCGCGAAGGAGACACTGGAGGGCAGGACTCCATACTCCTGCGCGCGAATGCATTACGCAC 1611
          *********** ************************************************

                     #
NF-8      ACACCTAGTTATTGTCACCACCAAGTGCAGTTCCAAATTCATAGTTACCTTGCGAGAATG 1675
NF-6      ACACCTAGTTATTGTCACCACCAAGTGCAGTTCCAAATTCATAGTTACCTTGCGAGAATG 1675
NF-9      ACACCTAGTTATTGTCACCACCAAGTGCAGTTCCAAATTCATAGTTACCTTGCGAGAATG 1675
NF-7      ACACCTAGTTATTGTCACCACCAAGTGCAGTTCCAAATTCATAGTTACCTTGCGAGAATG 1675
NF-1      ACACCTAGTTATTGTCACCACCAAGTGCAGTTCCAAATTCATAGTTACCTTGCGAGAATG 1675
NF-5      ACACCTAGTTATTGTCACCACCAAGTGCAGTTCCAAATTCATAGTTACCTTGCGAGAATG 1675
NF-4      ACACCTAGTTATTGTCACCACCAAGTGCAGTTCCAAATTCATAGTTACCTTGCGAGAATG 1675
NF-2      ACACCTAGTTGTTGTCACCACCAAGTGCAGTTCCAAATTCATAGTTACCTTGCGAGAATG 1680
AF-1      ACACCTAGTTGTTGTCACCACCAAGTGCAGTTCCAAATTCATAGTTACCTTGCGAGAATG 1675
AF-3      ACACCTAGTTGTTGTCACCACCAAGTGCAGTTCCAAATTCATAGTTACCTTGCGAGAATG 1666
AF-9      ACACCTAGTTGTTGTCACCACCAAGTGCAGTTCCAAATTCATAGTTACCTTGCGAGAATG 1666
AF-8      ACACCTAGTTGTTGTCACCACCAAGTGCAGTTCCAAATTCATAGTTACCTTGCGAGAATG 1666
AF-6      ACACCTAGTTGTTGTCACCACCAAGTGCAGTTCCAAATTCATAGTTACCTTGCGAGAATG 1666
AF-2      ACACCTAGTTGTTGTCACCACCAAGTGCAGTTCCAAATTCATAGTTACCTTGCGAGAATG 1671
AF-10     ACACCTAGTTGTTGTCACCACCAAGTGCAGTTCCAAATTCATAGTTACCTTGCGAGAATG 1671
AF-7      ACACCTAGTTGTTGTCACCACCAAGTGCAGTTCCAAATTCATAGTTACCTTGCGAGAATG 1671
AF-5      ACACCTAGTTGTTGTCACCACCAAGTGCAGTTCCAAATTCATAGTTACCTTGCGAGAATG 1671
AF-4      ACACCTAGTTGTTGTCACCACCAAGTGCAGTTCCAAATTCATAGTTACCTTGCGAGAATG 1671
NF-10     ACACCTAGTTGTTGTCACCACCAAGTGCAGTTCCAAATTCATAGTTACCTTGCGAGAATG 1671
NF-3      ACACCTAGTTGTTGTCACCACCAAGTGCAGTTCCAAATTCATAGTTACCTTGCGAGAATG 1671
          ********** *************************************************

NF-8      CTACTCACATCCTTGTTGTTGAACGTATAGTAGTCCATTGTGTAAATCTTAAATTATTCA 1735
NF-6      CTACTCACATCCTTGTTGTTGAACGTATAGTAGTCCATTGTGTAAATCTTAAATTATTCA 1735
NF-9      CTACTCACATCCTTGTTGTTGAACGTATAGTAGTCCATTGTGTAAATCTTAAATTATTCA 1735
NF-7      CTACTCACATCCTTGTTGTTGAACGTATAGTAGTCCATTGTGTAAATCTTAAATTATTCA 1735
NF-1      CTACTCACATCCTTGTTGTTGAACGTATAGTAGTCCATTGTGTAAATCTTAAATTATTCA 1735
NF-5      CTACTCACATCCTTGTTGTTGAACGTATAGTAGTCCATTGTGTAAATCTTAAATTATTCA 1735
NF-4      CTACTCACATCCTTGTTGTTGAACGTATAGTAGTCCATTGTGTAAATCTTAAATTATTCA 1735
NF-2      CTACTCACATCCTTGTTGTTGAACGTATAGTAGTCCATTGTGTAAATCTTAAATTATTCA 1740
AF-1      CTACTCACATCCTTGTTGTTGAACGTATAGTAGTCCATTGTGTAAATCTTAAATTATTCA 1735
AF-3      CTACTCACATCCTTGTTGTTGAACGTATAGTAGTCCATTGTGTAAATCTTAAATTATTCA 1726
AF-9      CTACTCACATCCTTGTTGTTGAACGTATAGTAGTCCATTGTGTAAATCTTAAATTATTCA 1726
AF-8      CTACTCACATCCTTGTTGTTGAACGTATAGTAGTCCATTGTGTAAATCTTAAATTATTCA 1726
AF-6      CTACTCACATCCTTGTTGTTGAACGTATAGTAGTCCATTGTGTAAATCTTAAATTATTCA 1726
AF-2      CTACTCACATCCTTGTTGTTGAACGTATAGTAGTCCATTGTGTAAATCTTAAATTATTCA 1731
AF-10     CTACTCACATCCTTGTTGTTGAACGTATAGTAGTCCATTGTGTAAATCTTAAATTATTCA 1731
```

```
AF-7       CTACTCACATCCTTGTTGTTGAACGTATAGTAGTCCATTGTGTAAATCTTAAATTATTCA 1731
AF-5       CTACTCACATCCTTGTTGTTGAACGTATAGTAGTCCATTGTGTAAATCTTAAATTATTCA 1731
AF-4       CTACTCACATCCTTGTTGTTGAACGTATAGTAGTCCATTGTGTAAATCTTAAATTATTCA 1731
NF-10      CTACTCACATCCTTGTTGTTGAACGTATAGTAGTCCATTGTGTAAATCTTAAATTATTCA 1731
NF-3       CTACTCACATCCTTGTTGTTGAACGTATAGTAGTCCATTGTGTAAATCTTAAATTATTCA 1731
           ************************************************************

NF-8       GGCAATAATTATGCGCTACTCATTTACTTATATACCAAACCAAACAGGTGTCTTATTTTT 1795
NF-6       GGCAATAATTATGCGCTACTCATTTACTTATATACCAAACCAAACAGGTGTCTTATTTTT 1795
NF-9       GGCAATAATTATGCGCTACTCATTTACTTATATACCAAACCAAACAGGTGTCTTATTTTT 1795
NF-7       GGCAATAATTATGCGCTACTCATTTACTTATATACCAAACCAAACAGGTGTCTTATTTTT 1795
NF-1       GGCAATAATTATGCGCTACTCATTTACTTATATACCAAACCAAACAGGTGTCTTATTTTT 1795
NF-5       GGCAATAATTATGCGCTACTCATTTACTTATATACCAAACCAAACAGGTGTCTTATTTTT 1795
NF-4       GGCAATAATTATGCGCTACTCATTTACTTATATACCAAACCAAACAGGTGTCTTATTTTT 1795
NF-2       GGCAATAATTATGCGCTACTCATTTACTTATATACCAAACCAAACAGGTGTCTTATTTTT 1800
AF-1       GGCAATAATTATGCGCTACTCATTTACTTATATACCAAACCAAACAGGTGTCTTATTTTT 1795
AF-3       GGCAATAATTATGCGCTACTCATTTACTTATATACCAAACCAAACAGGTGTCTTATTTTT 1786
AF-9       GGCAATAATTATGCGCTACTCATTTACTTATATACCAAACCAAACAGGTGTCTTATTTTT 1786
AF-8       GGCAATAATTATGCGCTACTCATTTACTTATATACCAAACCAAACAGGTGTCTTATTTTT 1786
AF-6       GGCAATAATTATGCGCTACTCATTTACTTATATACCAAACCAAACAGGTGTCTTATTTTT 1786
AF-2       GGCAATAATTATGCGCTACTCATTTACTTATATACCAAACCAAACAGGTGTCTTATTTTT 1791
AF-10      GGCAATAATTATGCGCTACTCATTTACTTATATACCAAACCAAACAGGTGTCTTATTTTT 1791
AF-7       GGCAATAATTATGCGCTACTCATTTACTTATATACCAAACCAAACAGGTGTCTTATTTTT 1791
AF-5       GGCAATAATTATGCGCTACTCATTTACTTATATACCAAACCAAACAGGTGTCTTATTTTT 1791
AF-4       GGCAATAATTATGCGCTACTCATTTACTTATATACCAAACCAAACAGGTGTCTTATTTTT 1791
NF-10      GGCAATAATTATGCGCTACTCATTTACTTATATACCAAACCAAACAGGTGTCTTATTTTT 1791
NF-3       GGCAATAATTATGCGCTACTCATTTACTTATATACCAAACCAAACAGGTGTCTTATTTTT 1791
           ************************************************************

                       #
NF-8       TGCAAGACTATTCCAGCTATTTTTAGAGCGATTGGTTGAACGACAAGGTGTCCAAAGTTG 1855
NF-6       TGCAAGACTATTCCAGCTATTTTTAGAGCGATTGGTTGAACGACAAGGTGTCCAAAGTTG 1855
NF-9       TGCAAGACTATTCCAGCTATTTTTAGAGCGATTGGTTGAACGACAAGGTGTCCAAAGTTG 1855
NF-7       TGCAAGACTATTCCAGCTATTTTTAGAGCGATTGGTTGAACGACAAGGTGTCCAAAGTTG 1855
NF-1       TGCAAGACTATTCCAGCTATTTTTAGAGCGATTGGTTGAACGACAAGGTGTCCAAAGTTG 1855
NF-5       TGCAAGACTATTCCAGCTATTTTTAGAGCGATTGGTTGAACGACAAGGTGTCCAAAGTTG 1855
NF-4       TGCAAGACTATTCCAGCTATTTTTAGAGCGATTGGTTGAACGACAAGGTGTCCAAAGTTG 1855
NF-2       TGCAAGACTATTTCAGCTATTTTTAGAGCGATTGGTTGAACGACAAGGTGTCCAAAGTTG 1860
AF-1       TGCAAGACTATTTCAGCTATTTTTAGAGCGATTGGTTGAACGACAAGGTGTCCAAAGTTG 1855
AF-3       TGCAAGACTATTTCAGATATTTTTAGAGCGATTGGTTGAACGACAAGGTGTCCAAAGTTG 1846
AF-9       TGCAAGACTATTTCAGATATTTTTAGAGCGATTGGTTGAACGACAAGGTGTCCAAAGTTG 1846
AF-8       TGCAAGACTATTTCAGATATTTTTAGAGCGATTGGTTGAACGACAAGGTGTCCAAAGTTG 1846
AF-6       TGCAAGACTATTTCAGATATTTTTAGAGCGATTGGTTGAACGACAAGGTGTCCAAAGTTG 1846
AF-2       TGCAAGACTGTTTCAGCTATTTTTAGAGCGATTGGTTGAACGACAAGGTGTCCAAAGTTG 1851
AF-10      TGCAAGACTGTTTCAGCTATTTTTAGAGCGATTGGTTGAACGACAAGGTGTCCAAAGTTG 1851
AF-7       TGCAAGACTGTTTCAGCTATTTTTAGAGCGATTGGTTGAACGACAAGGTGTCCAAAGTTG 1851
AF-5       TGCAAGACTGTTTCAGCTATTTTTAGAGCGATTGGTTGAACGACAAGGTGTCCAAAGTTG 1851
AF-4       TGCAAGACTGTTTCAGCTATTTTTAGAGCGATTGGTTGAACGACAAGGTGTCCAAAGTTG 1851
NF-10      TGCAAGACTATTTCAGCTATTTTTAGAGCGATTGGTTGAACGACAAGGTGTCCAAAGTTG 1851
NF-3       TGCAAGACTATTTCAGCTATTTTTAGAGCGATTGGTTGAACGACAAGGTGTCCAAAGTTG 1851
           ********* ** *** *******************************************

NF-8       TGTGCACATATGTACATATATGTGTCACCTCTGCGGACCT 1895
NF-6       TGTGCACATATGTACATATATGTGTCACCTCTGCGGACCT 1895
NF-9       TGTGCACATATGTACATATATGTGTCACCTCTGCGGACCT 1895
NF-7       TGTGCACATATGTACATATATGTGTCACCTCTGCGGACCT 1895
NF-1       TGTGCACATATGTACATATATGTGTCACCTCTGCGGACCT 1895
NF-5       TGTGCACATATGTACATATATGTGTCACCTCTGCGGACCT 1895
NF-4       TGTGCACATATGTACATATATGTGTCACCTCTGCGGACCT 1895
```

```
NF-2            TGTGCACATATGTACATATATGTGTCACCTCTGCGGACCT 1900
AF-1            TGTGCACATATGTACATATATGTGTCACCTCTGCGGACCT 1895
AF-3            TGTGCACATATGTACATATATGTGTCACCTCTGCGGACCT 1886
AF-9            TGTGCACATATGTACATATATGTGTCACCTCTGCGGACCT 1886
AF-8            TGTGCACATATGTACATATATGTGTCACCTCTGCGGACCT 1886
AF-6            TGTGCACATATGTACATATATGTGTCACCTCTGCGGACCT 1886
AF-2            TGTGCACATATGTACATATATGTGTCACCTCTGCGGACCT 1891
AF-10           TGTGCACATATGTACATATATGTGTCACCTCTGCGGACCT 1891
AF-7            TGTGCACATATGTACATATATGTGTCACCTCTGCGGACCT 1891
AF-5            TGTGCACATATGTACATATATGTGTCACCTCTGCGGACCT 1891
AF-4            TGTGCACATATGTACATATATGTGTCACCTCTGCGGACCT 1891
NF-10           TGTGCACATATGTACATATATGTGTCACCTCTGCGGACCT 1891
NF-3            TGTGCACATATGTACATATATGTGTCACCTCTGCGGACCT 1891
                ****************************************
```
